# Seasonal variations affect the ecosystem functioning and microbial assembly processes in plantation forest soils

**DOI:** 10.3389/fmicb.2024.1391193

**Published:** 2024-07-26

**Authors:** Min Wang, Abolfazl Masoudi, Can Wang, Liqiang Zhao, Jia Yang, Zhijun Yu, Jingze Liu

**Affiliations:** ^1^Hebei Key Laboratory of Animal Physiology, Biochemistry, and Molecular Biology, Hebei Collaborative Innovation Center for Eco-Environment, Hebei Research Center of the Basic Discipline of Cell Biology, Ministry of Education Key Laboratory of Molecular and Cellular Biology, College of Life Sciences, Hebei Normal University, Shijiazhuang, China; ^2^Department of Biological Sciences, University of Illinois, Chicago, IL, United States

**Keywords:** seasonal microbial assembly, microbial diversity, multifunctionality, biogeochemical cycling genes, co-occurrence networks

## Abstract

While afforestation mitigates climate concerns, the impact of afforestation on ecological assembly processes and multiple soil functions (multifunctionality) in afforested areas remains unclear. The Xiong’an New Area plantation forests (*Pinus* and *Sophora* forests) in North China were selected to examine the effects of plantation types across four distinct seasons on soil microbiomes. Three functional categories (nutrient stocks, organic matter decomposition, and microbial functional genes) of multifunctionality and the average (net) multifunctionality were quantified. All these categories are directly related to soil functions. The results showed that net soil multifunctionality as a broad function did not change seasonally, unlike other narrow functional categories. Bacterial communities were deterministically (variable selection and homogenous selection) structured, whereas the stochastic process of dispersal limitation was mainly responsible for the assembly and turnover of fungal and protist communities. In *Pinus* forests, winter initiates a sudden shift from deterministic to stochastic processes in bacterial community assembly, accompanied by decreased Shannon diversity and heightened nutrient cycling (nutrient stocks and organic matter decomposition). This indicates the potential vulnerability of deterministic assembly to seasonal fluctuations, particularly in environments rich in nutrients. The results predicted that protist community composition was uniquely structured with C-related functional activities relative to bacterial and fungal β-diversity variations, which were mostly explained by seasonal variations. Our study highlighted the importance of the protist phagocytosis process on soil microbial interactions through the predicted impact of protist α-diversity on microbial cooccurrence network parameters. This association might be driven by the high abundance of protist consumers as the main predators of bacterial and fungal lineages in our sampling plots. Our findings reveal that the complexity of microbial co-occurrence interactions was considerably higher in spring, perhaps attributing thermal variability and increased resource availability within spring that foster microbial diversity and network complexity. This study contributes to local ecosystem prospects to model the behavior of soil biota seasonally and their implied effects on soil functioning and microbial assembly processes, which will benefit global-scale afforestation programs by promoting novel, precise, and rational plantation forests for future environmental sustainability and self-sufficiency.

## 1 Introduction

The composition and function of microbial communities within terrestrial ecosystems can be changed greatly by anthropogenic activities, leading to notable alterations in multiple ecosystem functions (multifunctionality) (Fu et al., 2020). Soil multifunctionality refers to the ability of soils to simultaneously provide multiple ecosystem services and functions that are essential for environmental sustainability and human well-being (Zheng et al., 2019). Understanding soil functionality is crucial for achieving soil security by comprehensively recognizing the variability in soil processes, the ecosystem services soils provide to humans, and their responses to global changes (Chen et al., 2020). The interconnected and interdependent components of soil, such as its structure, chemistry, microbes, water, roots, and organic matter, are fundamental to enhancing and sustaining its multifunctionality (Yang et al., 2021). Vegetation restoration through afforestation occurs in different types of land use and climatic zones, either through self-generation or by establishing fast-growing trees with various physiological characteristics, which in turn can help reduce the effects of climate change (Hou et al., 2019). The largest afforestation area is currently recorded in China (Wang et al., 2020b), and the widespread planting of forest trees such as *Pinus*, a type of evergreen conifer (Tang et al., 2007; Wang et al., 2020a) and *Sophora*, a deciduous legume, (Liu et al., 2018) has recently been typical for afforestation in the northern part of China (Wang et al., 2021). The interaction of tree species with soil microbes in forest plantations can significantly affect soil composition and ecosystem dynamics. Notably, *Pinus* forests and *Sophora* forests may interact differentially with soil microbes, potentially shaping their composition in contrasting ways. In line with this concept, the accumulation of microbial residues in the soil was found to be higher under certain tree species due to direct impacts on microbial residue accumulation through alteration of fungal biomass (Jing et al., 2023). Moreover, in northeast China, the afforestation of abandoned land with Chinese pine trees and deciduous legumes has been shown to influence soil properties and microbial communities, with the potential for diverse ecological outcomes (Yang et al., 2018b). It is also noted that different tree species can cause shifts in the microbial biomass, the quantities of culturable bacteria and Actinomyces, and the profiles of phospholipid fatty acids (PLFA), resulting in altered soil microbial community structures (Xia et al., 2012). The establishment of specific tree species, such as thorny bamboo, has been found to enhance microbial community diversity and activity by increasing soil organic matter (Chang et al., 2019). The spatial positioning of trees within mixed stands of coniferous and broad-leaved trees correlates with microbial properties, where microbial biomass may decrease with some coniferous influences but increase with broad-leaved tree influences—demonstrating the intricate relationships between tree species composition and microbial dynamics in the soil (Saetre, 1999). Afforestation with sustainable management stands out as the most cost-effective mitigation option in forestry, offering flexibility to lower carbon intensity and combat climate change (Wang et al., 2020b). Therefore, it is crucial to understand the role of ecological processes, both deterministic and stochastic, in the assembly of microbial communities and their links to multifunctionality in environments like afforested areas, which are initially subjected to intense human activities. To reach this goal, it is essential to focus on both plantation types and seasonal successions at the same study sites when trying to disentangle the drivers of microbial distributions, functions, and their interactions in plantation forest regions.

One of the fundamental aims of microbial ecology is to disclose the role of ecological processes and environmental parameters in driving the assembly of microbial communities (Stegen et al., 2012). The assembly of microbial species is generally structured by four classes of ecological processes, categorized into stochasticity (homogenizing dispersal and dispersal limitation) and determinism (homogeneous selection and variable selection) (Jia et al., 2018). Growing evidence suggests possible associations among microbial community assembly, composition, and ecosystem functioning (Liu et al., 2021). External driving determinants, such as the perturbance of land-use management using exotic vegetation, drive diversified ecosystem functions by changing assembly processes and microbiome dynamics. Subsequently, the outcomes will affect microbial community composition by imposing feedback (Stegen et al., 2018). However, the temporal patterns of stochastic versus deterministic assembly processes and the driving environmental parameters that seasonally influence ecosystem functions in plantation forests remain unresolved.

Seasonal variations induce multifaceted impacts on ecosystem functioning and microbial assembly processes in plantation forests (Wang et al., 2020a; Van Nuland et al., 2021). These effects range from alterations in microbial community dynamics due to shifts in litter quality and tree root activity to more direct influences on soil microbial properties, enzyme activities, and the interaction with plant growth cycles (Lin et al., 2023). This can lead to shifts in microbial community composition and function, affecting ecosystem functioning on a landscape-level scale (Myers et al., 2001). For example, in *Eucalyptus grandis* plantations, soil microbial properties have been shown to decrease initially upon land-use change but then recover to levels comparable to native forests after 4 years, but this pattern varies with the season, highlighting the seasonal influence on microbial dynamics (Xu et al., 2020). Another aspect of seasonal impact is the fluctuation of environmental variables. For instance, increased levels of microbial PLFAs and enzyme activities have been observed during the wet season compared to the dry season, indicating that moisture is a key factor in modulating soil microbial community structure and activity (Yang et al., 2018a). Additionally, seasonal shifts in plant carbon allocation between root and shoot systems alter nutrient availability through root exudates, leading to changes in microbial communities surrounding host tree roots throughout the growing season (Habiyaremye et al., 2020). Moreover, the significance of seasonality varies with vegetation type, which differs across plantation forests (Körner, 2007). For example, deciduous forests exhibit photosynthetic activity only during the growing season, with a brief period of leaf litter in autumn. In contrast, coniferous forests have an extended growing season and produce more resilient litter compared to deciduous vegetation (Crow et al., 2009; Voříšková et al., 2014). Therefore, comprehensive studies that account for both the taxonomic and functional traits of soil microbial communities across different vegetation types, particularly in plantation forests, while considering seasonal dynamics, can provide valuable insights for refining simulation models and developing effective climate change mitigation strategies.

In this study, soil samples were collected from two different plantation forest types (evergreen vs. deciduous; *Pinus tabulaeformis* Carr. and *Sophora japonica* L.) in which soil experienced considerable disturbance by human activities during land preparation during four distinct seasons. The diversity and composition of soil biota (bacteria, fungi, and protists), the gene copy numbers of soil biota, eco-enzymatic activities and stoichiometry, physiochemical properties, and the abundance of microbial functional genes involved in the transformation of C, N, P, and S as microbial functional attributes were characterized. Quantitative microbial element cycling (QMEC), based on high-throughput quantitative polymerase chain reaction (PCR) (Zheng et al., 2018) was used to explore (i) the relationship between microbial diversity and composition under different plantation types seasonally with the provision of soil multifunctional services and (ii) the microbial assembly processes in plantation forests in the XNA. Additionally, because protists play a number of important roles in soil food webs and their relationships are indispensable to various soil functions and ecosystem interactions such as nutrient cycles, organic matter disintegration, formation of soil architecture, pest control, pollutant removal, and regulation of various contaminants (Nguyen et al., 2023); thus, the impact of seasonal fluctuations on protists trophic level were investigated. This study presents a framework to enhance our comprehension of the mechanisms directing the microbial pattern, the benefaction of deterministic and stochastic processes to community assembly processes, and soil multifunctionality.

## 2 Materials and methods

### 2.1 Study area and soil sampling

The Xiong’an New Area (XNA) (38°43′–39°10′ N, 115°38′–116°20′ E), which includes three counties (Xiong, Anxin, and Rongcheng), is located in the north of China, which was established in 2017, and it is another new national city after the Shenzhen Special Economic Zone and Shanghai Pudong New District (Wang et al., 2020a, 2023) (Supplementary Figure S1). Between 2017 and 2020, Xiong’an added more than 27,000 hectares of trees, increasing its forest coverage to 30 percent^1^. This area is classified as a warm temperate continental monsoon climate with four distinct seasons (Yang et al., 2022). The average annual temperature and precipitation are approximately 12.1°C and 560 mm, respectively (Wang et al., 2022). Seasonal temperature and precipitation details are presented in Supplementary Table S1. The soil samples were collected from *P. tabulaeformis* plots (Chinese pine tree, hereinafter referred to as “CP”) and *S. japonica* plots (Chinese scholar tree, hereinafter referred to as “CS”) in plantation forests with a history of planting either *Zea mays* L. or *Triticum aestivum* L., followed by the closing of the land for afforestation (Wang et al., 2021). These species were sampled two years post afforestation age, and the approximate age of the trees at the time of sampling was four years. The time frame for soil sampling was arranged considering four different seasons. Seasonal samplings were performed as follows: in July 2019 (summer: plant growth period and rhizodeposition; hereinafter referred to as “SU”), in October 2019 (autumn; during the late phase of litterfall; hereinafter referred to as “AU”), in January 2020 (winter; snow-covered time and carbon polymers/phenolics; hereinafter referred to as “WI”), and in May 2020 (spring: three weeks after the emergence of leaves; hereinafter referred to as “SP”). The time-scale sampling was selected according to the weather climate of the northern part of China. Sampling details were followed according to the previous study with a few modifications (Masoudi et al., 2018). Briefly, three random lines were randomly selected with the distance between each line at approximately 100 m, and by walking along each line, a soil core was collected every 10 m (a total of 5 cores in each line). The resulting soil cores were mixed to yield a composite sample from each line; three samples were collected per plot for each season. Thus, we collected 120 soil subsamples up to 30 cm depth in the root-zone soil from defined experimental plots over 1 year. After each set of sample collections, visible grassroots and pebbles were removed. All soil samples were divided into two parts; the first part (~10 g) was immediately frozen at −20°C using a portable refrigerator (Foshan Aikai Electric Appliance Co., Ltd., Guangdong, China) for DNA extraction and was stored at −80°C after the samples were transferred to the laboratory. The second part (~500 g) was considered for geochemical measurements.

### 2.2 Soil physicochemical properties, extracellular enzyme activities, and enzyme stoichiometry

The soil water content (SWC), total carbon (TC), soil electrical conductivity (EC), soil pH, soil organic matter (SOM), total potassium (TK), rapidly available potassium (RAK), slow-available potassium (SAK), total phosphorus (TP), total nitrogen (TN), soil hydrolyzed nitrogen (HN), inorganic phosphorus (IP), organic phosphorus (OP), available phosphorus (AP), total sulfur (TS), and available sulfur (AS) were determined, and the detailed methods can be found in Supplementary Table S2. The ecoenzymatic activities (EEAs) involved in hydrolytic activities, such as carbon (β-glucosidase, BGC; β-xylosidase, BXYS; β-cellobiosidase, BCL), nitrogen (leucine aminopeptidase, LAP; ɑ-glucosidase, AGC; N-acetyl-β-D-glucosidase, NAG), phosphorus (alkaline phosphatase, ALP), sulfur (aryl sulfatase, ASF), and oxidative activities, such as phenolic compound oxidase, including polyphenol oxidase (PPO) and peroxidase (POD), were determined following the fluorescence-based protocols (Ullah et al., 2019) using soil enzyme kits purchased from Suzhou Comin Biotechnology Co., Ltd. (Suzhou, Jiangsu, China) (Supplementary Table S3), and reported in nmol h^−1^ g^−1^ units. Initially, 1 g of fresh soil was blended with 100 mL of sterilized water using a polytron homogenizer, ensuring a uniform suspension with the aid of a magnetic stirrer. This suspension, along with sterilized water, 200 μM of 4-methylumbelliferyl-linked substrates, and 10 μM of references, was dispensed into the wells of a black 96-well microplate. The microplates were covered and incubated at 25°C in darkness for 4 h. Following incubation, 10 μL of a 1 M NaOH solution was swiftly added to each well to halt the enzymatic reaction. Fluorescence readings were taken using a microplate fluorometer (Scientific Fluoroskan Ascent FL, Thermo Fisher Scientific, Waltham, MA, United States) equipped with 365 nm excitation and 450 nm emission filters. Additionally, PO and PEO were quantified via colorimetric methods in a clear 96-well microplate, as detailed in Ullah et al. (2019). The formulas ln (BGC): ln (NAG + LAP), ln (BGC): ln (ALP), and ln (NAG + LAP): ln (ALP) were used to calculate the ecoenzymatic stoichiometry (EES) C:N, C:P, and N:P activity ratios, respectively, to gain a better understanding of possible resource shifts with seasonal variations.

### 2.3 DNA extraction and amplicon sequencing

Soil genomic DNA extraction and quality checking were performed according to our previous publications (Masoudi et al., 2020). PCR amplification and sequencing were individually performed for each replicate. Target genes were bacterial 16S rRNA, fungal ITS, and protist 18S rRNA. The primers 338F/806R, 1737F/2043R, and TAReuk454FWD1F/TAReukREV3R were used to amplify the V3-V4 region of the bacterial 16S rRNA gene, fungal ITS1, and 18S rRNA, respectively. PCRs, amplifications, and sequencing were performed in the framework of our previous study (Wang et al., 2022). Microbial biomass was determined using a real-time quantitative PCR assay for the 16S rDNA, the ITS1 region, and 18S rDNA for bacteria, fungi, and protists according to our previous protocol (Wang et al., 2021). All qPCR reactions were performed on a CFX96™ Real-Time System (Bio-Rad Laboratories, Hercules, California, United States). Absolute quantitative qPCR results were used to express soil microbial biomass using 16S rDNA, the ITS1 region, and 18S rDNA for bacteria, fungi, and protists. Cycle threshold (Ct) values determined the amount of DNA templates in each sample. Standard curves for the three genes were produced by ligating the gene fragments to the T1 plasmid vector, which was then transformed into Trans1-T1 competent *Escherichia coli*. These gene fragments were quantified using a NanoDrop 2000 ultra-micro spectrophotometer (Thermo Fisher Scientific, United States) and diluted 10 times with ddH_2_O to generate the standard curves. Microbial biomass in different soil samples is expressed as log copy numbers of genes per gram of soil. The abundance and diversity of the functional genes involved in C, N, P, and S cycling in different plantation plots and seasons were estimated using quantitative microbial element cycling (QMEC) based on high-throughput quantitative PCR (HT-qPCR). QMEC, a chip utilizing qPCR technology, comprises 71 primers targeting bacterial CNPS (carbon, nitrogen, phosphorus, sulfur) cycling genes, along with one primer for the 16S rRNA gene (Supplementary Table S5). This innovative platform allows for the simultaneous qualitative and quantitative analysis of 72 genes across 72 samples in a single run on the WaferGen Smart-Chip Real-time PCR system (Bio-Rad, Hercules, CA, United States). The relative copy number of detected genes was determined following the method outlined by Zheng et al. (2018). Each qPCR reaction was carried out in a 100 μL reaction volume, with triplicate reactions performed for each primer set. A negative control (sterile water) was included in each run to monitor for contamination. The PCR amplification protocol consisted of an initial denaturation step at 95°C for 10 min, followed by 40 cycles of denaturation at 95°C for 30 s, annealing at 58°C for 30 s, and extension at 72°C for 30 s. The melting curve analysis was automatically generated using the WaferGen software, and the corresponding threshold cycle (Ct) value was determined. Any signals exhibiting multiple melting peaks or amplification efficiencies outside the range of 1.8 to 2.2 were excluded from the analysis. A Ct value of 31 served as the detection limit for gene presence. All reactions (Real-Time qPCR and HT-qPCR) were carried out in three technical replicates for each soil replicate (In total, nine replicates for three soil samples of each experimental plot) to ensure accuracy.

### 2.4 Bioinformatic and statistical analyses

Demultiplexing and quality filtering of raw FASTQ files were carried out according to our previous studies (Wang et al., 2022). The classified protistan OTUs were generally assigned to three different trophic levels, namely phototroph, consumer, or parasite, based on our previous study (Wang et al., 2021). Another group of trophic levels, photophagatroph, was considered for those protistan lineages that belonged to the sub-kingdom of Hacrobia (kingdom: Chromista) based on the study of Cavalier-Smith et al. (2015). Co-occurrence networks were used to uncover the potential interactions among bacterial, fungal, and protistan taxa in different seasons and plantations. Based on the “betweenness centrality score”, the top-ten nodes from each network were selected as key nodes (Wang et al., 2021). The α- and β diversity indices of bacterial, fungal, and protistan communities among the four seasons were measured based on our previous publication (Wang et al., 2022). To determine the relative contributions of environmental variables to change in soil microbial community compositions, PERMANOVA (permutational multivariate analysis of variance) was performed based on the function of “adonis” (analysis of similarity) using the package “vegan” in R. To calculate the predictor variables for “seasonality” and “plantation type” in the PERMANOVA model, principal component analysis (PCA) was used to summarize variations in different seasonal and plant species compositions (Wang et al., 2021). Linear discriminant analysis effect size, LEfSe (from phylum to genus level), and the Venn diagram were performed to identify which microbial taxa were primary contributors to the differences in community compositions between plantation types with seasonal variations. Soil multifunctionality was assessed according to Ding and Eldridge (2021). Data on various aspects of soil attributes were collected, and these variables were classified into three categories: nutrient stocks (*n* = 16 attributes), organic matter decomposition (*n* = 10 attributes), and microbial functional genes (*n* = 6 attributes). These attributes are good proxies of processes driving soil biogeochemical cycling and are frequently used to assess the ecosystem’s multifunctionality of microbial communities (Supplementary Table S6) (Ding and Eldridge, 2021; Qiu et al., 2021). These standardized attributes within each functional category were then averaged to obtain a multifunctionality index for each group as a narrow functional index. The net soil multifunctionality index was calculated by averaging the values of all 32 standardized attributes as a broad functional category (Supplementary Table S6). This method has been widely used previously (Qiu et al., 2021). Even soil pH is essential in defining nutrient cycling and availability; however, soil pH was not included when calculating the four different multifunctionality indices because pH is a logarithmic scale (Qiu et al., 2021). RDA (redundancy analysis) and heatmap analyses were used to explore the relationships between the soil microbial community, environmental factors, co-occurrence network parameters, multifunctionality indices, and microbial βNTI values via the package “vegan” in R (Wang et al., 2024). The student’s *t*-test was used to compare the microbial community and environmental factors among four seasons and plant species by JMP ver. 14.3.0 for Windows (SAS Institute LLC., Cary, United States). The effect of edaphic factors/soil enzymes, annual air temperature (max, min, and average) changes, diversity indices, and multifunctionality indices on soil microbial diversity and richness was further quantified using response curves fitted by generalized additive models (GAM) utilizing the R package mgcv. It should be mentioned that “annual air temperature” is the best predictor for “soil temperature” as Dennis et al. (2019) suggested. The Spearman correlation coefficient was calculated to evaluate the association between co-occurrence network parameters and environmental variables to determine how different plantation types and seasonal successions shaped microbial co-occurrence network parameters. The method suggested by Stegen et al. (2012) was used to consolidate the use of phylogenetic inferences and null modeling approaches to quantify the importance of ecological processes (variable selection, homogeneous selection, dispersal limitation, homogenizing dispersal, and undominated processes) in stimulating the assembly of soil microbiomes at multiple time points (spring, summer, autumn, and winter) in our experimental plots.

## 3 Results

### 3.1 Changes in soil physicochemical properties, extracellular ecoenzymatic activities, and stoichiometry

The results of the edaphic conditions are summarized for different seasons specific to this investigation (Supplementary Table S7). Edaphic parameters were unsteady throughout the course of one year. Soils in spring and summer were relatively mesic and high in some elements, such as TP and TN, whereas soils were relatively high in TC, SOM, TK, SWC, and EC in autumn and winter. Soil pH was alkaline across our sampling plots; it decreased significantly between spring and winter in the CP plot but did not change significantly (*p* < 0.05, *t*-test) in the CS plot with seasonal variations. SWC was significantly affected by seasonal variations. Differences in the EEAs of the soils were observed based on seasonal variations and plantations (*p* < 0.05, Supplementary Table S7). EEAs results are categorized as organic matter decomposition (Supplementary Table S6). The various seasons and plantations significantly influenced the EESs in the soils. Our results showed that the BGC:LAP + NAG (C:N) activity ratio was not significantly different in the CP plot seasonally (*F*_3,11_ = 2.0222, *p* = 0.1894), whereas this ratio was significantly different between spring and other seasons in the CS plot (*F*_3,11_ = 5.0298, *p* = 0.0301). The BGC:ALP (C:P) activity ratio was highest in winter, and it was significantly different between summer and spring in the CP plot (*F*_3,11_ = 4.5608, *p* = 0.0383), whereas this ratio was not significantly different seasonally in the CS plot (*F*_3,11_ = 0.8182, *p* = 0.5193). The LAP + NAG: ALP (N:P) activity ratio was significantly different between winter and the other seasons in both experimental plots, CP (*F*_3,11_ = 7.6975, *p* = 0.0096) and CS (*F*_3,11_ = 6.3929, *p* = 0.0161) (Supplementary Tables S8–S11). More detailed results can be found in the Supplementary Tables S8–S11 and Supplementary Figures S2, S3.

### 3.2 Response of the composition of soil microbial communities to seasonal variations in different plots

**Figure 1:**
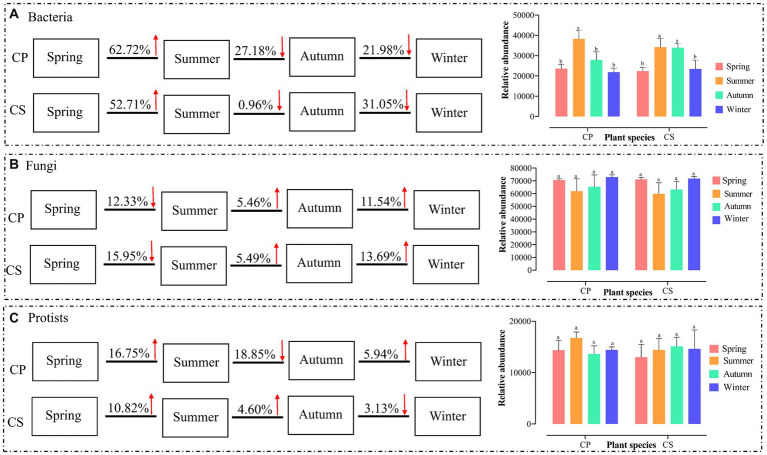
Seasonal trend of bacterial **(A)**, fungal **(B)**, and protistan **(C)** communities based on the relative abundance of microbial genera. The data represent the means of three replicates with standard errors. Significant differences (*t*-test, *p* < 0.05) among different seasons and plantations are labeled with different letters. CP, Chinese pine tree plots; CS, Chinese scholar tree plots.

**Figure 2:**
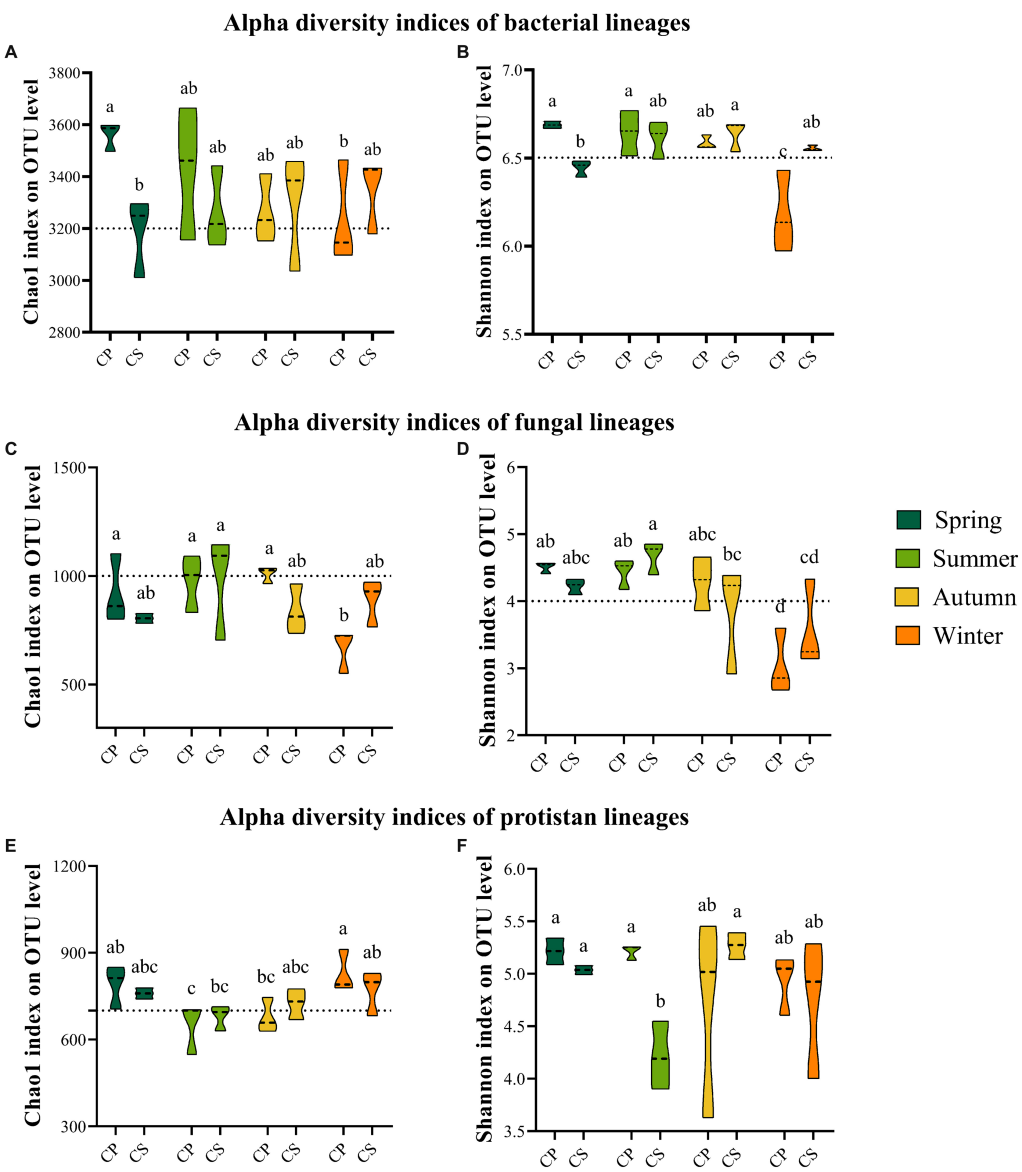
Alpha diversity indices of soil biota retrieved from two different forest types seasonally. Bacteria **(A,B)**, fungi **(C,D)**, and protists **(E,F)**. CP (*P. tabulaeformis*) and CS (*S. japonica*).

**Figure 3:**
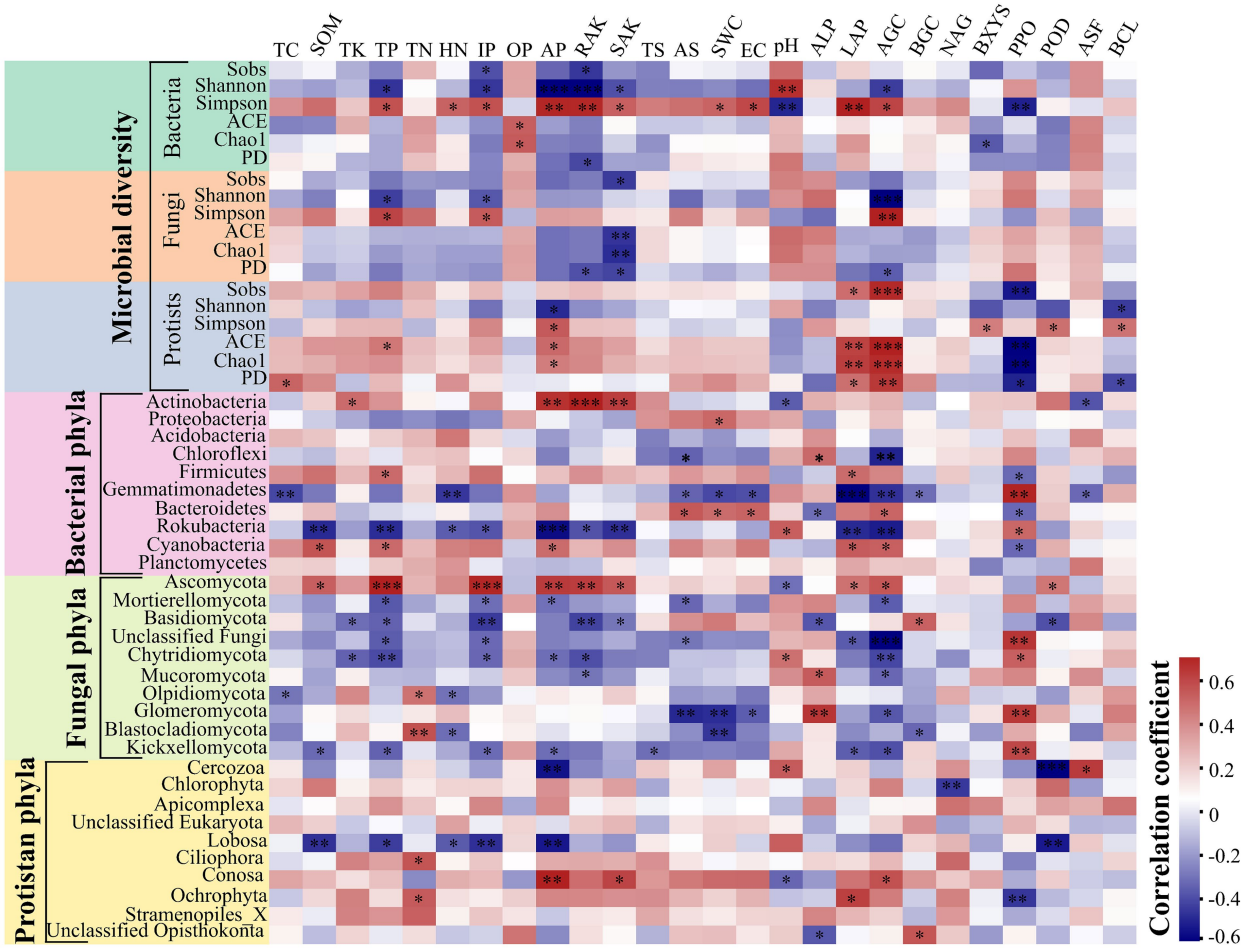
Spearman correlation ships between the relative abundance of dominated phyla and microbial diversity indices with edaphic physicochemical variables and soil enzymes. The red and blue colors indicate positive and negative correlations, respectively. The significances of correlation analyses are marked with asterisks (*) at different significance levels (*p < 0.05, **p < 0.01, and ***p < 0.001).

The core microbial communities, represented by all eight groups, showed that the core bacterial community comprised the highest OTUs across all samples (Supplementary Figure S4). The dominant Firmicutes phylum reached its peak abundance in winter in both plant communities, with significantly different levels observed between the two species plots (Supplementary Figures S5A,B and Supplementary Table S12). Similarly, the dominant Ascomycota exhibited its highest abundance in winter for both plant species, with no significant difference between spring and winter in the CS plot (Supplementary Figures S5C,D and Supplementary Table S12). Additionally, the protist communities were predominantly composed of Cercozoa (26.87%), Chlorophyta (14.51%), Apicomplexa (12.77%), unclassified Eukaryota (10.98%), and Lobosa (8.75%) (Supplementary Figures S5E,F). The disparity in bacterial abundance at the genus level widened between spring and summer in the CP (62.72%) and CS (52.71%) plots, while diminishing with seasonal transitions from summer to autumn and autumn to winter for both plant species (Figure 1A). Conversely, the relative abundance of fungal genera did not exhibit significant increases during seasonal shifts from summer to autumn and from autumn to winter in both plant species plots (Figure 1B) (Supplementary Figure S6B). Meanwhile, the abundance of protist genera escalated from spring to summer for both plant species, displaying divergent trends during seasonal changes from summer to autumn and autumn to winter across both plant types (Figure 1C). The study revealed significant differences in bacterial diversity (*F*_7,23_ = 6.8806, *p* = 0.0007) and richness (*F*_7,23_ = 3.4191, *p* = 0.026) between the two plant species during spring and winter (Figures 2A,B and Supplementary Table S13), with seasonal trends of other bacterial α-diversity indices summarized in Supplementary Figure S6. Additionally, fungal Shannon diversity (*F*_7,23_ = 4.4671, *p* = 0.0063) and richness (*F*_7,23_ = 2.7379, *p* = 0.0463) in the plant species plots significantly varied with seasonal changes, showing a decline in diversity and richness as the season transitioned to cold weather, particularly noticeable in the CP plot (Figure 2). Furthermore, protist richness (*F*_7,23_ = 2.7219, *p* = 0.0461) peaked in winter but did not significantly differ from other seasons, whereas protist Shannon diversity (*F*_7,23_ = 1.9374, *p* = 0.0486) did not follow this pattern (Figures 2E,F). Additional findings on microbial community compositions can be found in the Supplementary Figures S7–S9 and Supplementary Tables S14–S16. Although the microbial Shannon diversity was linked with several edaphic factors, such as soil pH, fewer soil factors showed a significant association with microbial richness (Chao1) (Figure 3). Four protistan alpha diversity indices (PD, Chao1, Sobs, and ACE) and one protist alpha diversity index (Simpson) were negatively and positively correlated with PPO and POD activities, respectively (Supplementary Figure S10). Quantitative PCR showed that the fungal biomass (gene copy number) was lower than that of other soil biota and followed the trend bacteria > protists > fungi (Supplementary Figure S11).

### 3.3 Drivers of soil microbiome community variations

The PERMANOVA showed that the impact of season on bacterial and fungal compositions was significant, explaining 26.83% (*p* = 0.029) and 43.30% (*p* = 0.002) of the bacterial and fungal variations, respectively (Supplementary Figures S12A,B and Supplementary Tables S17–S18). In contrast, the influence of season on protist compositions was not significant, explaining 17.91% (*p* = 0.163) of the variation in protists (Supplementary Figure S12C and Supplementary Table S19). The second most important factors for predicting the β-diversity of bacterial, fungal, and protist taxa were AGC, TP, and POD, which explained 17.02% (*p* = 0.011), 35.57% (*p* = 0.003), and 12.18% (*p* = 0.029), respectively, of the variance (Supplementary Figure S12C). Bacterial and fungal RDA analyses of soil enzymes did not separate hydrolytic and oxidative activities onto separate axes (Supplementary Figures S13A,C). However, interestingly, the RDA of soil enzymes condensed hydrolytic and oxidative activities onto separate axes (Supplementary Figure S13E). In addition, fungal RDA of edaphic parameters was much more concentric compared with bacterial and protist RDA analyses of soil properties (Supplementary Figures S13B,D,F).

### 3.4 Effect of seasonal variations and plantations on protistan trophic groups

The results showed that protist consumer taxa were the dominant group across our experimental plots (42.24%, on average). The second most dominant protist trophic group was phototrophs (eukaryotic algae, 31.68%), with the highest abundance in SWI samples and the lowest abundance in CSU samples. The protist communities were composed of taxa putatively assigned as 11.64% parasites, with the highest abundance in SSU samples and the lowest abundance in CSP samples. The photophagotroph group was found across sampling plots with a lower abundance compared with other trophic groups (0.35%, on average); the lowest abundance of this group was observed in CAT samples (0.15%), and the highest abundance was observed in SAT samples (0.53%). Across sampling plots, 14.07% of the overall protist communities were composed of the unclassified group (Supplementary Figure S14).

### 3.5 Co-occurrence patterns in microbial networks

**Figure 4:**
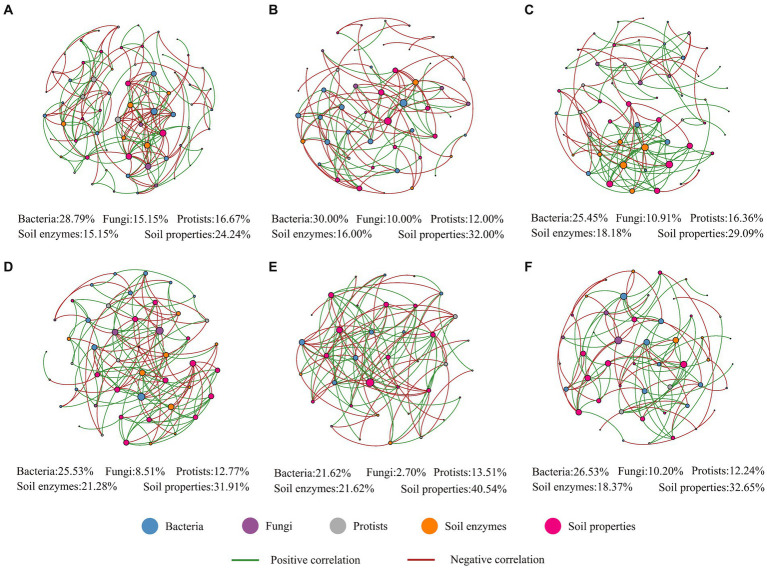
Bacterial, fungal, and protistan co-occurrence networks (at the family level) with environmental variables in the *Pinus* forest **(A)**, in the Sophora forest **(B)**, in spring **(C)**, in summer **(D)**, in autumn **(E)**, and winter **(F)**. The lines indicate interlinkage between OTUs. The green lines indicate a positive linkage, and the red lines indicate a negative linkage between OTUs.

To determine how plantation types and seasonality affected microbial network complexity, topological network parameters such as the “average degree (AD)”, “average clustering coefficient (ACC)”, and “average path length (APL)” were considered. Higher AD and ACC values indicate more complex networks. The more moderate the APL, the closer the association among the members. Applying this rationale and considering plantation type, our results indicated that the soil–biome relationship was more complex and intense in the *Pinus* forest; considering seasonality, the microbial co-occurrence network was more complex in spring, whereas this network was more intense in winter (Figure 4; Supplementary Table S20). Interestingly, the highest number of microbial links (bacteria to protists) was recorded in spring (Supplementary Table S20). Based on the key nodes, our results suggested that spring, which was characterized by those nodes, was affiliated with bacterial and protist networks and abiotic factors such as soil enzymes that belonged to the N and P cycles (Supplementary Table S21). More details of microbial interactions are presented in Supplementary Table S22. All network parameters were significantly associated with the three different classes of air temperature. Remarkably, oxidative enzymes such as PPO and N-related enzymes such as LAP were also associated with most network parameters (Supplementary Figure S15).

### 3.6 Seasonal impacts on microbial functional genes and soil functioning

Absolute functional gene abundance differed significantly (*p* < 0.05) among soil samples (Supplementary Figures S16A–C). More specifically, the results of QMEC based on the HT-qPCR approach showed that the abundance of those genes involved in C degradation (ANOVA, *p* = 0.0139), N cycling (ANOVA, *p* = 0.0026), and S cycling (ANOVA, *p* = 0.009) was significantly affected by plantation type seasonally (Supplementary Figure S16D). The PCA revealed that the CNPS cycling gene compositions in different plantation types with seasonal variations formed distinct but concentric clusters in which the CNPS cycling genes of the *Pinus* forest in autumn and winter separated from other clusters (Supplementary Figure S17). Additionally, a significant impact of different plantations with seasonal variations on the overall profile of CNPS cycling genes was observed (Adonis, *p* = 0.004, Anosim, *p* = 0.006, and MRPP, *p* = 0.008). The abundance of keystone C-hydrolysis genes significantly differed seasonally among plantation types, and the abundance of those genes involved in hemicellulose degradation was higher than that of other C-hydrolysis genes (Supplementary Figure S18). Our results also showed that those genes involved in C degradation and N cycling significantly differed between the two plantations (Supplementary Figure S19). Spearman analysis showed that there was no association between microbial richness and diversity and the microbial CNPS cycling genes, whereas fungal and protist β-diversity was significantly associated with microbial C degradation and C fixation. In contrast, bacterial β-diversity was significantly associated with microbial P cycling genes (Supplementary Figure S20). Interestingly, the protist RDA results of the microbial CNPS cycling genes indicated significantly condensed C degradation and C fixation on separate axes (*p* < 0.05) (Supplementary Figure S21).

**Figure 5:**
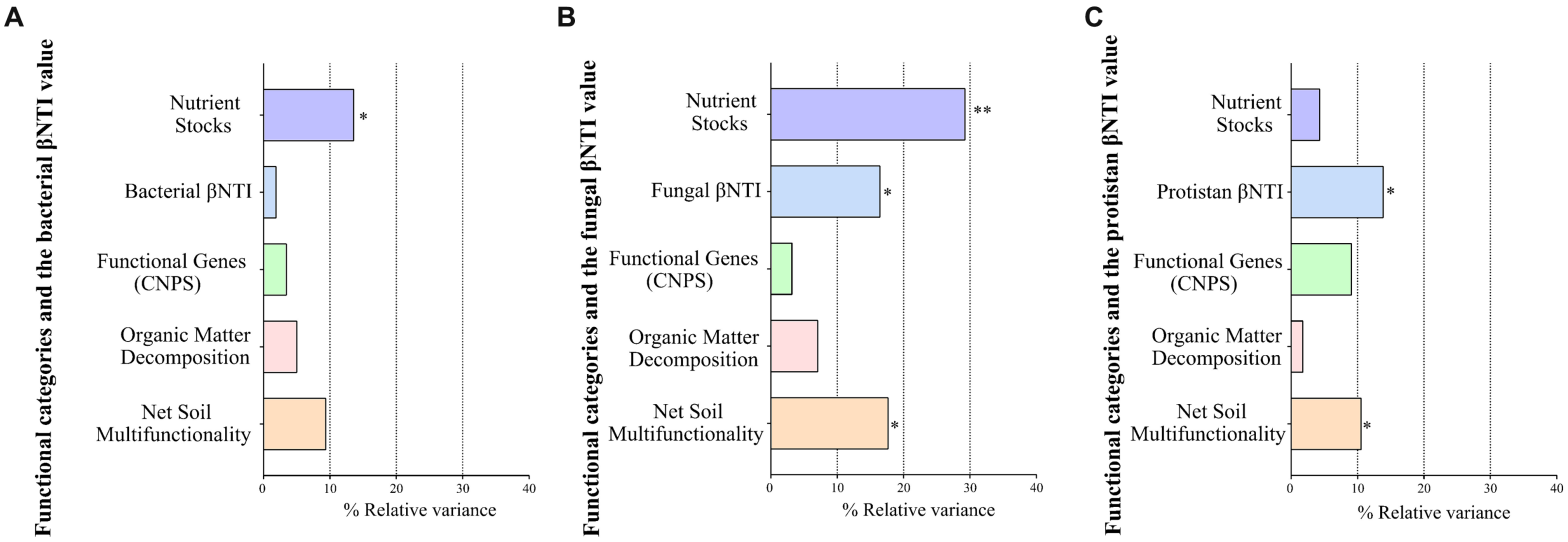
The impact of multifunctionality indices, functional genes, and microbial βNTI values on the bacterial **(A)**, fungal **(B)**, and protist **(C)** compositions using the PERMANOVA test (the Bray–Curtis distance matrix). Values less than 0.05 were considered significant.

Among the four functional categories (Supplementary Table S6), narrow functional categories (nutrient stocks, organic matter decomposition, and microbial functional genes) significantly differed between plantation types with seasonal variations (ANOVA, *p* < 0.05), whereas “net soil multifunctionality” did not differ significantly (*p* = 0.4306) (Supplementary Figure S22). Different levels of statistical associations were observed between multifunctionality indices and microbial compositions (Supplementary Figure S23) and microbial diversities (Supplementary Figure S24). The nutrient stocks and organic matter decomposition showed the highest number of significant associations with environmental variables (Supplementary Figure S25). Among the four functional groups, only the “nutrient stocks” function was associated with the minimum, average, and high air temperatures (Supplementary Figure S26). In particular, “nutrient stocks” were correlated with more features of the network than any of the other four functional categories (Supplementary Figure S27). The nutrient stocks explained the highest variations in the bacterial (Figure 5A) and fungal (Figure 5B) variations, whereas protist βNTI could explain the highest variation in protist communities (Figure 5C).

### 3.7 Assembly processes of microbial communities

**Figure 6:**
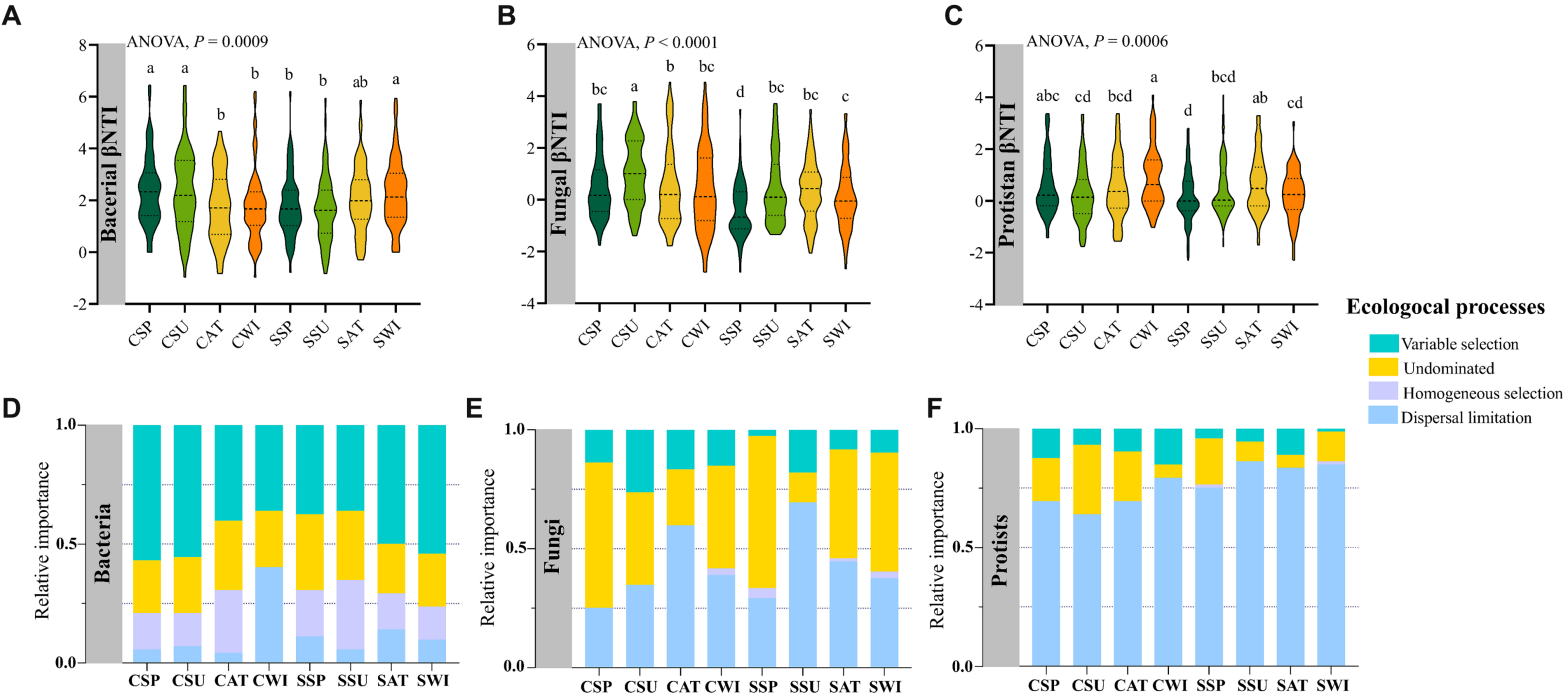
The microbial community assembly processes across two forest types seasonally. The values of the beta nearest taxon index (βNTI) for soil bacterial **(A)**, fungal **(B)**, and protistan **(C)** communities present. The upper and lower significance thresholds for the βNTI index were +2 and −2, respectively. The relative turnover in soil bacterial **(D)**, fungal **(E)**, and protistan **(F)** community assemblies is governed principally by deterministic processes (homogeneous and variable selections), stochastic processes (dispersal limitation and homogenizing dispersal), or undominated processes. Bars with different lowercase letters indicate significant differences within the microbial taxa phyla (*p* < 0.05) across two forest types with seasonal variations, as revealed by one-way ANOVA with Turkey’s *post hoc* test.

Results showed that the βNTI distributions differed significantly across plantation types, with seasonal variations for the bacterial (*F*_7,575_ = 3.5549, *p* = 0.0009), fungal (*F*_7,575_ = 6.9355, *p* < 0.0001), and protist communities (*F*_7,575_ = 3.6975, *p* = 0.0006) (Figures 6A–C). Null model analysis showed that the relative contributions of deterministic (|βNTI| ≥ 2) and stochastic (|βNTI| < 2) processes in various microbial compartments differed greatly. The deterministic processes of variable selection and homogenous selection were primarily responsible for the assembly and turnover of the soil bacterial communities (average: 62.49%) (Figure 6D), whereas the stochastic process of dispersal limitation was mainly responsible for the assembly and turnover of the fungal and protist communities (average: fungi: 42.36%, protists: 76.39%) (Figures 6E,F). Interestingly, among microbial compartments, the relative contribution of undominated processes to the fungal community (average 42.36%) was greater than that to the bacterial and protist communities (bacteria: 25.34%, protists: 14.93%, on average). The results showed that the bacterial deterministic process of homogenous selection shifted toward the dominance of stochastic processes (dispersal limitation) in the *Pinus* forest in winter (Figure 6D).

## 4 Discussion

### 4.1 Microbial diversities under seasonal variations and in different plantations

The seasonal dynamics of observed richness and Shannon diversity for each soil microbiome group exhibited distinct patterns (Figure 2 and Supplementary Table S13). These findings suggest that seasonal variations had an additional influence on soil microorganisms concerning different plant species, which is consistent with other studies (Žifčáková et al., 2016, 2017). As an example, unlike other studies where potential biotic and abiotic variables (Bates et al., 2013; Stefan et al., 2014), contributed to shaping the composition of protist structures, our PERMANOVA profiling revealed that the key factor driving the protist community structure was peroxidase (POD) activity (Supplementary Figure S12). PERMANOVA showed that the protist community, compared with the bacterial and fungal communities, was not influenced by either seasonal variation or plantation type, suggesting that protist taxa have a broad tolerance to the wide fluctuation of seasonal variabilities. Thus, results suggest that the protist composition is strongly but indirectly influenced by soil bacterial and fungal metabolic activities, particularly C-related functions. Suppose this pattern holds true across a range of ecosystem types. In that case, it implies that protists appear to be impacted to a unique degree compared with other soil biotas by alterations in soil oxidative activities and nitrogen fluctuations. Moreover, the GAM results indicated the importance of N cycles and oxidative enzymes in predicting the diversity and composition of the soil protist taxa (Supplementary Figure S10). This finding is partly consistent with previous findings, which highlighted that protists were the most susceptible soil biota to the application of nitrogen fertilizers (Zhao et al., 2019). Unlike the most recent study, in which biotic factors were identified as the major biogeographical predictor for protist consumers and mean annual temperature was the best predictor of the diversity and composition of phototrophs on a large scale in the Southern Hemisphere (Nguyen et al., 2021), our findings showed that seasonality and plantation type were important factors shaping protist trophic interactions at the local scale. The high abundance of consumers indicates the importance of protists’ role in controlling the frequency of other soil biotas (Oliverio et al., 2020).

PERMANOVA analysis revealed that seasonal variations are the best predictors of bacterial and fungal β-diversity community changes. Consistent with our findings, a previous study by Koranda et al. (2013) demonstrated that soil bacterial and fungal communities exhibited greater seasonal than spatial variation in alpine tundra soils. While seasonal variation was the best predictor for observed changes in both bacterial and fungal beta diversities, the factors explaining these variations differ. The number of soil variables significantly explaining fungal β-diversity is higher compared to those explaining bacterial β-diversity. This may be due to fungal lineages’ hyphal growth, allowing them to access more soil volume and obtain more substrates and nutrients than other organisms (Zheng et al., 2019). Thus, the potential alteration in edaphic parameters might substantially influence the fungal structure rather than bacterial taxa. Additionally, our results showed that, unlike the β-diversity of the fungal and protist taxa, soil moisture strongly and significantly explained bacterial β-diversity variation, which is consistent with a previous study that highlighted the importance of the soil water content for soil bacterial communities (Stres et al., 2008). However, PERMANOVA showed that the impact of different plantations significantly explained none of the microbial β-diversity variations. This finding is consistent with a previous report that the effect of soil type on shaping the bacterial rhizosphere was stronger than that of plant species (Liu et al., 2020). Our results showed that the relative abundance of bacterial taxa was disproportionately higher than other soil biota’s. More discussion can be found in the Supplementary material.

### 4.2 Soil microbial co-occurrence network complexity under seasonal successions and in different plantations

The segregated soil biota co-occurrence network patterns and their topological characteristics in each plantation plot showed pronounced seasonality (Figure 4 and Supplementary Table S20). Our results showed that the spring microbial network was complex compared to other seasons. The upward shift in network complexity from one season to another may have happened because of, or was induced by, several biotic or abiotic factors that generated an adaptive response in soil microbiomes seasonally. More specifically, many factors might contribute to this intricacy, such as controlling the temporary partitioning of nutrients between soil biota and plants due to the rapid alterations in microclimate from winter to spring, which consequently lead to transitions in some groups of soil microbiomes, abiotic stresses (wet-dry and freeze-thaw cycles), and the consumption of labile C composites, which drives the turnover of microbial community attendants to release labile N for plant uptake (Bardgett et al., 2005). Moreover, the increased network complexity in spring can indirectly be attributed to increased soil thermal variability and resource availability in spring, which fosters microbial diversity and network complexity (Zhu et al., 2021). Furthermore, the spring consisted of nodes with high BC values (Supplementary Table S21), indicating the importance of the control potential that an individual node exerts over the interactions of other nodes (Ma et al., 2016). These complex interactions in spring might consequently enhance system durability and resistance to adverse environmental conditions such as wet-dry and freeze–thaw cycles with changing seasons (Ji et al., 2021). Additionally, the soil microbiome networks could be used to visualize the scenarios in which the highest percentage of links among soil biota was observed between bacteria and protists (spring), indicating that bacteria and protist taxa were tightly linked within the microbiomes (Supplementary Table S20). It has also been shown that protist communities create a dynamic hub in soil biota (Zhao et al., 2019). More discussion on microbial co-occurrence network complexity can be found in the Supplementary material.

### 4.3 Diversity-function-assembly relationship with seasonality

Our results suggest that different afforested plantations changed ecosystem functioning seasonally, consistent with a previous finding (Broadbent et al., 2021). Results showed a positive relationship between microbial diversity and composition and multifunctionality in plantation forests seasonally, corroborating the positive biodiversity-ecosystem function relationships. These results align with recent studies on the northeastern and central Chinese Tibetan Plateau at the local scale (Jing et al., 2015) and the global scale (Delgado-Baquerizo et al., 2016). First, these outputs suggest that microbial diversity and composition have a leading role in maintaining ecosystem functioning (Zheng et al., 2019). Second, this association indicates high functional redundancy in soil biotas (Miki et al., 2014). A previous study highlighted that functional redundancy is a part of microbial communities (Xiang et al., 2020), and the functional redundancy of the microbial communities may explain why microbial taxonomic and functional gene diversity were correlated with different environmental variables in our study. Therefore, any changes in microbial diversity resulting from both soil biotic and abiotic factors might influence the soil’s multifunctionality, suggesting that the estimation of the causal association between microbial diversity and ecosystem functioning would be complex (Chen et al., 2020). Furthermore, our results suggested that broad functions, such as net soil multifunctionality, maybe more functionally redundant and thus better buffered against microbial shifts that are caused by seasonality in different plantation types or under other biotic and abiotic disturbances.

The null model supports the notion that distinct assembly processes drive the structure of different soil microbiomes. Deterministic processes, particularly variable selection, tended to be more critical in shaping the assembly of the soil bacterial communities. In contrast, stochastic processes dominated the soil fungal and protist community assemblies, with dispersal limitation playing a more critical role in both plantation types. A similar finding was recently reported for the assembly of the soil bacterial community (Liu et al., 2021). In accordance with the present results, a previous study demonstrated that stochastic processes are more important than deterministic processes for microbial community assembly at small scales (Zhou et al., 2022). Moreover, our results showed that seasonality played a decisive role in mediating the balance between stochastic and deterministic processes and showed a significant association with the diversity of soil microbiomes seasonally. The soil bacterial community assembly was governed by both deterministic and stochastic processes, with deterministic processes exerting a more substantial influence than stochastic processes. However, the relative importance of deterministic processes versus stochastic processes in bacterial community assembly varied between the different plantations, with seasonal variations in the *Pinus* forest. More specifically, seasonal transition in particular plantations markedly diminished the relative importance of homogenous selection and increased dispersal limitation of the bacterial community assembly in winter in the *Pinus* forest, corresponding to significantly lower bacterial Shannon diversity (Figure 2), higher bacterial gene copy numbers (Supplementary Figure S11), the association of the bacterial Shannon diversity with soil pH (Figure 3) and the bacterial βNTI value (Supplementary Figure S28), suggesting seasonality significantly increased the importance of stochastic processes, specifically in the bacterial community with stronger deterministic assembly. This result is likely related to the seasonal transition leading to selecting a particular group of soil microbiomes and influencing several soil edaphic conditions. Thus, selected microbes may have eminent potential to increase functions connected to nutrient stocks and organic matter decomposition and to decrease functional genes involved in CNPS cycling in the *Pinus* forest in winter (Supplementary Figure S20), which was associated with a shift in the deterministic process to the stochastic process for the bacterial community with the transition of season from autumn to winter for this plot (Figure 6D). In accordance with the present results, previous studies have demonstrated that determinism-dominated assembly processes generally selected limited taxa, which led to limited stress and perturbance tolerance (Vanwonterghem et al., 2014). Therefore, we propose that microbial communities such as bacteria, which were characterized by the highest gene copy numbers and the lowest Shannon diversity in winter, are potentially more susceptible to the assembly transition from deterministic to stochastic with seasonal variations. In contrast, microbial communities such as fungi and protists with stochastic assembly are potentially more resistant to the assembly transition in alkaline soils seasonally. Thus, the fluctuation of the microbial assembly is ultimately beneficial for ecosystem stability.

Studies have determined that environmental variables (such as air temperature, soil pH, and moisture) and habitat heterogeneity are vital determinants of community assembly (Liu et al., 2021; Zhou et al., 2021). Results highlighted that soil pH was positively correlated with the soil bacterial Shannon diversity. Therefore, the decrease in the soil bacterial Shannon diversity in winter (Figure 2) may be associated with the decrease in soil pH (Supplementary Table S13), suggesting that the fluctuation of soil pH in alkaline soil exerted substantial effects on the bacterial community assembly compared with the fungal and protist communities. We speculate that in alkaline soil with low habitat heterogeneity (monoculture plantation), the bacterial community assembly may be driven by deterministic processes with a high possibility of seasonal influence. By contrast, fungal and protist communities are more likely to be driven by stochastic processes, suggesting that stochastic processes may be more vital for soil microbial communities (Zhou et al., 2022).

## 5 Conclusion

The results predicted that protist community composition was uniquely structured with C-related functional activities (lignin-degrading enzymes, C-degradation, and C-fixation) relative to bacterial and fungal β-diversity variations, which were explained mainly by seasonal variations. Bacterial communities were deterministically (variable selection and homogenous selection) structured, whereas the stochastic process of dispersal limitation was mainly responsible for the assembly and turnover of the fungal and protist communities. Additionally, results showed that winter triggered an abrupt transition in bacterial community assembly from a deterministic to a stochastic process in the *Pinus* forest that was closely associated with a reduction in the bacterial Shannon diversity, with the pattern of a high level of nutrient cycling (nutrient stocks and organic matter decomposition functional categories), suggesting that the bacterial community with deterministic assembly is potentially more susceptible to the assembly transition with seasonal fluctuations in diversity and soil pH. This study contributes to local ecosystem prospects to model the behavior of soil biota seasonally and their implied effects on soil functioning and microbial assembly processes, which will benefit global-scale afforestation programs by promoting novel, precise, and rational urban plantation forests for future environmental sustainability and self-sufficiency.

## Data availability statement

The raw reads were deposited into the NCBI sequence read archive (SRA) database under BioProject IDs PRJNA688619, PRJNA688632, and PRJNA688645 for bacteria, fungi, and protists, respectively.

## Author contributions

MW: Conceptualization, Investigation, Writing – review & editing, Data curation, Formal analysis, Methodology, Resources, Software, Validation, Visualization, Writing – original draft. AM: Conceptualization, Data curation, Formal analysis, Investigation, Methodology, Resources, Software, Validation, Visualization, Writing – original draft, Writing – review & editing. CW: Conceptualization, Investigation, Validation, Writing – review & editing. LZ: Conceptualization, Investigation, Validation, Writing – review & editing. JY: Conceptualization, Investigation, Validation, Writing – review & editing. ZY: Conceptualization, Investigation, Writing – review & editing, Project administration, Supervision. JL: Conceptualization, Investigation, Writing – review & editing, Project administration, Supervision.

## Footnotes

^1^ http://www.xinhuanet.com/english/2021-04/01/c_139852880.htm

## Glossary

SWC: Soil water content
TC: total carbon
EC: soil electrical conductivity
SOM: soil organic matter
TK: total potassium
RAK: rapidly available potassium
SAK: slow-available potassium
TP: total phosphorus
TN: total nitrogen
HN: soil hydrolyzed nitrogen
IP: inorganic phosphorus
OP: organic phosphorus
AP: available phosphorus
TS: total sulfur
AS: available sulfur
BGC: β-Glucosidase
BXYS: β-xylosidase
BCL: β-cellobiosidase
LAP: Leucine Aminopeptidase
AGC: ɑ-Glucosidase
NAG: N-acetyl-β-D-glucosidase
ALP: Alkaline phosphatase
ASF: Arylsulfatase
PPO: Polyphenol oxidase
POD: Peroxidase
QIIME: quantitative insight into microbial ecology
OTU: operational taxonomic units
RDP: ribosomal database project
PR2: protist ribosomal reference
BC: betweenness centrality
NMDS: non-metric multidimensional scaling analysis
ANOSIM: analysis of similarity PERMANOVA permutational multivariate analysis of variance
RDA: redundancy analysis
GAM: generalized additive models
EEA: ecoenzymatic activity
EES: ecoenzymatic stoichiometry
LDA & LEfSe: linear discriminant analysis (LDA) effect size (LEfSe)
ND: network nodes
NG: network edges
AD: average degree
APL: average path length
ML: modularity
ACC: average clustering coefficient
