# Supplementary material for: Seasonal variations affect the ecosystem functioning and microbial assembly processes in plantation forest soils
Source: Front Microbiol. 2024 Jul 26;15:1391193. doi: 10.3389/fmicb.2024.1391193 (PMC11310165; doi:10.3389/fmicb.2024.1391193)
Supplement: Supplementary file 1 [file Presentation_1.pdf]

Supplementary Figures for:

Seasonal variations affect the ecosystem functioning and microbial assembly processes in plantation forests

Min Wang[1]†, Abolfazl Masoudi[2]†, Can Wang[1], Liqiang Zhao[1], Jia Yang[1], Zhijun Yu[1]*, Jingze Liu[1]*

[1]Hebei Key Laboratory of Animal Physiology, Biochemistry, and Molecular Biology, Hebei Collaborative Innovation Center for Eco-Environment, Hebei Research Center of the Basic Discipline of Cellular Biology, Ministry of Education Key Laboratory of Molecular and Cellular Biology, College of Life Sciences, Hebei Normal University, Shijiazhuang, Hebei 050024, P. R. China

[2]Department of Biological Sciences, University of Illinois, Chicago, Illinois, United States of America

Address: College of Life Sciences, Hebei Normal University, 20 Nanerhuan East Road, Shijiazhuang, Hebei Province, China

Email addresses:

*Corresponding Authors: Tel/fax: +86 311 80787519, yuzhijun@hebtu.edu.cn (ZY: https://orcid.org/0000-0003-0122-7525), liujingze@hebtu.edu.cn (JL: https://orcid.org/0000-0002-0923-1775).

† These authors contributed equally to this study.

Supplementary Figures

Supplementary Figures:

Fig. S1:
The map shows the sampling locations.

Fig. S2:

Associations (Spearman's *P*) between the relative abundances of edaphic parameters and ecoenzymatic activities (EEAs) and stoichiometry (EES).

Fig. S3:

Relationship between ecoenzymatic activities (EEAs) and stoichiometry (EES) with soil properties evaluated using a generalized additive model.

Fig. S4:

Venn diagram presenting the number of share and unique OTUs in different plantation types with four distinct seasons. (A) bacterial Venn, (B) fungal Venn, and (C) protistan Venn.

Fig. S5:

Detection of the top 10 dominant microbial phyla and classes by 16S rRNA (bacteria A, B), ITS1 (fungi C, D), and 18S rRNA (protists E, F). Abbreviations: C (*P. tabulaeformis*), S (*S. japonica*), SP (spring), SU (summer), AT (autumn), and WI (winter).

Fig. S6:

Microbial α-diversity indices for bacterial lineages (A, B, C, and D), fungal lineages (E, F, G, and H), and protistan lineages (I, J, K, and L).

Fig. S7:

Non-metric multidimensional scaling (NMDS) plot for different seasons and plant species.

Fig. S8:

Differentially abundant microbial groups (based on LEfSe) were observed in response to different plantation types and distinct seasons with relative abundances of greater than 1% of the entire communities.

Fig. S9:

Comparison of soil microbial community clustering from different plantation types and seasons using hierarchical clustering analysis (UPGMA using the Bray-Curtis dissimilarities). (A) bacterial, (B) fungal, and (C) protists hierarchical clustering.

Fig. S10:

Supplementary Figures

The generalized additive model finds a significant association between microbial richness (Chao1), and microbial diversity (Shannon) with edaphic parameters and enzyme activities.

Fig. S11:

The abundance of soil biota in different plantation types and distinct seasons using real-time quantitative PCR.

Fig. S12:

The impact of environmental variables on bacterial (A), fungal (B), and protist community compositions using the PERMANOVA test (the Bray-Curtis distance matrix).

Fig. S13:

Life history strategy (photophagotrophs, parasites, consumers, and phototroph) of protistan lineages for each plantation type and distinct season.

Fig. S14:

The linkage between co-occurrence network parameters and environmental variables. Different network parameters were linked with different environmental variables, among which parameters associated with temperature appeared to be the strongest.

Fig. S15:

Principal Component Analysis (PCA) profile based on the Bray-Curtis distances showing the distinct but concentric patterns of CNPS cycling genes in different plantation types with seasonal variations.

Fig. S16:

The abundance of key-stone C-hydrolysis genes. The potentials for starch degradation, *amyA*, and *apu* were significant, but the absolute abundance of *sga* was not significant in soils (P = 0.2041).

Fig. S17:

The abundance of CNPS cycling genes across two different plantation types (ANOVA, HSD-Tukey Kramer test).

Fig. S18:

Spearman correlation heatmap between microbial diversity indices and CNPS cycle genes.

Fig. S19:

The link between CNSP cycling genes with bacterial (A), fungal (B), and protistan (C) community compositions.

Supplementary Figures

Fig. S20:

Average multifunctionality indices response to different plantations with seasonal variations.

Fig. S21:

The correlation ship between multifunctionality indices and bacterial (A), fungal (B), and protistan (C) compositions.

Fig. S22:

Microbial diversity (richness and Shannon diversity)-multifunctionality relationship using the GAM analysis.

Fig. 23:

Heatmap of environmental driving factors derived multifunctionality indices.

Fig. S24:

The association of multifunctionality categories with annual air temperature.

Fig. S25:

The linkage between co-occurrence network parameters and functional groups such as nutrient stocks, organic matter decomposition, functional genes (CNPS), and net soil multifunctionality.

Fig. S26:

The linkage between microbial βNTI values with environmental variables (A), microbial diversity indices (α- and β-diversities) (B), and co-occurrence network parameters (C).

Supplementary Figures

Supplementary Tables:

Table S1: Information of seasonal temperature and precipitation

Table S2: Procedure for evaluation of soil elemental parameters

Table S3: Extracellular enzyme abbreviations

Table S4: The qPCR primers and PCR cycle conditions in this study

Table S5: Information of 72 CNPS cycling genes detected in gene chip

Table S6: Ecological attributes within each soil functional group

Table S7: The impact of plantation types with seasonal variations on soil properties and enzyme activities in the XNA

Table S8: The C:N ratio for different seasons

Table S9: The C:P ratio for different seasons

Table S10: The N:P ratio for different seasons

Table S11: The C:P:N ratio for different seasons

Table S12: The relative abundance of microbial phyla and classes in different plantation types and different seasons

Table S13: α-diversity indices of soil microbiomes in different plantations with seasonal variations

Table S14: Bacterial ANOSIM analysis

Table S15: Fungal ANOSIM analysis

Table S16: Protistan ANOSIM analysis

Table S17: Bacterial PERMANOVE analysis based on environmental factors

Table S18: Fungal PERMANOVE analysis based on environmental factors

Table S19: The Protistan PERMANOVE analysis based on environmental factors

Supplementary Figures

Table S20: Topological indices of each network in Fig. 6

Table S21: Top 10 nodes with the highest betweenness centrality (BC) value for different plantation types seasonally

Table S22: Network structure for soil microbial interaction at different plantation types seasonally

Supplementary Figures

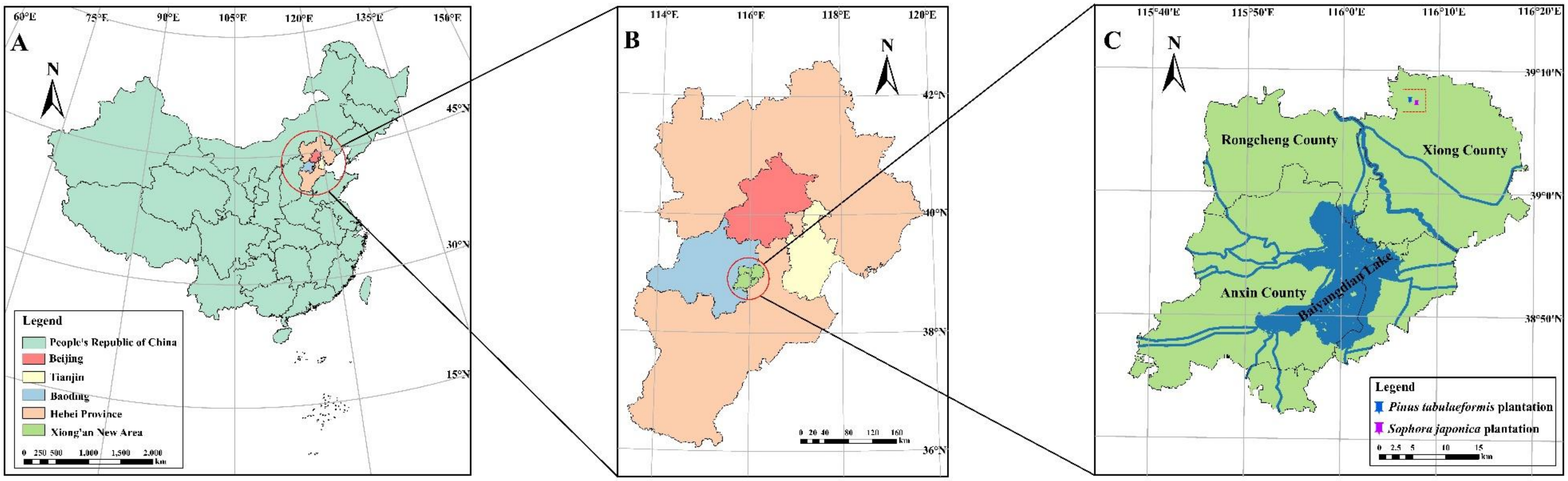

Fig. S1:

The map shows Beijing-Tianjin-Hebei, a regional city cluster called 'Jing-Jin-Ji' (A), the Xiong'an New Area (B) location, and the experimental plots. Geographical map with coordinates created by ArcGIS for Windows ver. 10.2 (Environmental Systems Research Institute [ESRI; www.esri.com; 2012], Redlands, USA).

Supplementary Figures

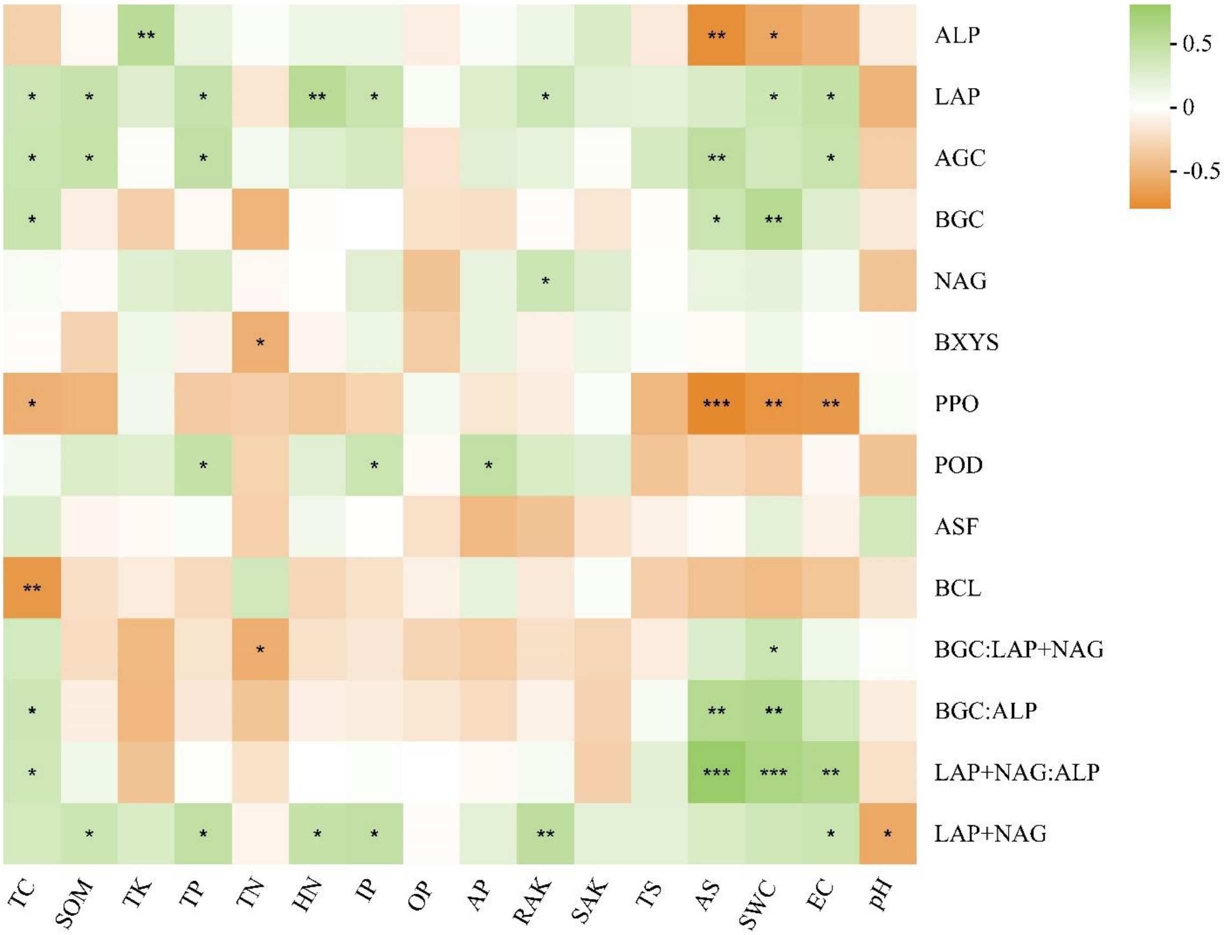

Fig. S2:

Associations (Spearman's *P*) between the relative abundances of edaphic parameters and ecoenzymatic activities (EEAs) and stoichiometry (EES). The correlation coefficients ranging from negative to positive are indicated by color intensity changing from light orange to light green, as shown by the figure scale. * $P < 0.05$, **$P < 0.01$, ***$P < 0.001$. The results showed a negative association of soil pH with LAP + NAG. In addition, the result could clearly show the significant and positive association between soil moisture and C:P, N:P, and C: N ratios ($P < 0.05$).

Supplementary Figures

Abbreviations:

SWC, Soil water content; TC, total carbon; EC, soil electrical conductivity, SOM, soil organic matter, TK, total potassium, RAK, rapidly available potassium, SAK, slow-available potassium, TP, total phosphorus, TN, total nitrogen, HN, soil hydrolyzed nitrogen, IP, inorganic phosphorus; OP, organic phosphorus, AP, available phosphorus, TS, total sulfur; AS, available sulfur; BGC, β-Glucosidase; BXYS, β- xylosidase; BCL, β-cellobiosidase; LAP, Leucine Aminopeptidase; AGC, α-Glucosidase; NAG, N-acetyl-β-D-glucosidase; ALP, Alkaline phosphatase; ASF, Arylsulfatase; PPO, Polyphenol oxidase; POD, Peroxidase; EEA, ecoenzymatic activity; EES: ecoenzymatic stoichiometry

Supplementary Figures

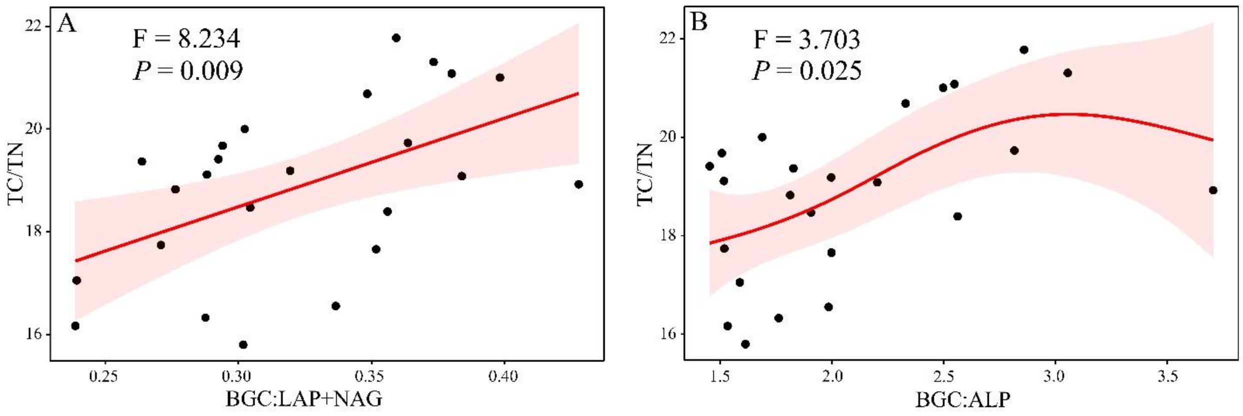

Fig. S3:

Relationship between ecoenzymatic activities (EEAs) and stoichiometry (EES) with soil properties evaluated using a generalized additive model. Among all, the association of TC/TN with BGC: LAP+NAG (C: N) (A) and BGC: ALP (C:P) (B) was statistically significant, which is presented. Non-significant associations were not included in this picture.

Supplementary Figures

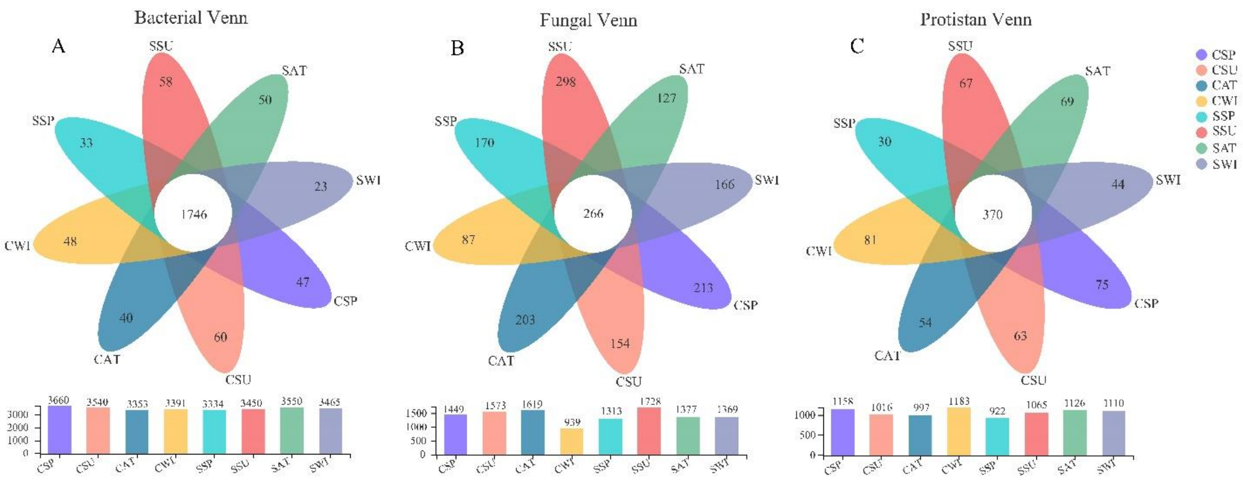

Fig. S4:

Venn diagram presenting the number of shared and unique OTUs in different plantation types with four distinct seasons. (A) bacterial Venn, (B) fungal Venn, and (C) protistan Venn. The highest number of unique bacterial and fungal OTUs was detected at the Chinese scholar tree in summer (SSU). In contrast, the highest number of unique protistan OTUs was detected at the Chinese pine tree plot in winter (CWI). Interestingly, the lowest number of bacterial, fungal, and protistan OTUs were observed in winter, and the Chinese pine plot was composed of the lowest fungal OTUs. In contrast, the Chinese scholar tree plot contained the lowest bacterial and protistan OTUs. The fungal Venn diagram revealed that SSU samples yielded the maximum number of unique OTUs (298 OTUs), whereas the minimum number of unique OTUs (87 OTUs) was from CWI samples. The core fungal community was 266 OTUs in all soil samples. The number of OTU appeared to be the minimum value in CWI samples, but the maximum value was found in SSU samples. These results might indicate that plantation types are an essential factor in shaping microbial lineages in soils with seasonal transitions. Abbreviations: C (Chinese pine tree plot), S (Chinese scholar tree plot), SP (spring), SU (summer), AT (autumn), and WI (winter).

Supplementary Figures

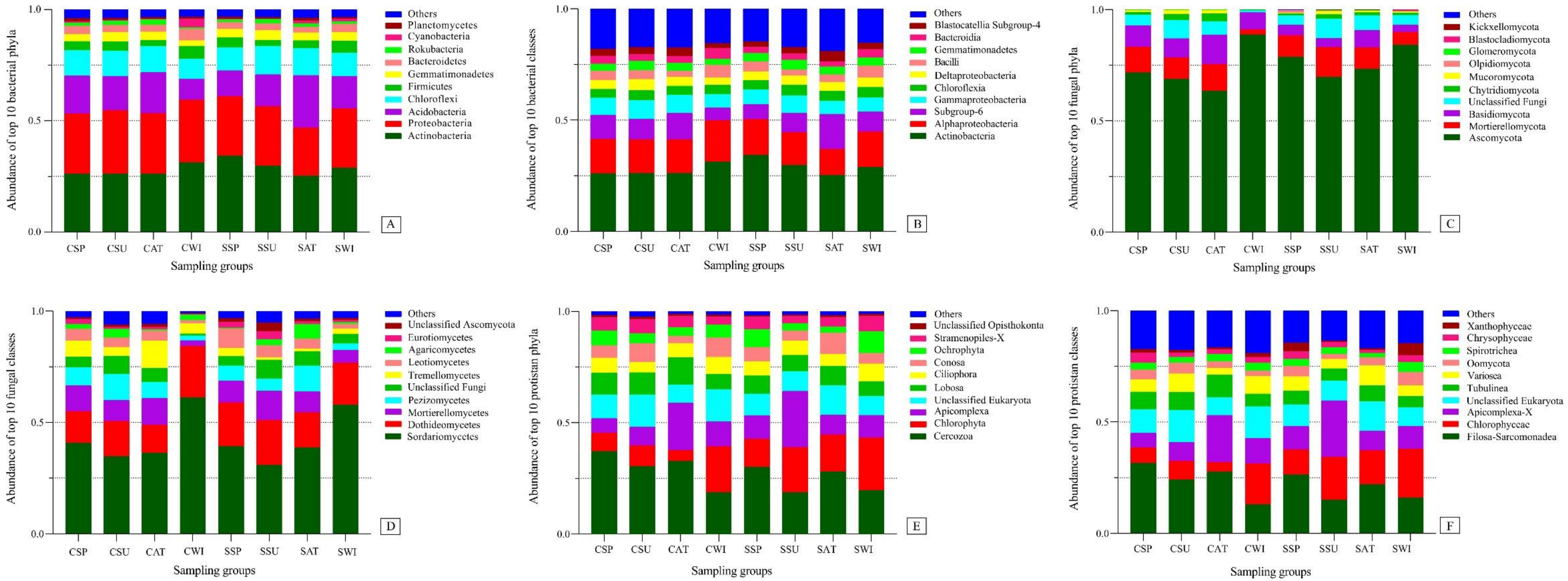

Fig. S5:

Detection of the top 10 dominant microbial phyla and classes by 16S rRNA (bacteria A, B), ITS1 (fungi C, D), and 18S rRNA (protists E, F). Abbreviations: C (*P. tabulaeformis*), S (*S. japonica*), SP (spring), SU (summer), AT (autumn), and WI (winter).

The abundance of ectomycorrhizal (ECM) fungi (and thus the Basidiomycota) showed seasonal variations, in which its abundance was dropped in winter for both plant species. We realized that the relative abundance of ectomycorrhizal (ECM) fungi was higher

than mycorrhizal (AM) fungi in both experimental plots. Interestingly, the relative abundance of both fungal groups was more elevated in autumn. Surprisingly, the difference in abundance of ECM fungi decreased for both plant species from spring to summer (CP: 12.33%, CS: 15.95%).

Abbreviations: C (Chinese pine tree plot), S (Chinese scholar tree plot), SP (spring), SU (summer), AT (autumn), and WI (winter).

Supplementary Figures

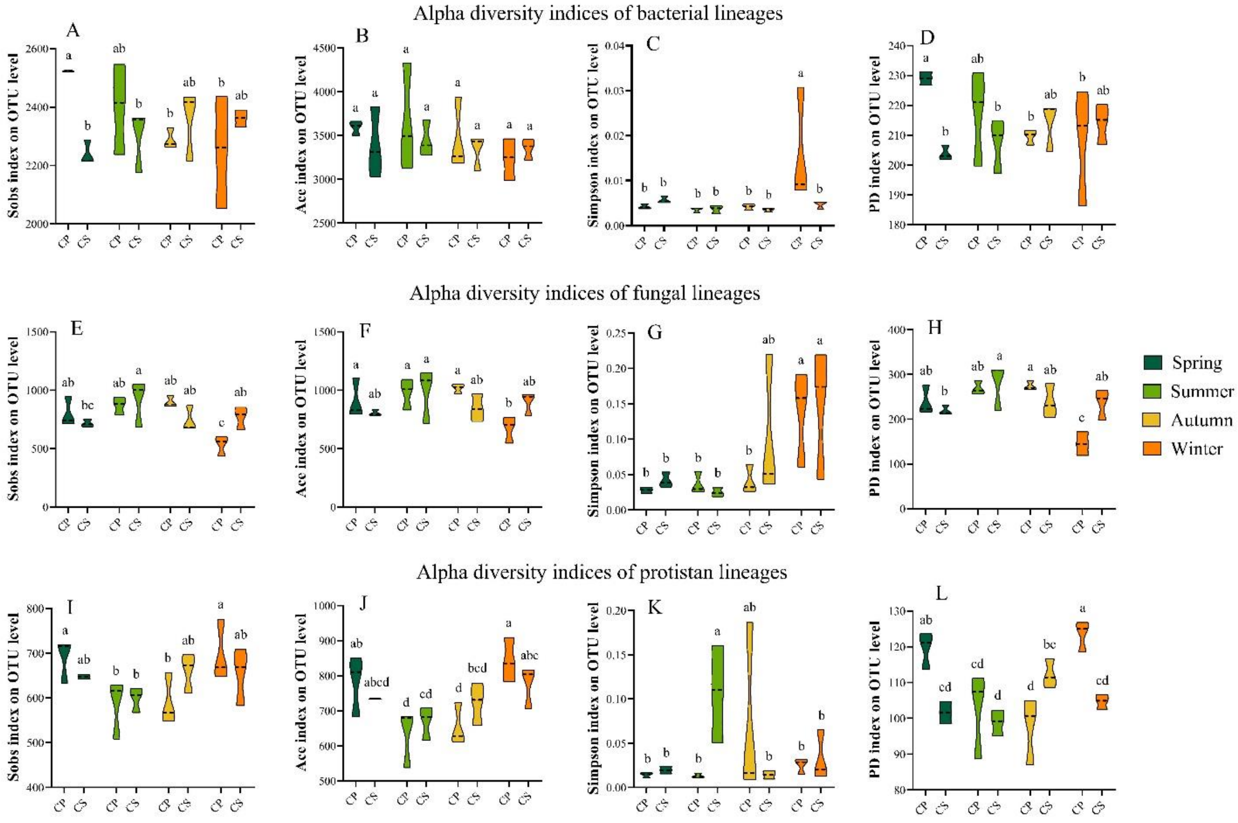

Fig. S6:

Microbial α-diversity indices for bacterial lineages (A, B, C, and D), fungal lineages (E, F, G, and H), and protistan lineages (I, J, K, and L).

Abbreviations: Sob (observed species), ACE (abundance-based coverage estimator, non-parametric method based on abundance), Simpson index and phylogenetic diversity index (PD), C (Chinese pine tree plot), S (Chinese scholar tree plot), SP (spring), SU (summer), AT (autumn), and WI (winter).

Supplementary Figures

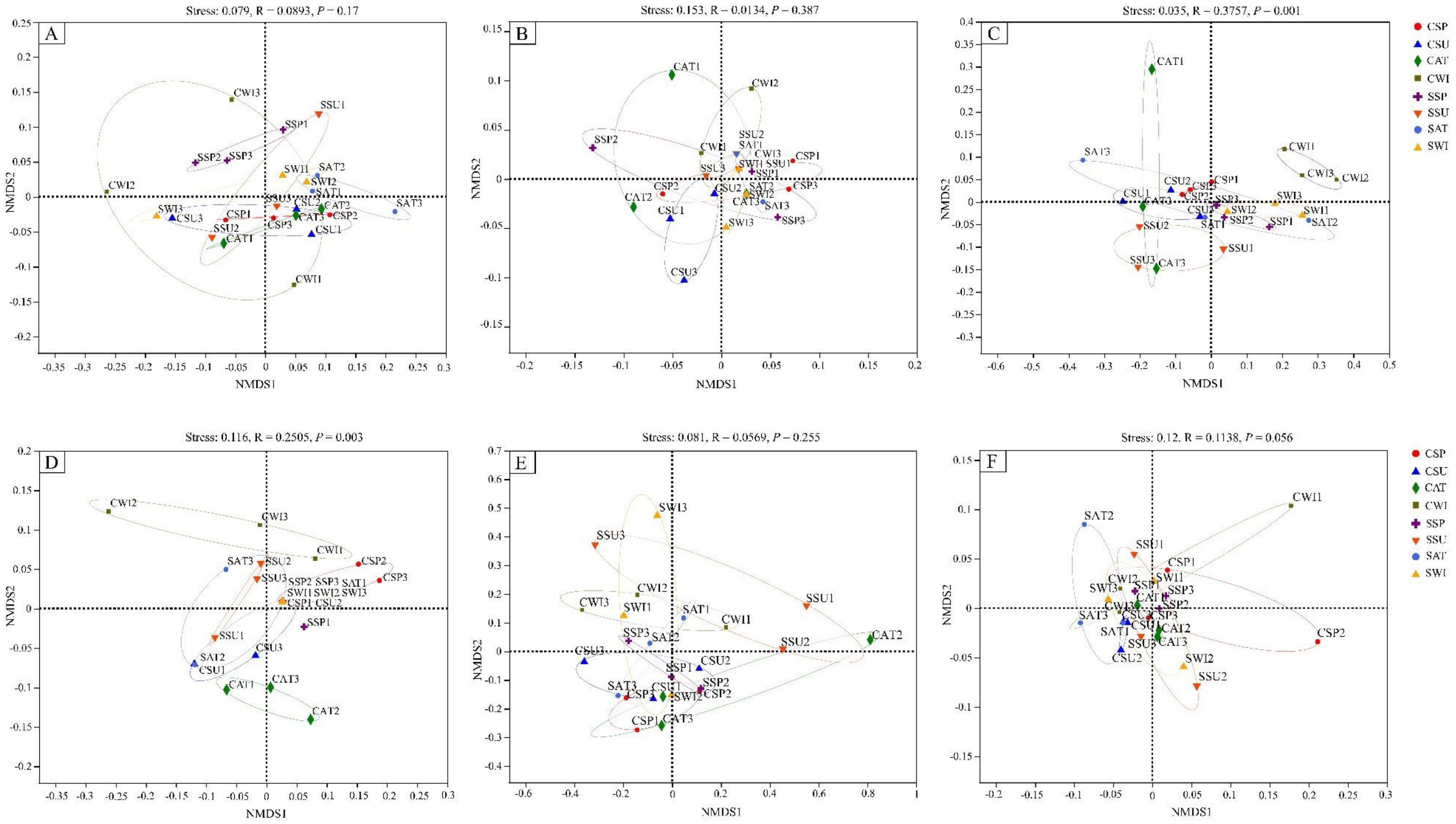

Supplementary Figures

Fig. S7:

Non-metric multidimensional scaling (NMDS) plot for different seasons and plant species. (A) bacterial NMDS plot based on the weighted UniFrac, (B) bacterial NMDS plot based on the unweighted UniFrac, (C) fungal NMDS plot based on the weighted UniFrac, (D) fungal NMDS plot based on the unweighted UniFrac, (E) protists NMDS plot based on the weighted UniFrac, and (F) protists NMDS plot based on the unweighted UniFrac. The samples were clustered by seasons and plant species and represented with different colors. The ellipses represent the centroid's standard error for each group of soil samples with a confidence limit of 95%.

Abbreviations: C (Chinese pine tree plot), S (Chinese scholar tree plot), SP (spring), SU (summer), AT (autumn), and WI (winter).

Supplementary Figures

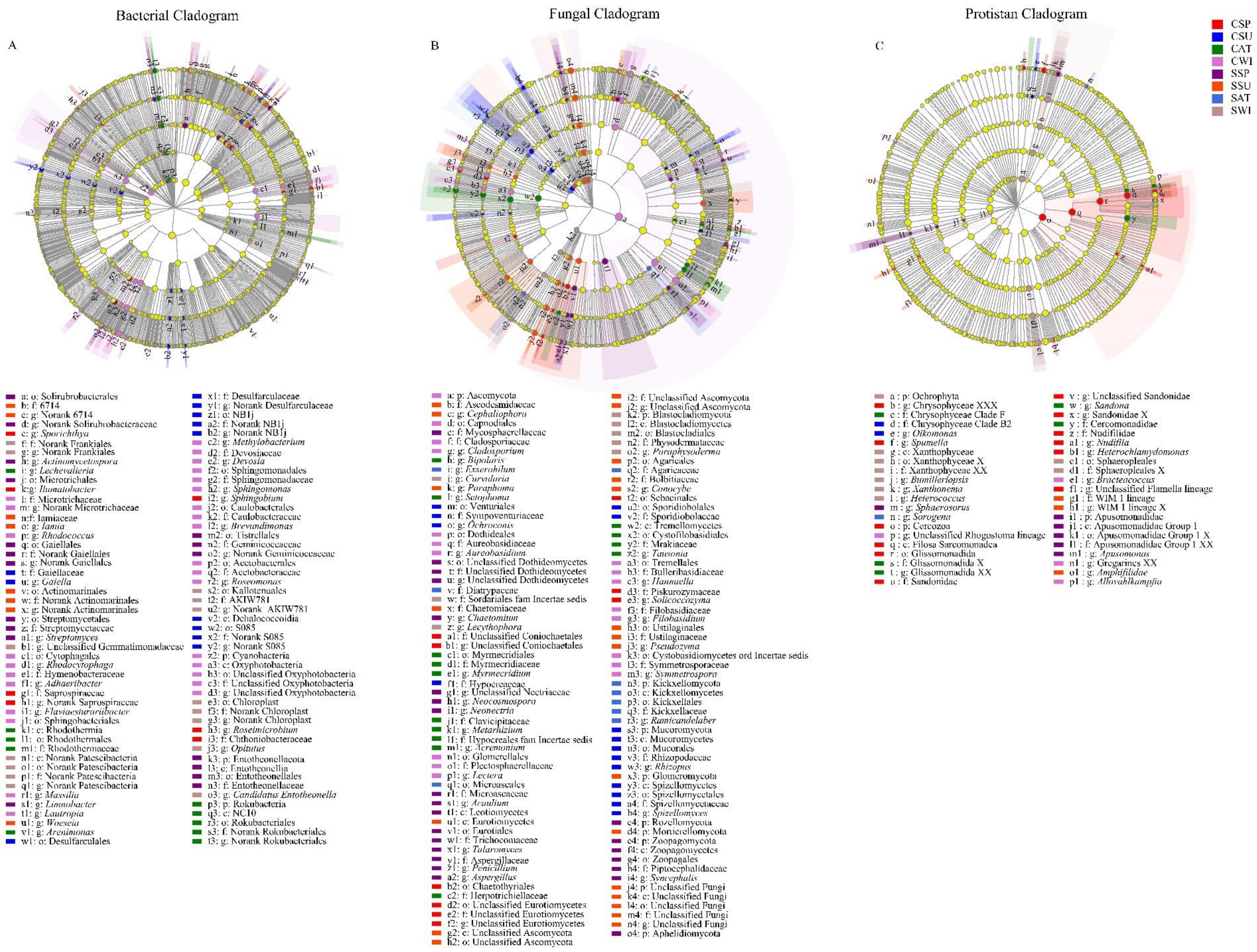

Supplementary Figures

Fig. S8:

Differentially abundant microbial groups (based on LEfSe) were observed in response to different plantation types and distinct seasons with relative abundances of greater than 1% of the entire communities. The cladogram plotted from LEfSe analysis shows the taxonomic levels represented by rings with phyla in the innermost ring and genera in the outermost ring. Each circle is a member within that level. Those taxa in each level are colored by different plantation types and various seasons for which it is more abundant ($P < 0.05$; LDA score 4.0). The cladogram showed interesting results. For instance, considering the CP plot, the abundance of the Mucoromycotina fungal symbiosis phylum was high in summer, which recently highlighted that this group forms an intimate symbiosis with liverworts as well (Rimington et al., 2019). Considering insect-associated fungi, *Metarhizum*, which is newly recognized as a plant symbiont with the capability of transferring insect nitrogen to plants (Behie et al., 2017), was detected as a biomarker in autumn at the CP plot. Considering the CS plot, Glomeromycota (most arbuscular fungi are categorized in this phylum) was recognized as a biomarker in the summer. Considering bacterial taxa in the CP plot, the highest number of biomarkers was recorded in winter, in which the vital group of Cyanobacteria was included.

Abbreviations: C (Chinese pine tree plot), S (Chinese scholar tree plot), SP (spring), SU (summer), AT (autumn), and WI (winter).

Supplementary Figures

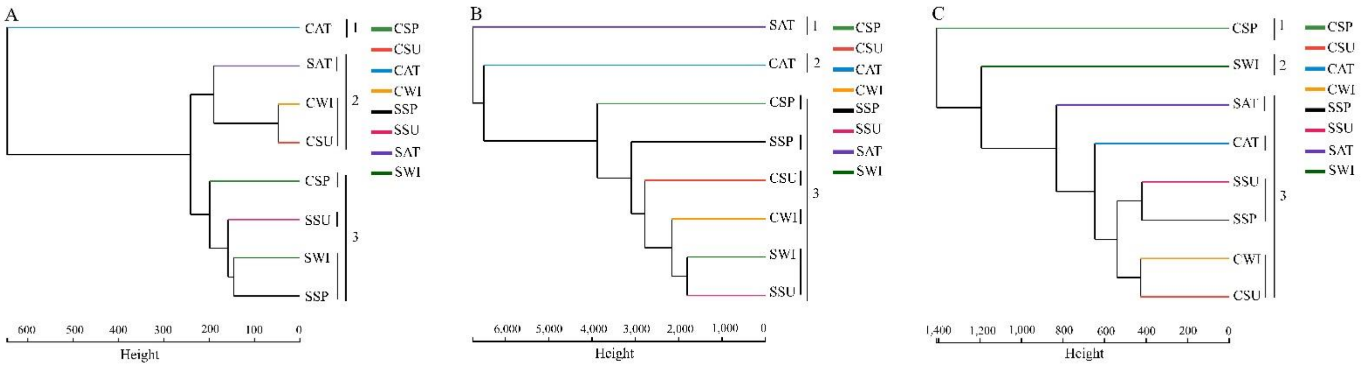

Fig. S9:

Comparison of soil microbial community clustering from different plantation types and seasons using hierarchical clustering analysis (UPGMA using the Bray-Curtis dissimilarities). (A) bacterial, (B) fungal, and (C) protists hierarchical clustering.

Abbreviations: C (Chinese pine tree plot), S (Chinese scholar tree plot), SP (spring), SU (summer), AT (autumn), and WI (winter).

Supplementary Figures

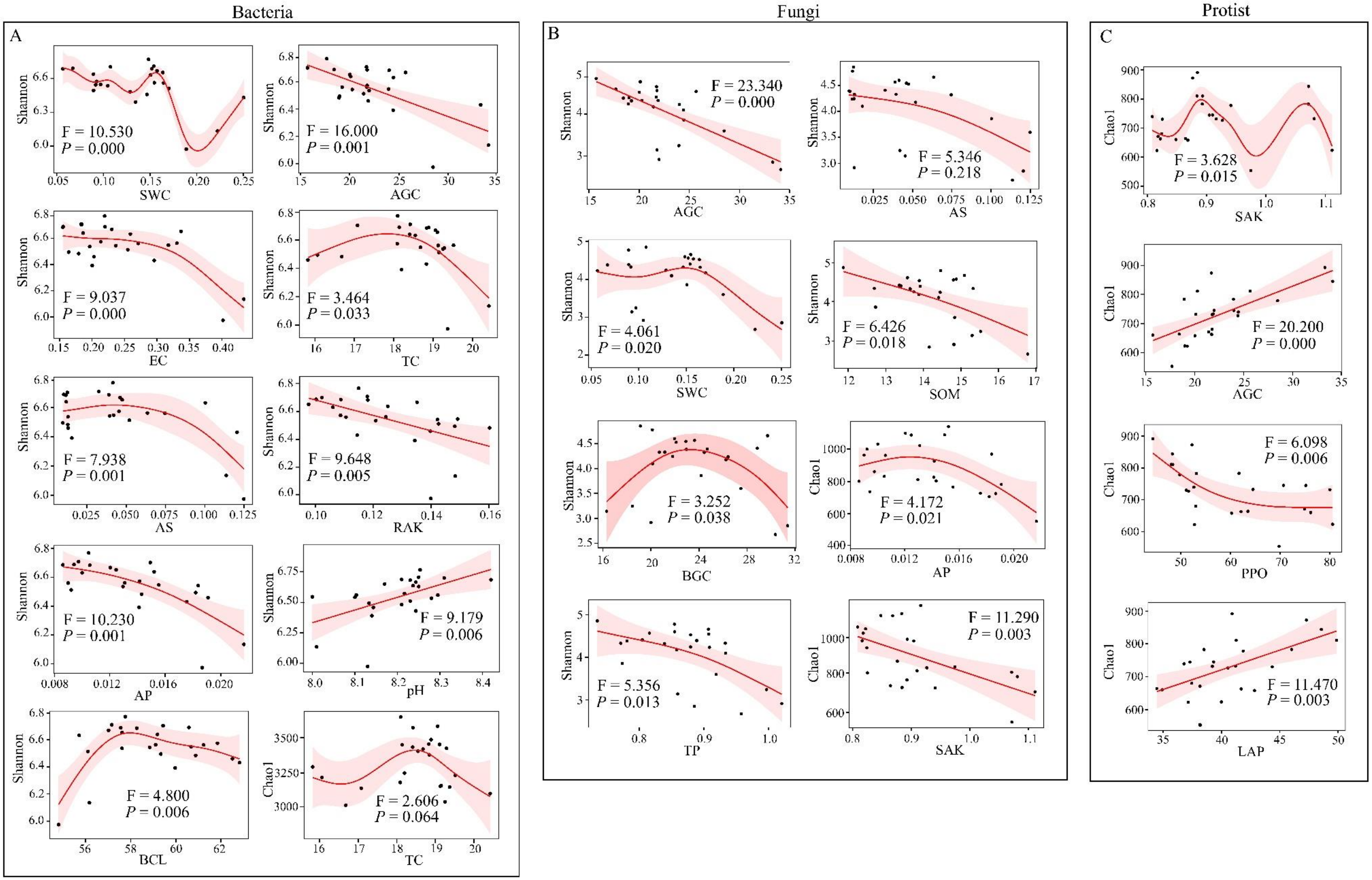

Supplementary Figures

Fig. S10:

The generalized additive model finds a significant association between microbial richness (Chao1) and microbial diversity (Shannon) with edaphic parameters and enzyme activities. (A) All the possible and significant associations of bacterial richness and diversity with edaphic parameters and enzyme activities. (B) All the potential and significant association of fungal richness and diversity with edaphic parameters and enzyme activities. (C) All the possible and significant associations of protists richness and diversity with edaphic parameters and enzyme activities. The results showed that the highest bacterial Shannon diversity was observed at the lower concentration of soil parameters like SWC, AGC, EC, AS, RAK, AP, and BCL. The highest bacterial Shannon diversity peaked at alkaline (pH = 8.4). We realized that TC had the same impact on the bacterial Shannon diversity and Chao1 richness, which peaked in the middle concentration of TC. The highest fungal Shannon diversity was recorded at the lower concentration of AGC, AS, SOM, and TP, whereas this index peaked at the middle value of soil moisture (SWC). The fungal richness peaked at the lower concentration of AP and SAK. None of the soil factors significantly affected the protists' Shannon diversity, whereas protists' richness showed opposite trends. The richness of Protists peaks in high concentrations of LAP and AGC (N-cycling enzymes), whereas its richness peaks in the lower concentration of the PPO enzyme. Moreover, GAM results indicated the positive association of protists' richness with hydrolase enzymes like those that were related to N-cycling enzymes (LAP and ALP enzymes). In contrast, high protists richness was observed at a low concentration of polyphenol oxidase (PPO). PPO is an effective enzyme to degrade lignin as a significant component of SOM (Sinsabaugh, 2010). Thus, it seems that the high richness of protists can be observed at the high level of SOM. Our results verified the spatiotemporal variation of soil phenol oxidase and peroxidase activities. Some studies showed that the activity of these two enzymes either becomes undetectable or

dipped in summer (Criquet et al., 2000; Sinsabaugh et al., 2005; Finzi et al., 2006; Weintraub et al., 2007), probably as a result of changes in carbon availability (Sinsabaugh, 2010) or moisture limitation (Toberman et al., 2008). Another group of studies found no significant seasonal variation (Sinsabaugh et al., 2003; Boerner et al., 2005; Kellner et al., 2009). Decker et al. (1999) showed that hydrolase activities generally correlated with soil organic matter (SOM) and nutrient availabilities. In contrast, phenol oxidase activity associated with soil pH and was more challenging to model. As an interesting point of view, unlike bacterial and fungal richness, two hydrolytic enzymes of N-cycling enzymes (AGC and LAP) were positively correlated with four protist alpha diversity indices (Spearman correlation, $P < 0.05$) (Fig. S2). In line with this result, there was a negative association between bacterial (Fig. S10A) and fungal (Fig. S10B) Shannon diversities with the AGC concentration. In contrast, a positive association between the AGC concentration and protists richness was observed (Fig. S10C). Also, the GAM analysis showed the high protists richness in the low concentration of PPO, and the positive association between protists richness and the LAP concentration was recorded (Fig. S10C).

Supplementary Figures

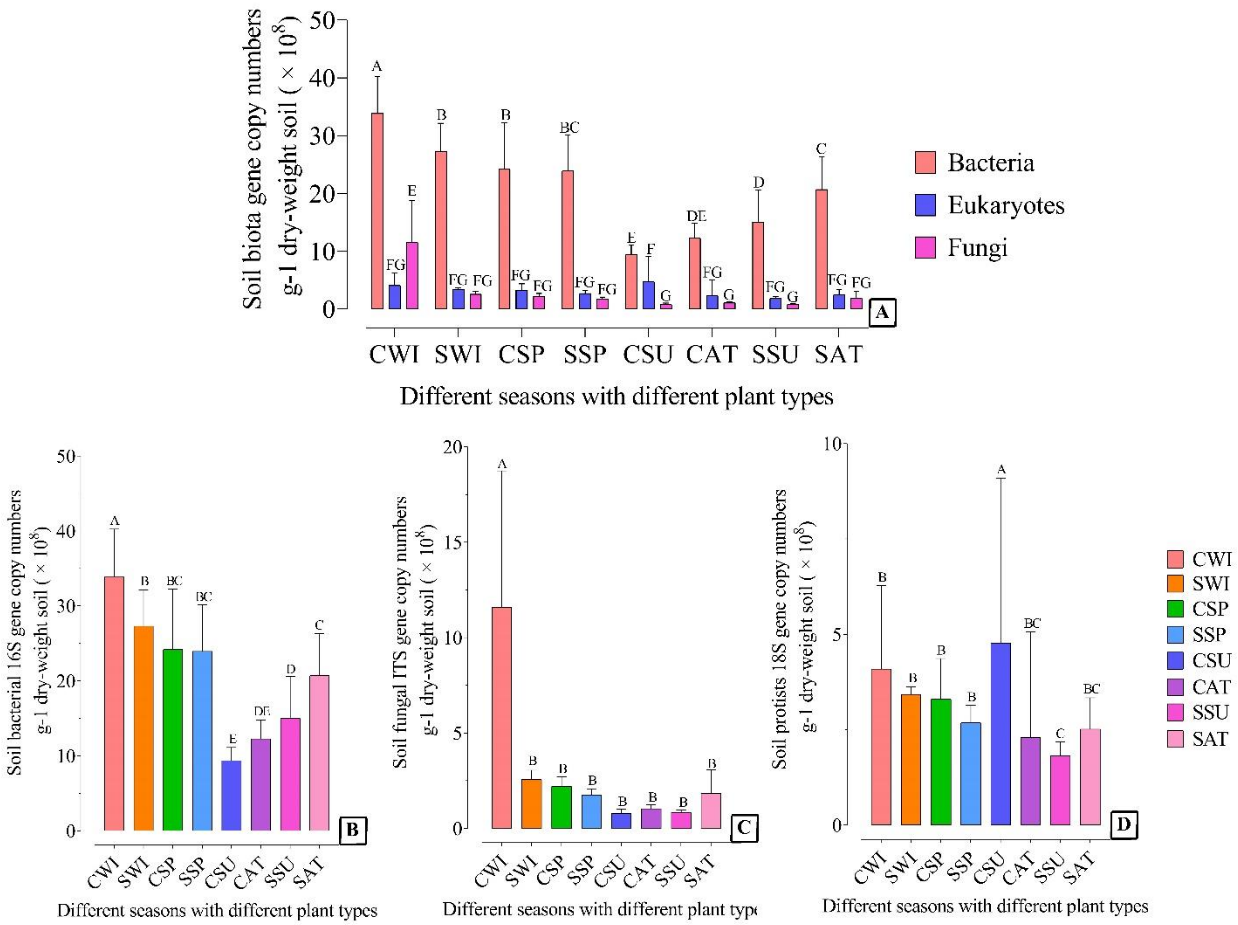

Fig. S11:

The abundance of soil biota in different plantation types and distinct seasons using real-time quantitative PCR. Interestingly, the highest gene copy numbers of bacteria were observed in winter and spring, whereas the bacterial gene copy numbers were significantly dropped in summer and autumn (Fig. S11B). No significant difference was observed for fungal gene copy numbers for all samples except CWI samples (Fig. S11C). The gene copy number of soil protists was higher for CWI samples, whereas the gene copy number of soil protists was higher for CSU samples (Fig. S11D). Briefly, all reactions were carried out in three technical replicates for each soil replicate (In total, nine replicates for three soil samples of each experimental plot) to ensure accuracy. The

reactions were prepared in a final volume of 20 μL containing one μL of template DNA, 0.8 μL of each primer (5 μM), 7.4 μL ddH_2O, and ten μL of 2x Taq™ Plus Master Mix (Vazyme Biotech Co., Ltd, Nanjing, China). Standard curves were constructed with a 10-fold serial dilution of perceived copy numbers of plasmids (pMD18-T, 2692 bp) containing 16S rRNA, ITS1, and 18S genes fragments, and they were linear from 7.66×10^2 to $7.66\ 10^8$ copies/μL (R^2 >0.99, E = 106.98%), 4.84×10^3 to 4.84×10^7 copies/μL ($R^2 > 0.98$, E = 98.44%), 5.60×10^2 to 5.60×10^5 copies/μL ($R^2 > 0.99$, E = 109.93%), respectively. The bacterial biomass ranged on average from 8.65×10^8 to 34.84×10^8 copies/g dry soil, the fungal biomass ranged on average from 0.68×10^8 to 6.84×10^8 copies/g dry soil, and the protistan biomass ranged on average from 1.17×10^8 to 7.22×10^8 copies/g dry soil.

Abbreviations: C (Chinese pine tree plot), S (Chinese scholar tree plot), SP (spring), SU (summer), AT (autumn), and WI (winter).

Supplementary Figures

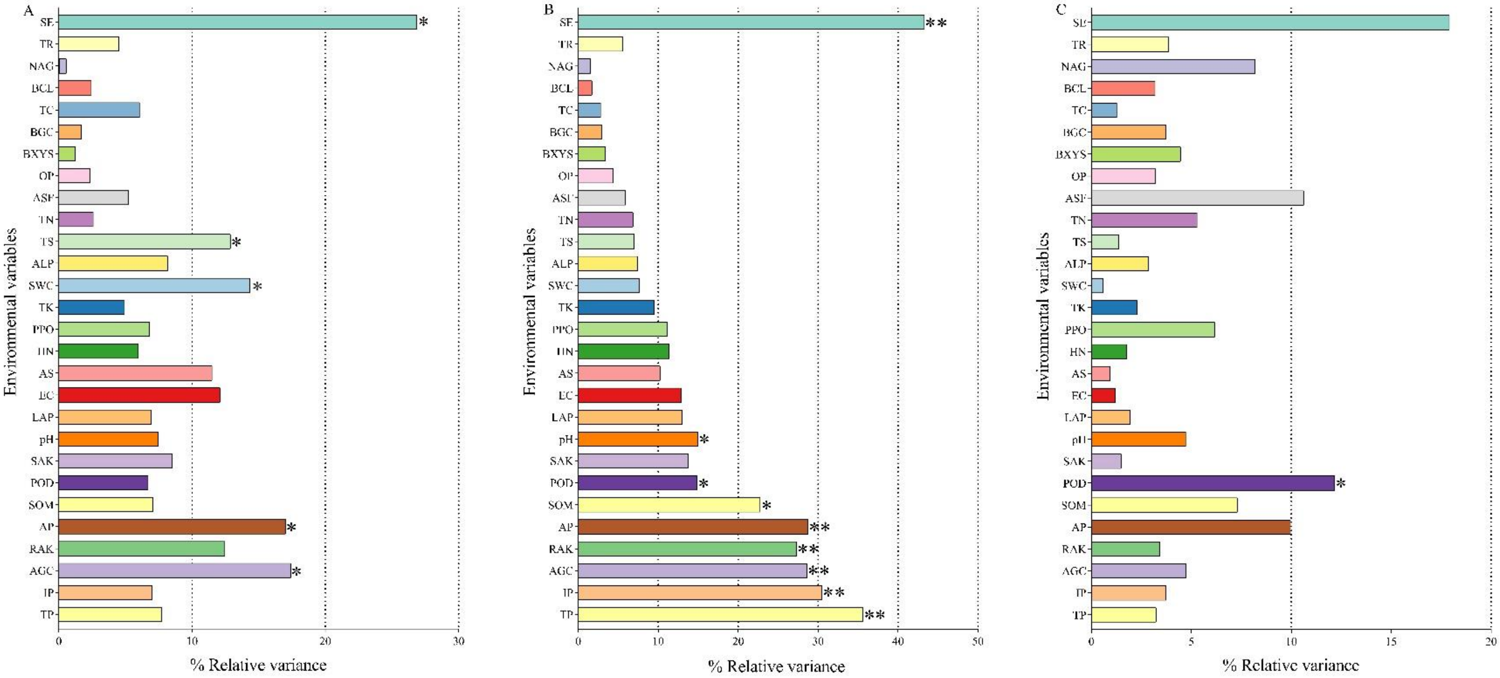

Fig. S12:

The impact of environmental variables on bacterial (A), fungal (B), and protist community compositions using the PERMANOVA test (Bray-Curtis distance matrix). Values less than 0.05 were considered significant.

Supplementary Figures

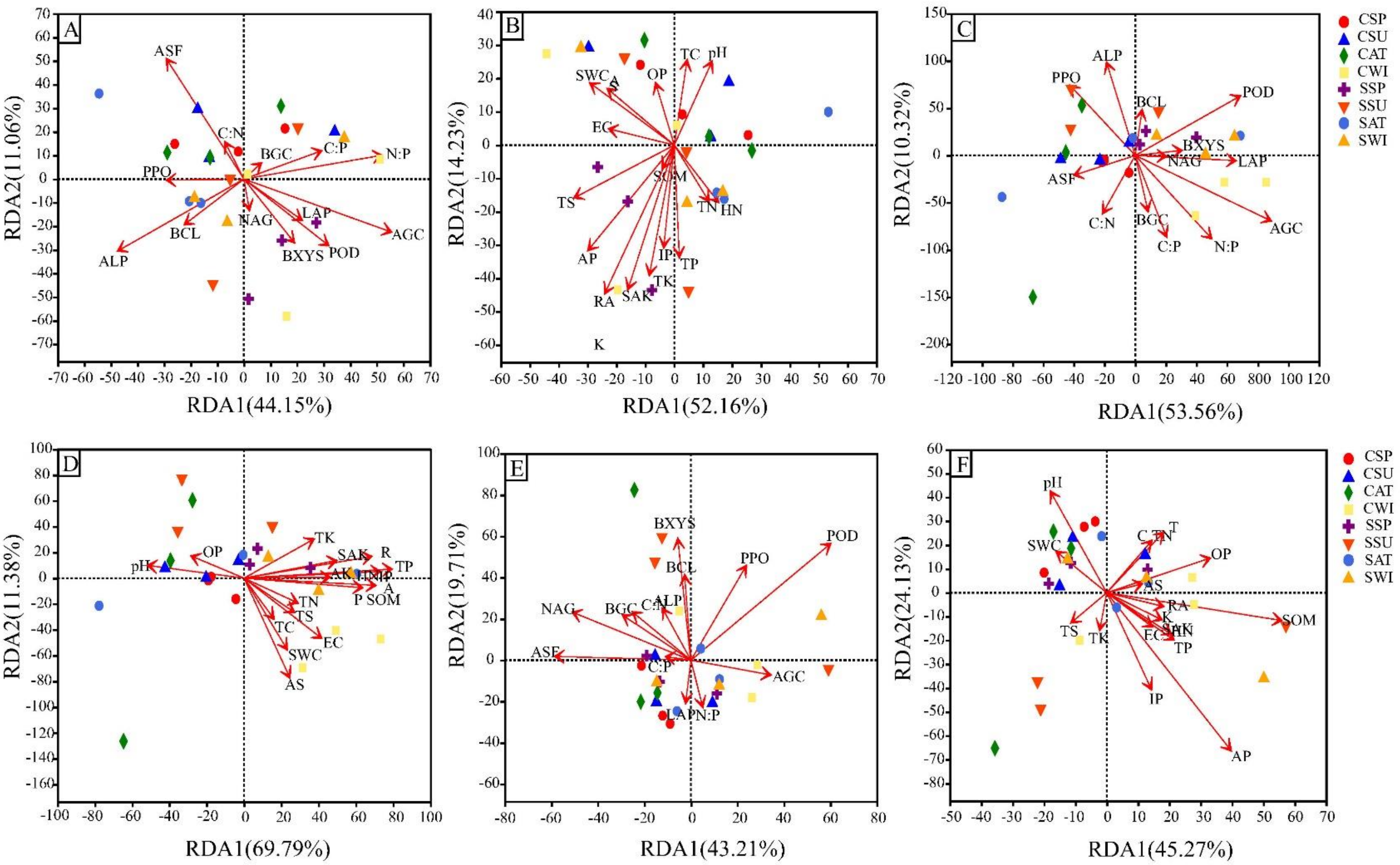

Fig. S13:

RDA is used to establish the linkage of bacterial (A: soil enzymes, B: soil properties), fungal (C: soil enzymes, B: soil properties), and protist (E: soil enzymes, F: soil properties) community compositions with soil enzymes and soil properties.

Supplementary Figures

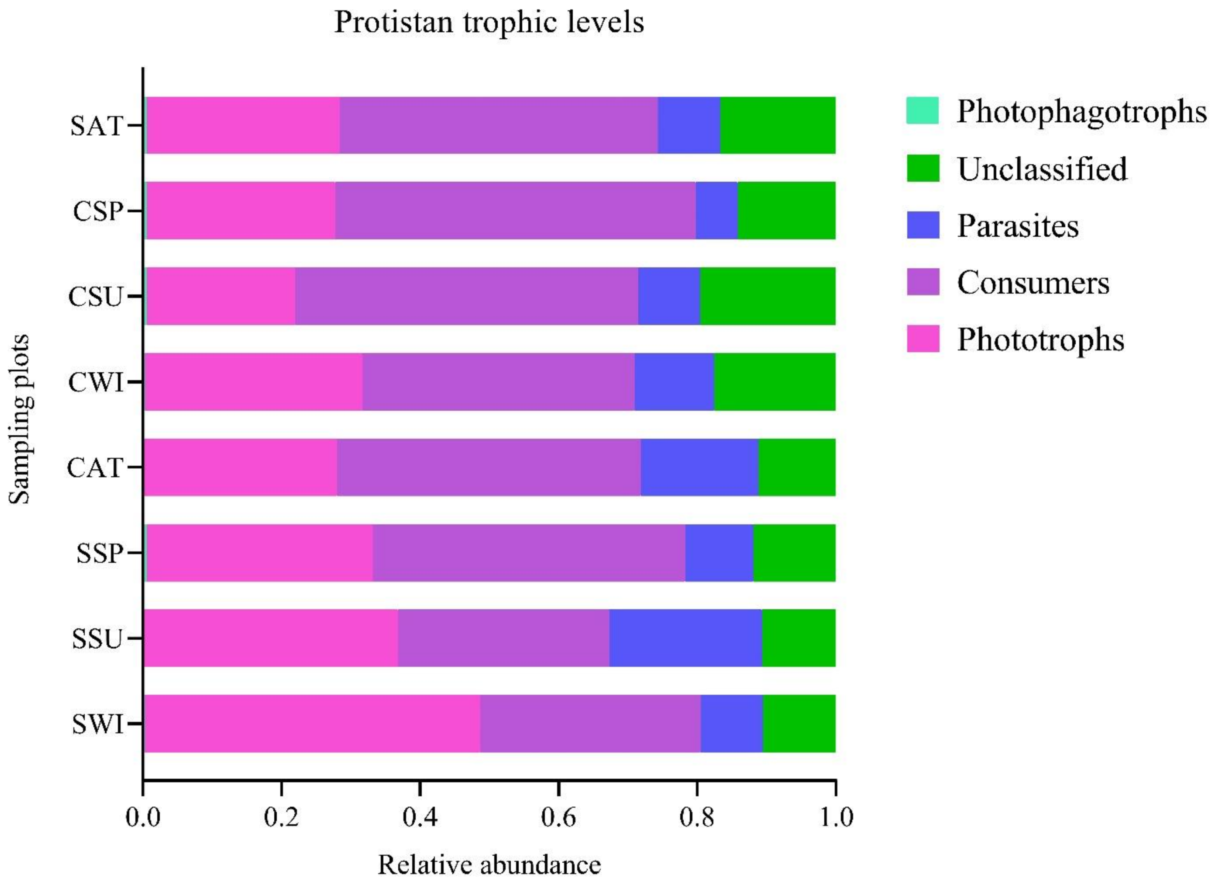

Fig. S14:

Life history strategy (photophagotrophs, parasites, consumers, and phototrophs) of protistan lineages for each plantation type and distinct season. The major protistan lineages were assigned to their dominant mode of energy acquisition: either photophagotrophs, parasites, consumers, or phototrophs.

Abbreviations: C (Chinese pine tree plot), S (Chinese scholar tree plot), SP (spring), SU (summer), AT (autumn), and WI (winter).

Supplementary Figures

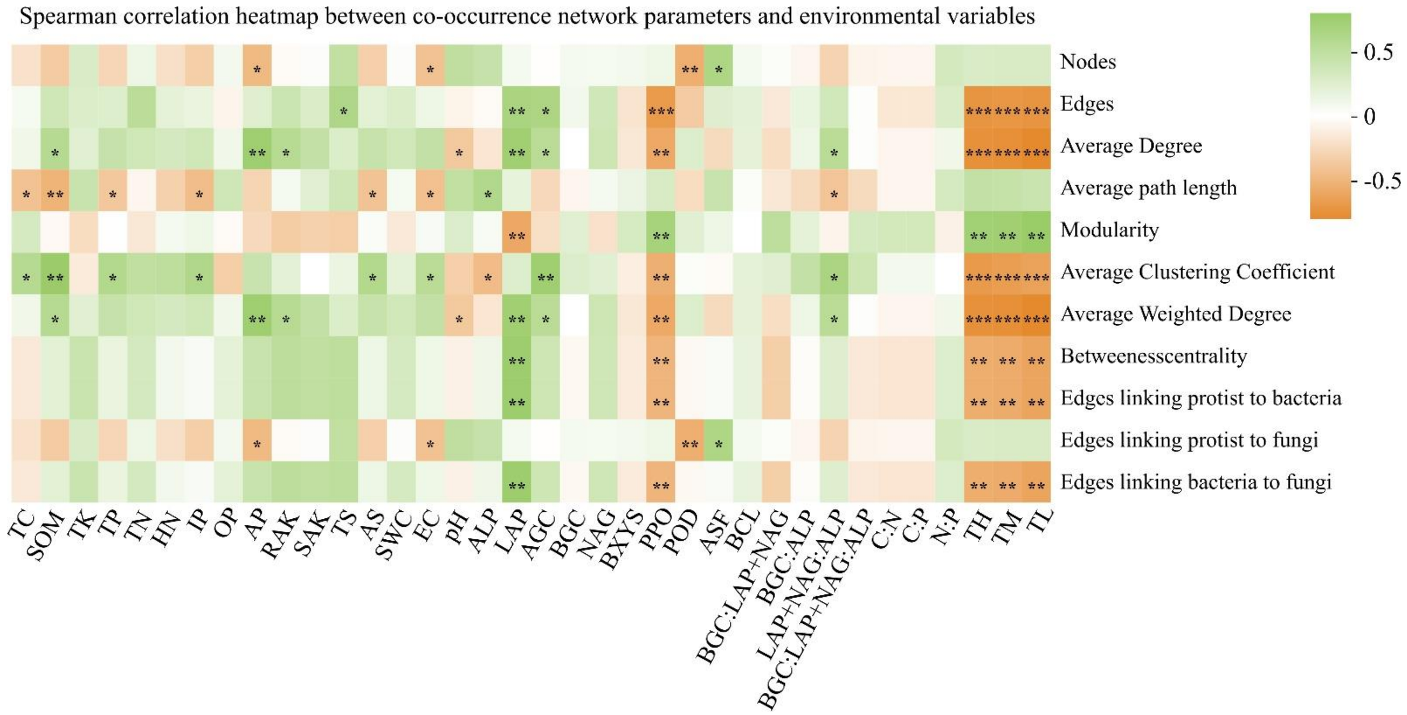

Fig. S15:

The linkage between co-occurrence network parameters and environmental variables. Different network parameters were linked with different environmental variables, among which parameters associated with temperature appeared to be the strongest. Green represents positive correlations, and orange represents negative correlations. * indicates *P*-value < 0.05, ** indicates *P*-value < 0.01, and *** indicates *P*-value < 0.001.

Supplementary Figures

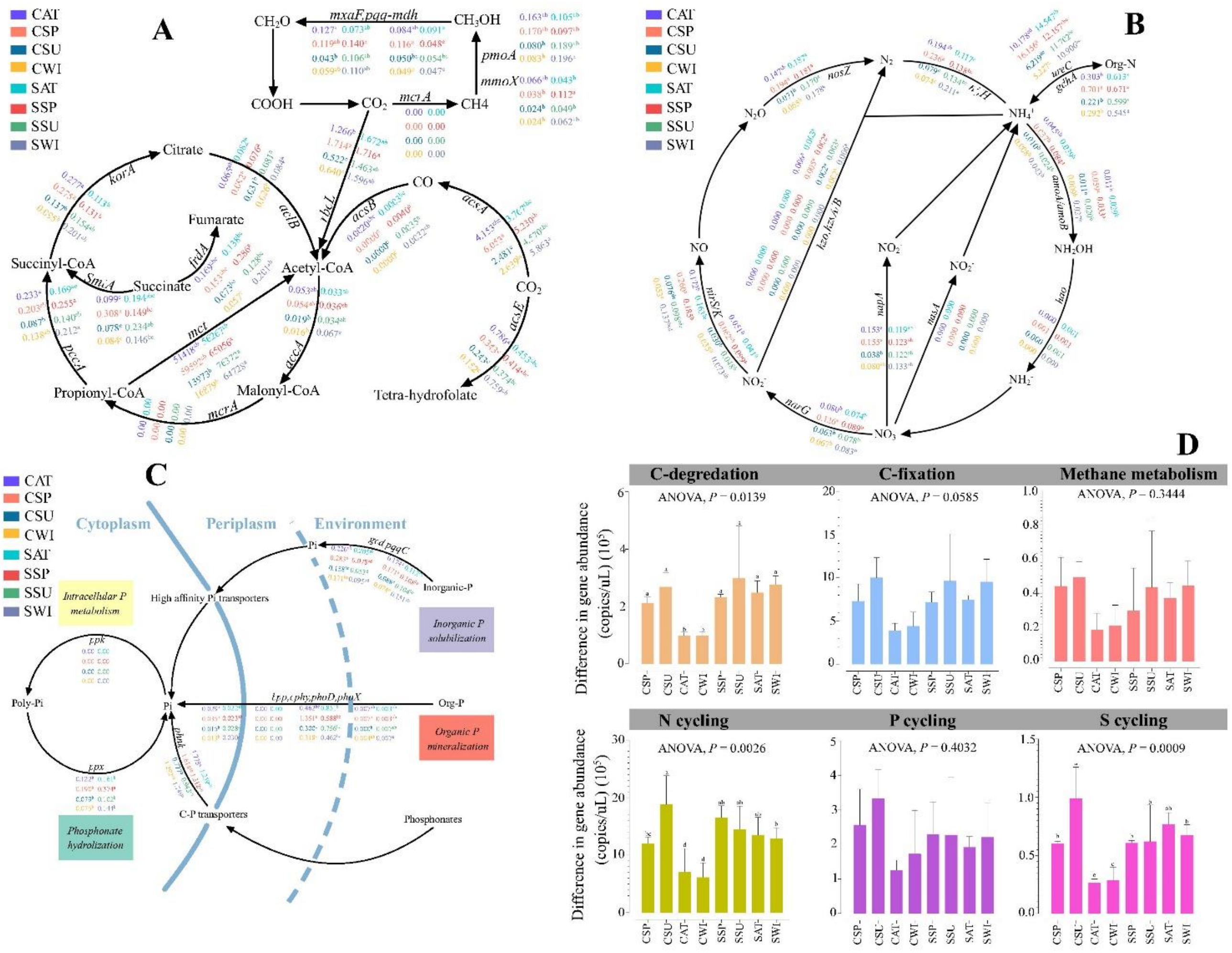

Fig. S16:

Microbe-driven C fixation (A), nitrogen (B), phosphorus biogeochemical processes (C), and functional-gene abundances (D) in different plantation types with four main seasonal variations. Different letters indicate the relative abundance of genes is statistically significant between two afforested plots with seasonal variations. Abbreviations: C (Chinese pine plot tree), S (Chinese scholar plot tree), SP (spring), SU (summer), AT (autumn), WI (winter), and P_i (inorganic orthophosphate).

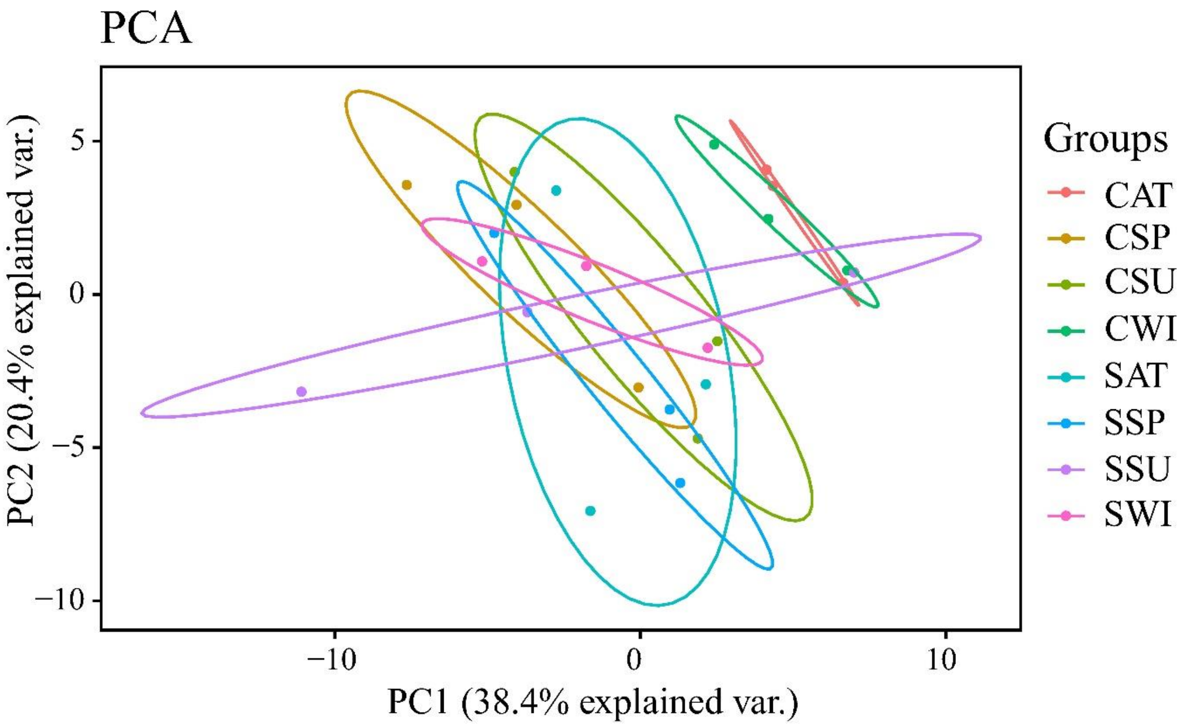

Fig. S17:

Principal Component Analysis (PCA) profile based on the Bray-Curtis distances showing the distinct but concentric patterns of CNPS cycling genes in different plantation types with seasonal variations.

Abbreviations: C (Chinese pine tree plot), S (Chinese Scholar tree plot), SP (spring), SU (summer), AT (autumn), and WI (winter).

Supplementary Figures

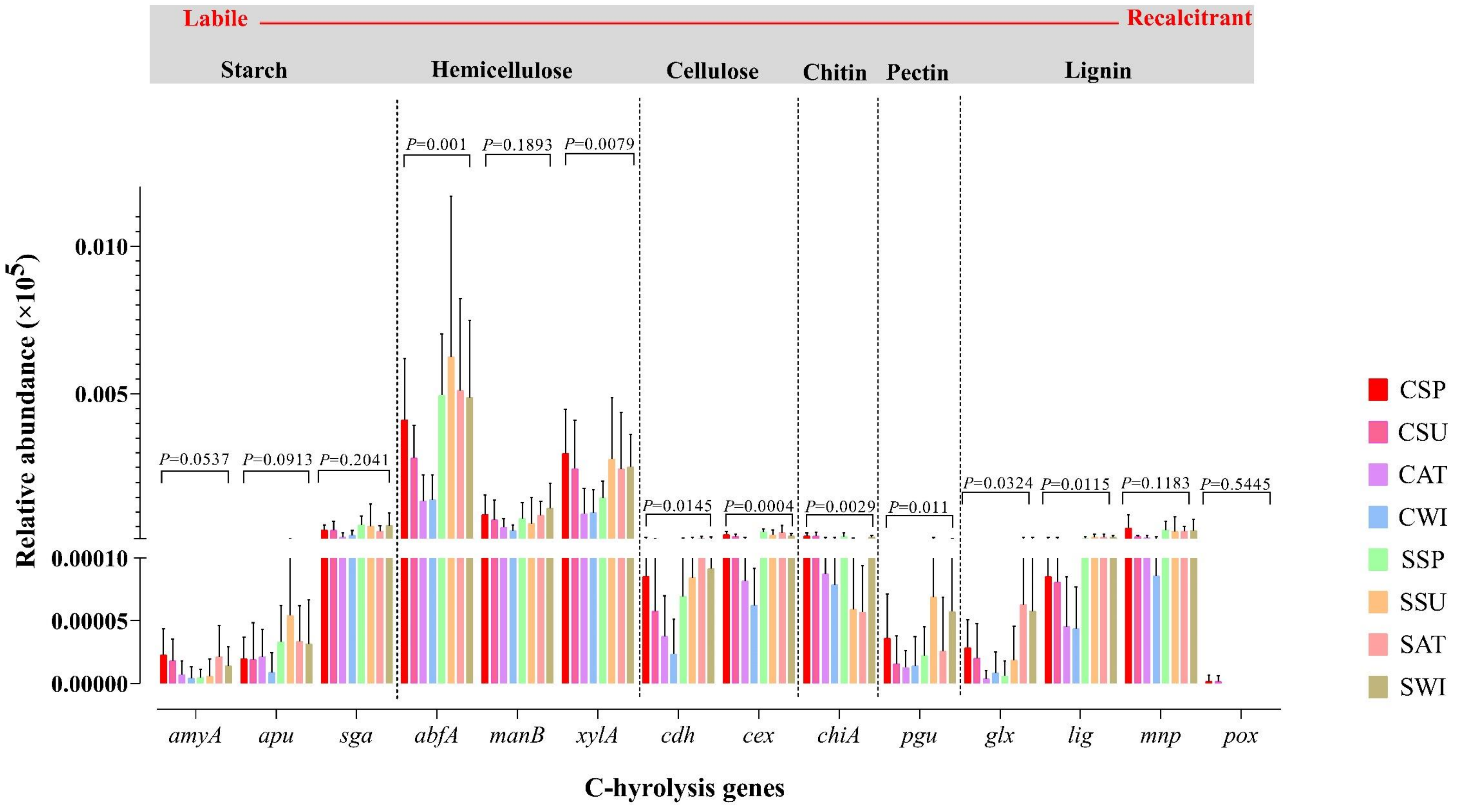

Supplementary Figures

Fig. S18:

The abundance of key-stone C-hydrolysis genes. The potentials for starch degradation, *amyA*, and *apu* were significant, but the absolute abundance of *sga* was not significant in soils ($P = 0.2041$). The result showed that the abundance of C-hydrolysis genes involved in hemicellulose degradation, *abfA* (arabinofuranosidase), *xylA* (xylose isomerase), and *manB* (mannanase), was higher than other C-hydrolysis genes irrespective of plantation types and seasonal variations. The absolute abundance of those genes involved in cellulose, pectin, and chitin statistically differed between plantation types with seasonal variations. Among four genes related to lignin degradation, the absolute abundance of *glx* and *lig* was significantly different, whereas the abundance of *mnp* and *pox* was not statistically different in soils. We should mention that the abundance of the *pox* gene could be detected just in *Pinus* Forest in spring and summer. Some vital genes previously reported (Zheng et al., 2018) were not detected in our study. These genes are comprised of *amyx* (starch degradation), *naglu* (cellulose), and *exo-chi* (chitin).

Supplementary Figures

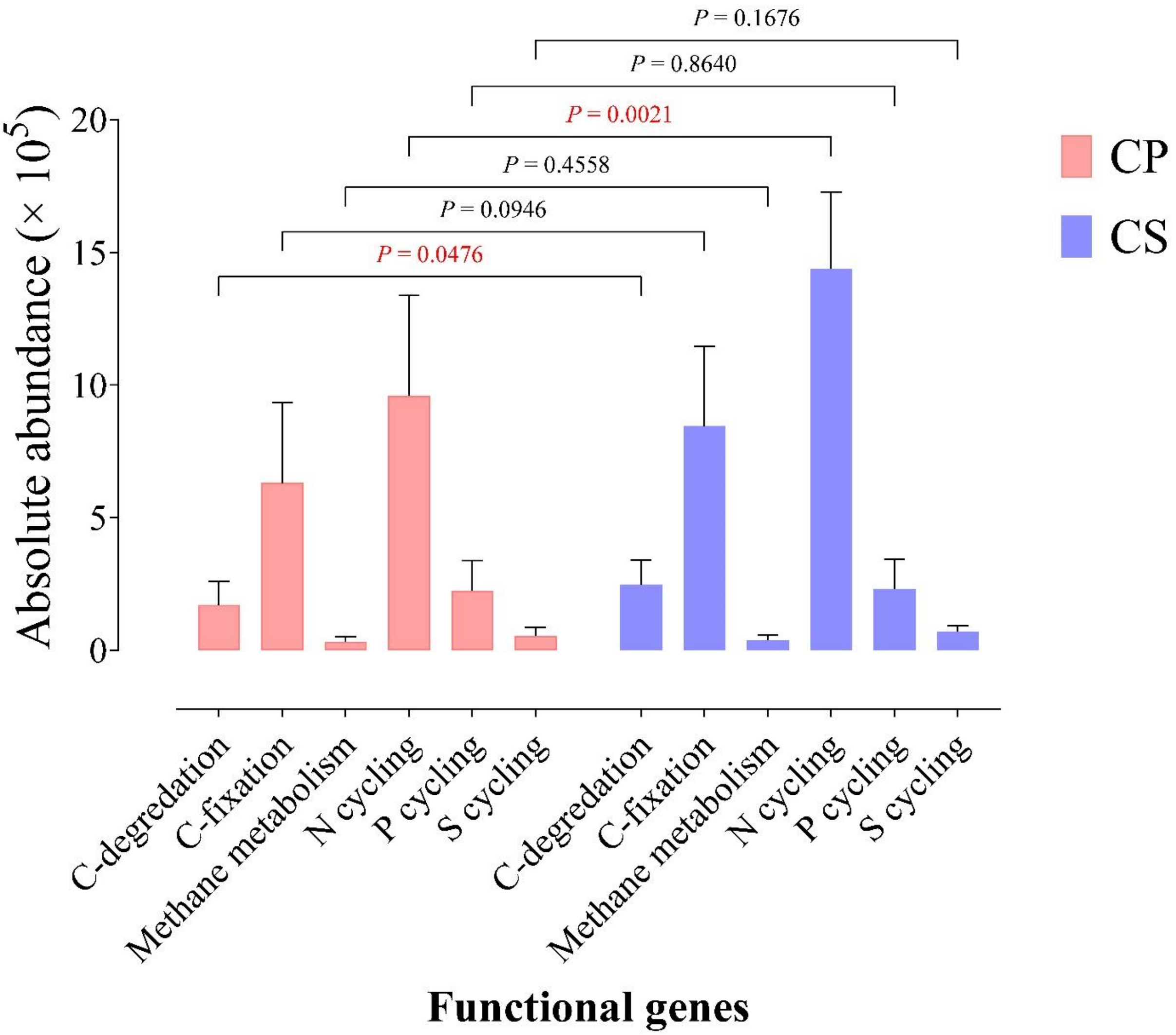

Fig. S19:

There is an abundance of CNPS cycling genes across two different plantation types (ANOVA, HSD-Tukey Kramer test).

Abbreviations: CP (Chinese pine tree plot), CS (Chinese Scholar tree plot)

Supplementary Figures

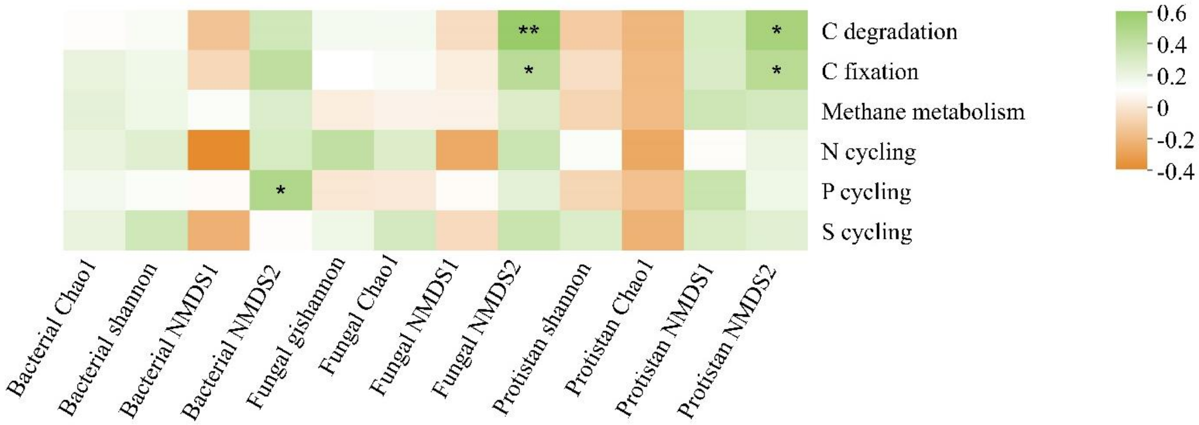

Fig. S20:

Spearman correlation heatmap between microbial diversity indices and CNPS cycle genes.

Supplementary Figures

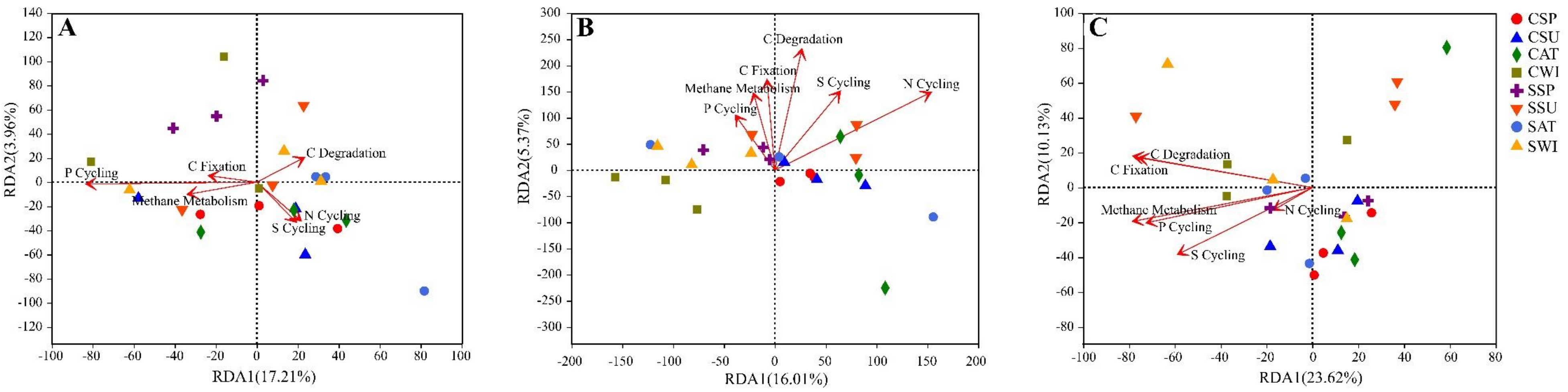

Fig. S21:

The link between CNSP cycling genes with bacterial (A), fungal (B), and protistan (C) community compositions. The RDA results showed that there was not any significant association between the abundance of CNSP cycling genes with bacterial composition in different plantations with seasonal variations [C-degradation (P = 0.835), C-fixation (P = 0.923), Methane metabolism (P = 0.774), N cycling (P = 0.87), P cycling (P = 0.247), and S cycling (P = 0.867)]. The fungal RDA plot showed that the abundance of functional genes involved in C-degradation and N cycling were significantly associated with fungal composition [C-degradation (P = 0.026), C-fixation (P = 0.163), Methane metabolism (P = 0.305), N cycling (P = 0.033), P cycling (P = 0.569), and S cycling (P = 0.156)]. The protistan RDA plot verified that the abundance of C-degradation, C-fixation, and methane metabolism was statistically associated with

the protistan composition in different plantation types seasonally [C-degradation (P = 0.04), C-fixation (P = 0.03), Methane metabolism (P = 0.043), N cycling (P = 0.853), P cycling (P = 0.054), and S cycling (P = 0.115)].

Supplementary Figures

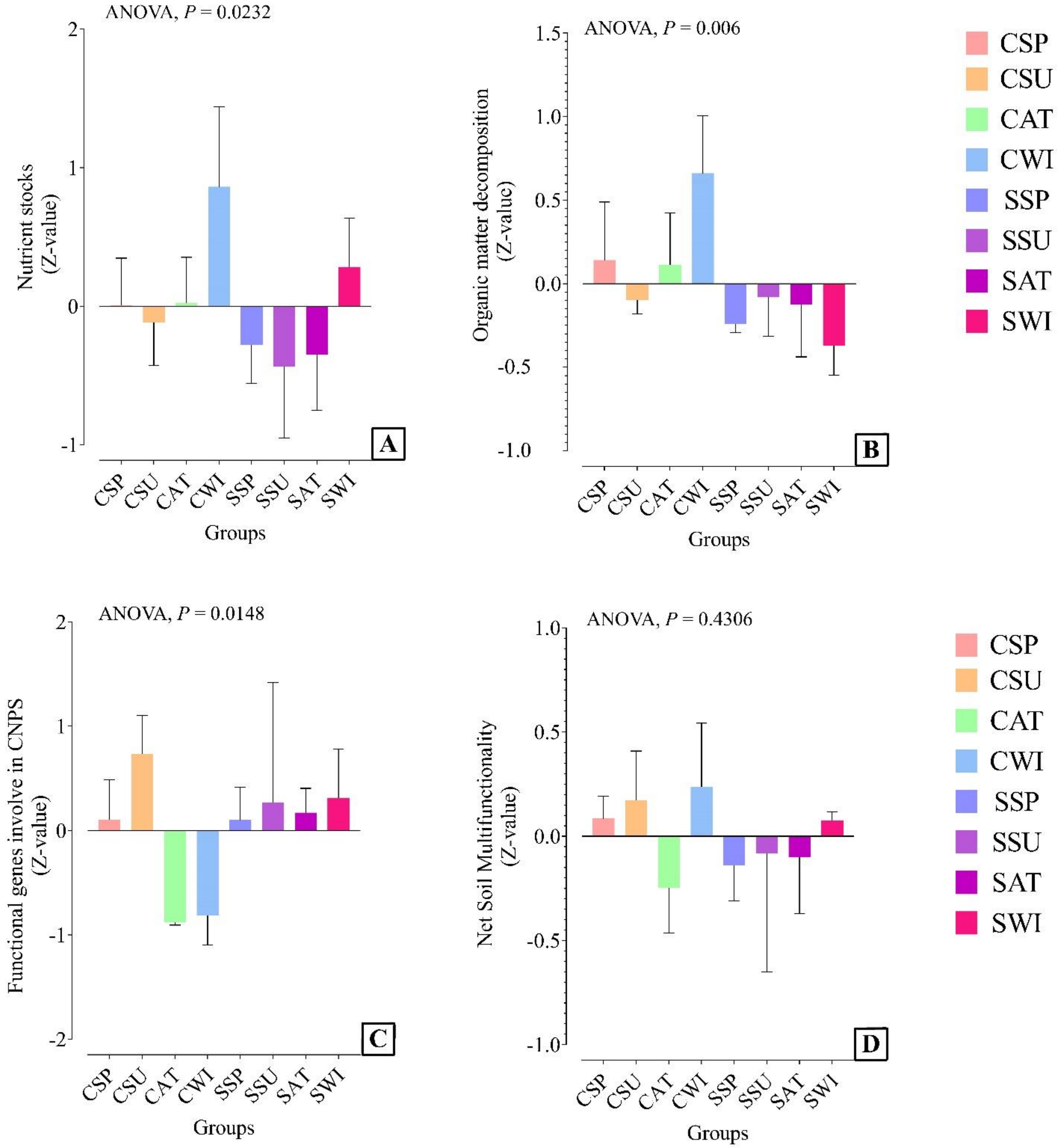

Supplementary Figures

Fig. S22:

Average multifunctionality indices response to different plantations with seasonal variations. The narrow multifunctionality indices like nutrient stocks (A), organic matter decomposition (B), and functional genes involved in CNPS cycling (C) significantly differed within different plantations seasonally, but net soil multifunctionality (D) did not show a significant difference within two plantation types with seasonal variations. This result indicates that the high level of nutrient cycling (nutrient stocks and organic matter decomposition) in winter for the Chinese pine plot compensated by decreases in measures of microbial functional genes involved in CNPS cycling in summer for this plot. Such tradeoffs among functional categories suggest that soils ordinarily may sustain a harmonious level of net soil multifunctionality.

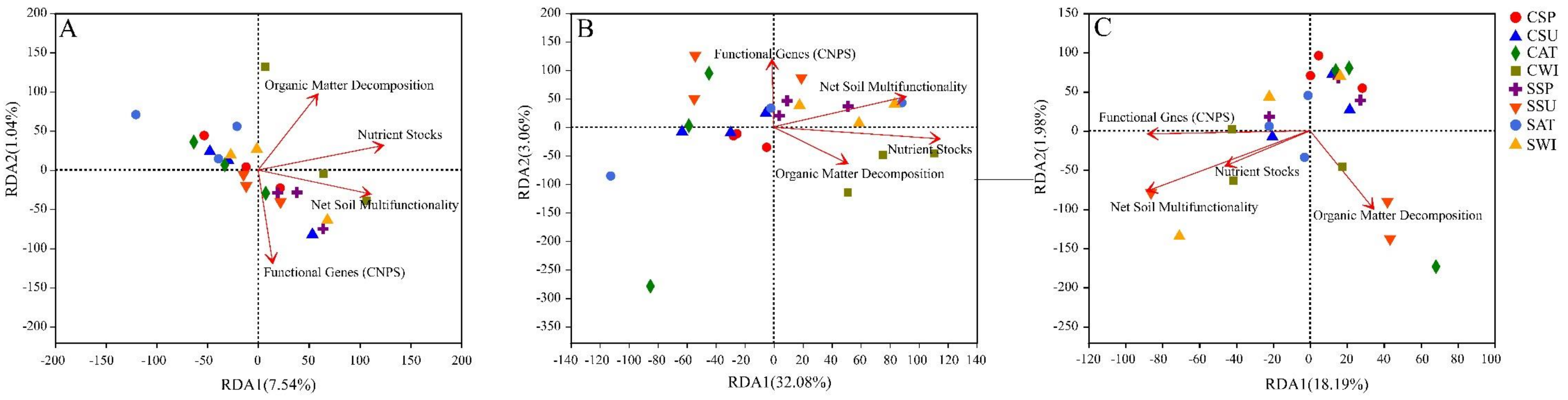

Fig. S23:

The correlation ship between multifunctionality indices and bacterial (A), fungal (B), and protistan (C) compositions. The bacterial RDA showed that 'nutrient stocks' and 'organic matter decomposition' indices were significantly correlated with bacterial composition [Nutrient stocks (P = 0.025), Organic matter decomposition (P = 0.015), Net soil multifunctionality (P = 0.202), Functional genes (CNPS) (P = 0.09)]. The fungal RDA plot demonstrated that the 'nutrient stocks' index was statistically associated with fungal composition [Nutrient stocks (P = 0.006), Organic matter decomposition (P = 0.17), Functional genes (P = 0.051), and net soil multifunctionality (P = 0.053)]. The protistan RDA ordination plot showed that none of the multifunctionality indices were significantly

associated with the protistan community [nutrient stocks (*P* = 0.481), Organic matter decomposition (*P* = 0.348), Functional genes (CNPS) (*P* = 0.077), and net soil multifunctionality (*P* = 0.079).

Supplementary Figures

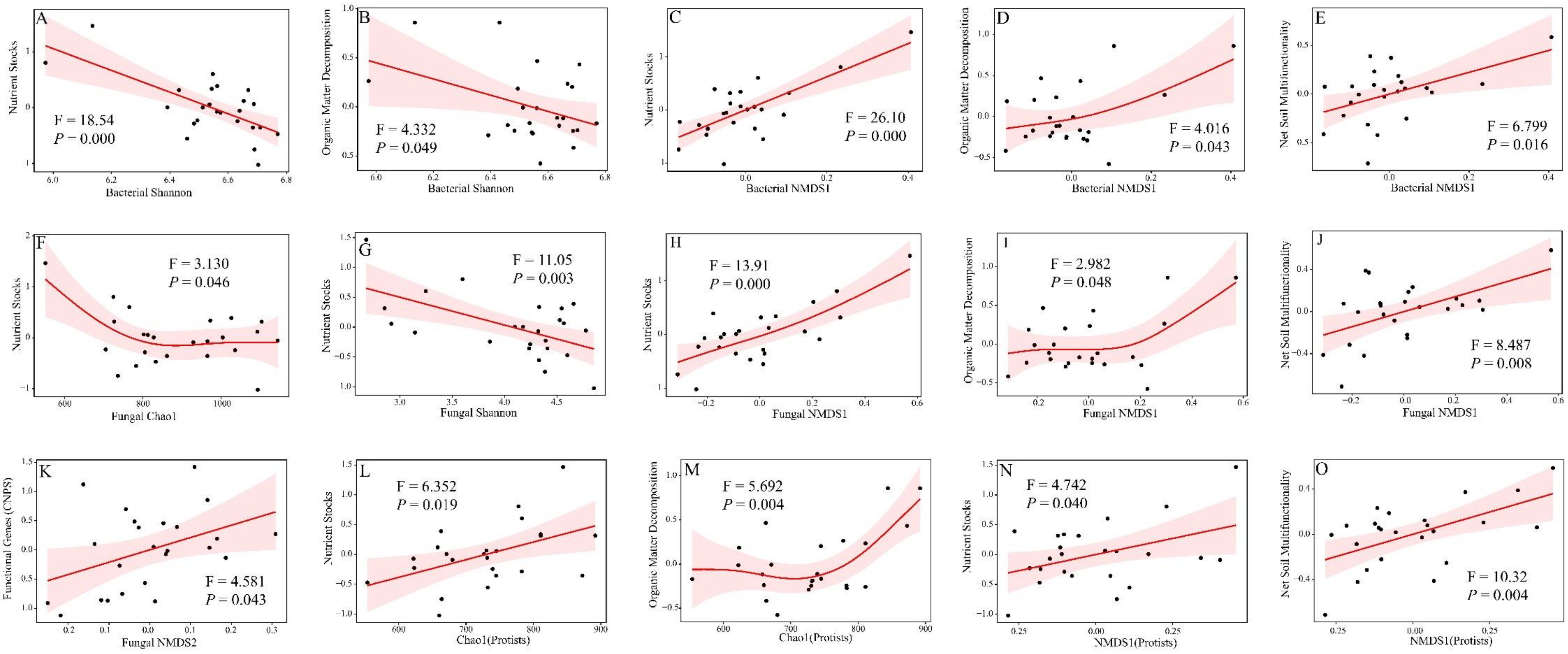

Supplementary Figures

Fig. S24:

Microbial diversity (richness and Shannon diversity)-multifunctionality relationship using the GAM analysis. The results of GAM analysis showed that microbial richness and diversity indices were significantly associated with different functional groups. The results of the GAM analysis showed that the bacterial Shannon diversity was significantly associated with different functional categories like nutrient stocks and organic matter decomposition, and the bacterial β-diversity was statistically associated with nutrient stocks, organic matter decomposition, and net soil multifunctionality. Fungal richness was significantly associated with nutrient stock function, and the fungal β-diversity was statistically linked with four functional groups. Protistan richness was congruent with nutrient stocks and organic matter decomposition, and their β-diversity was linked with nutrient stocks and net soil multifunctionality.

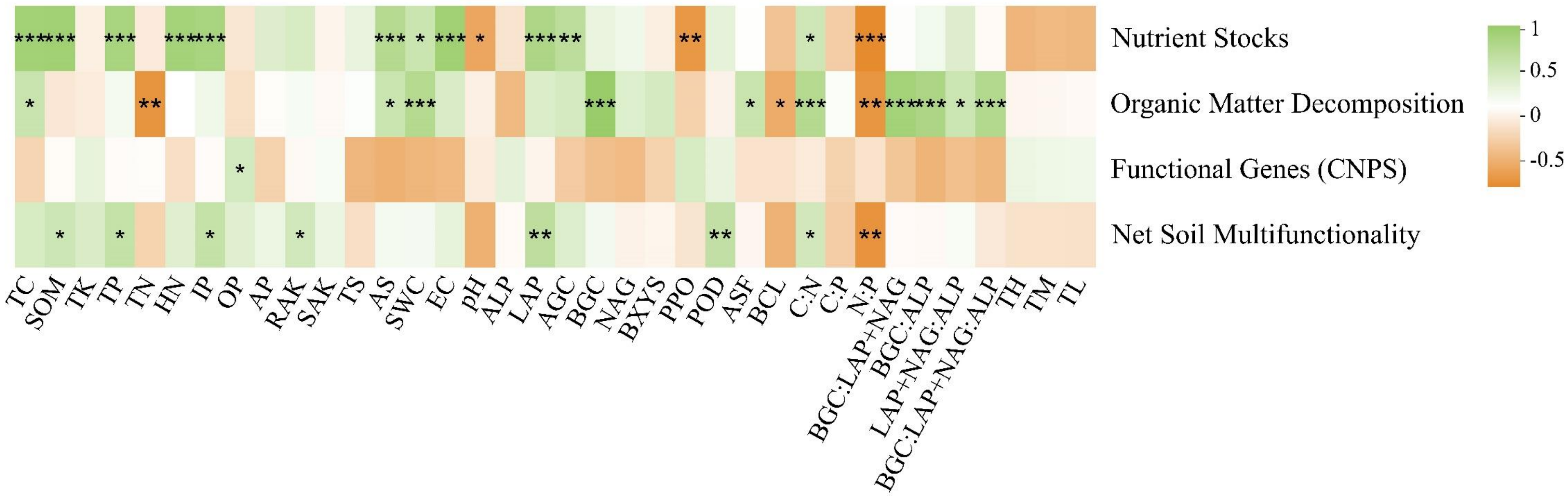

Fig. 25:

Heatmap of environmental driving factors derived multifunctionality indices. As a point of interest, two narrow functional categories, namely, nutrient stocks and organic matter decomposition, were associated with most environmental variables compared with the functional genes (CNPS) category and net soil multifunctionality. * indicates P-value < 0.05, ** indicates P-value < 0.01, and *** indicates P-value < 0.001

Supplementary Figures

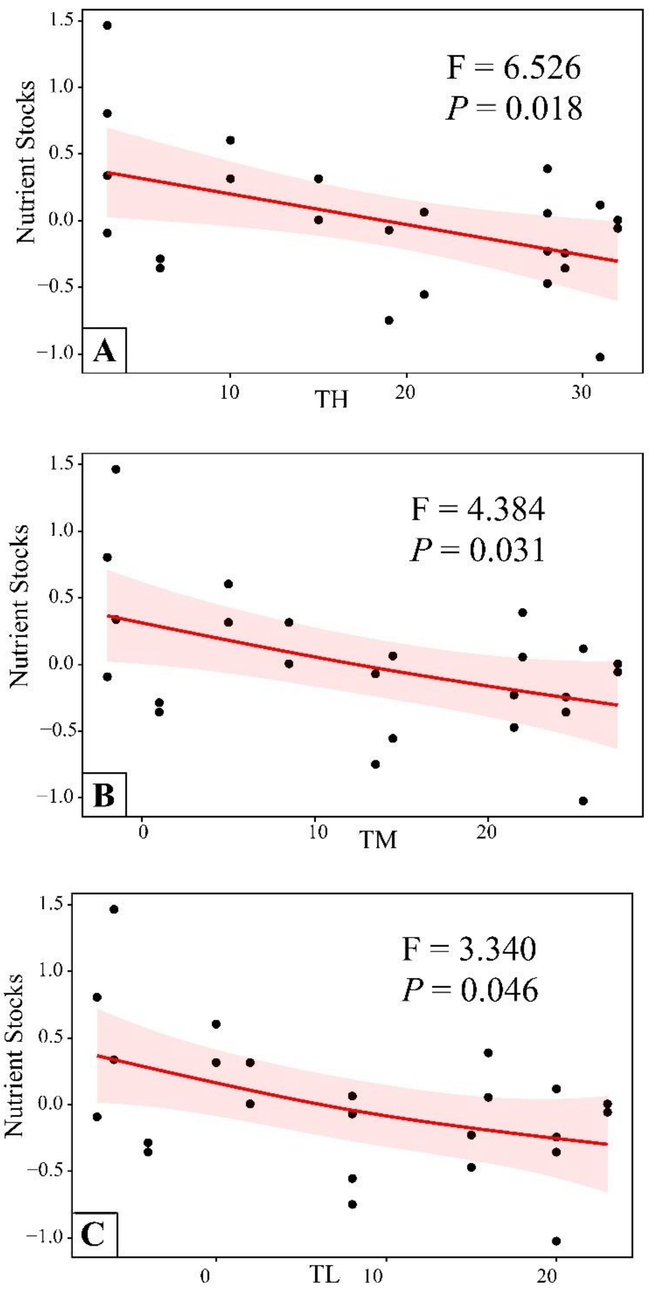

Fig. S26:

The association of multifunctionality categories with annual air temperature. Among all evaluated relationships, just the nutrient stocks category was significantly associated with the minimum, average, and maximum yearly air temperature.

Supplementary Figures

Spearman correlation heatmap between co-occurrence network parameters and functional groups

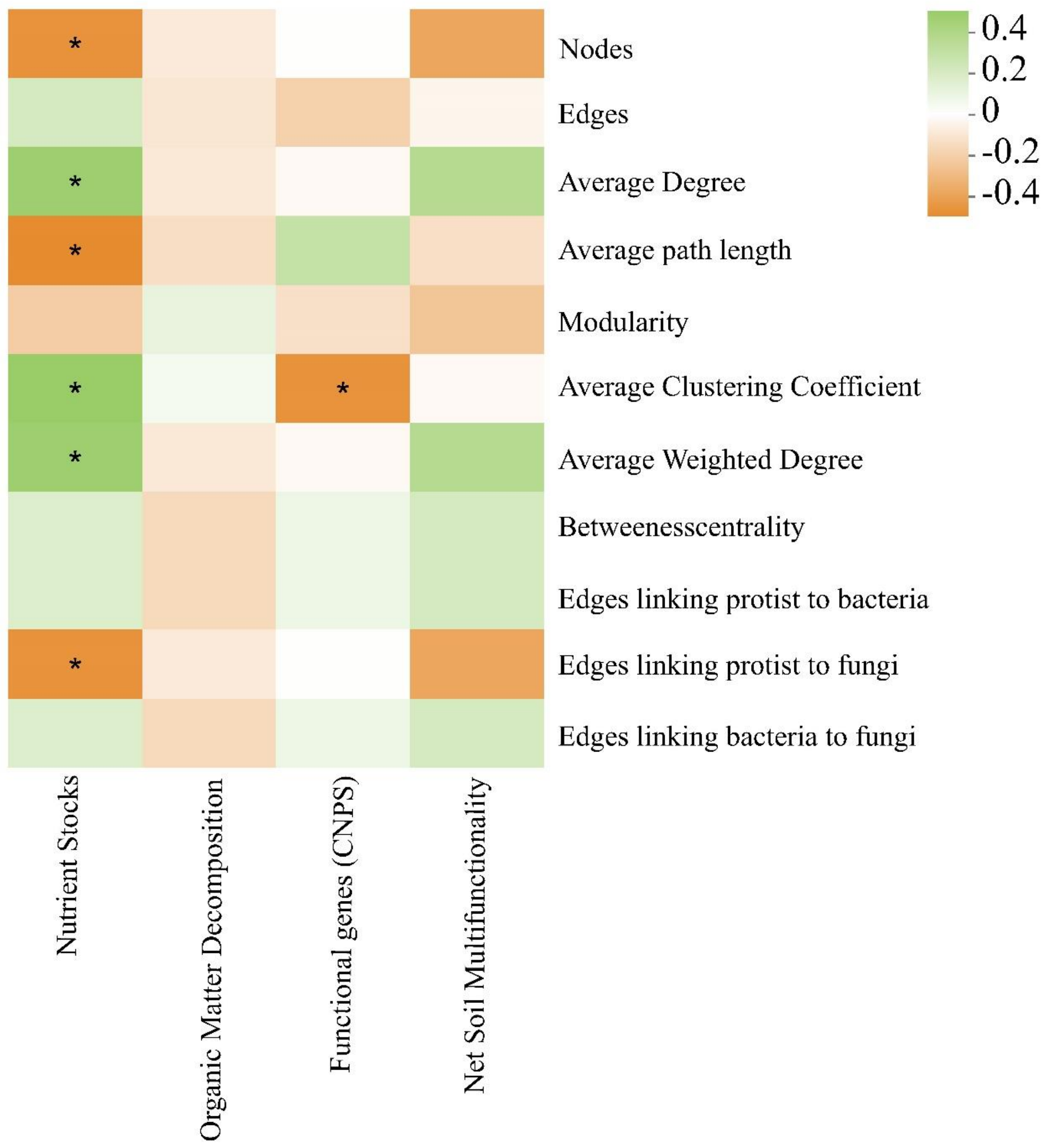

Supplementary Figures

Fig. S27:

The linkage between co-occurrence network parameters and functional groups such as nutrient stocks, organic matter decomposition, functional genes (CNPS), and net soil multifunctionality. The functional group of nutrient stocks is statistically associated with six network parameters, and the microbial functional genes (CNPS) category is significantly associated with the average clustering coefficient. Green represents positive correlations, and orange represents negative correlations. * indicates P-value < 0.05.

Supplementary Figures

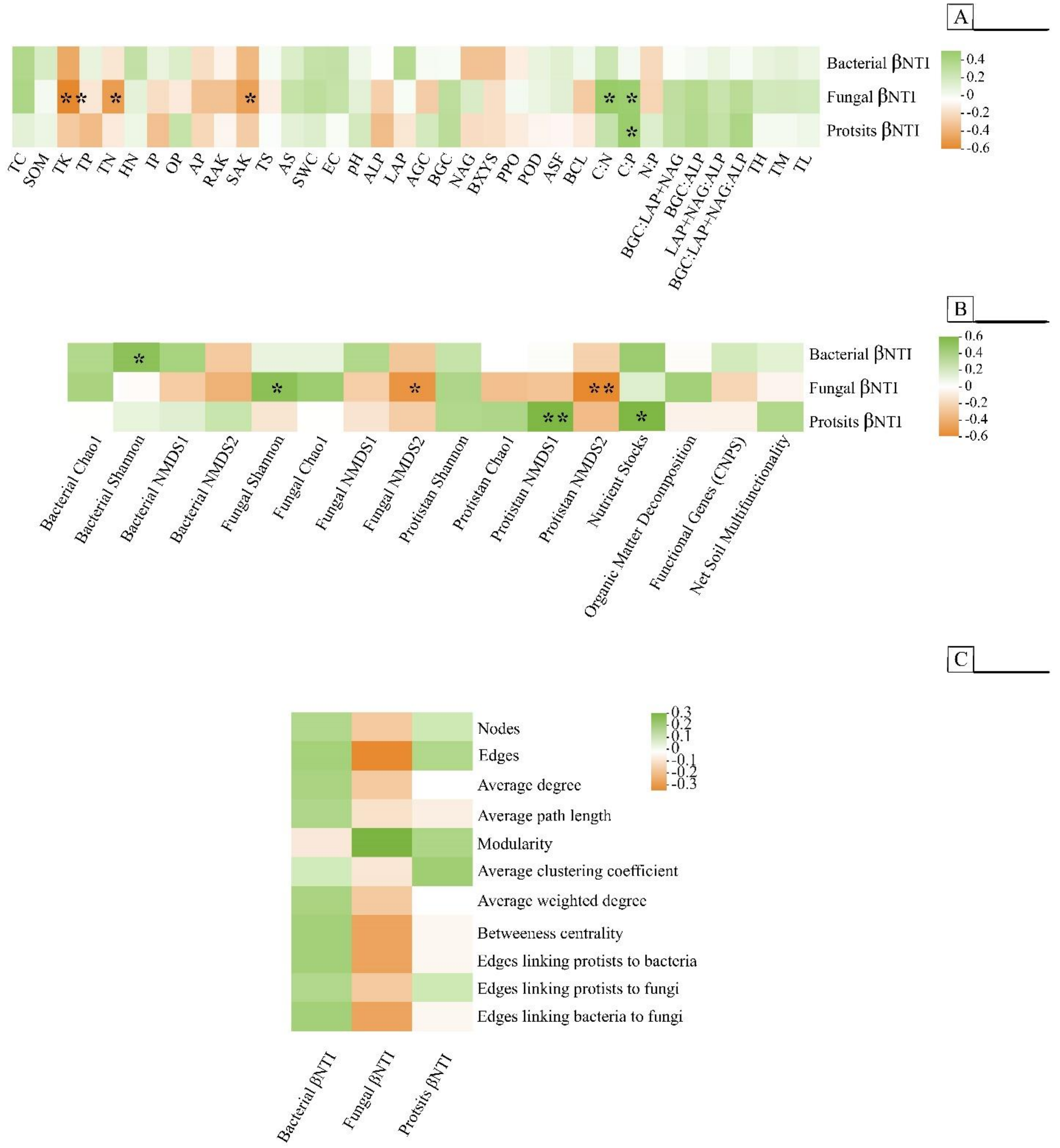

Fig. S28

The linkage between microbial βNTI values with environmental variables (A), microbial diversity indices (α- and β-diversities) (B), and co-occurrence network parameters (C). The bacterial βNTI

value was not associated with environmental variables (A), whereas the protistan βNTI value was statistically linked with the C: P ratio. The fungal βNTI value was significantly congruent with several environmental variables like the C: P ratio, the C: N ratio, SAK, TN, and TK (). The βNTI value was significantly associated with the bacterial Shannon diversity (B). The fungal βNTI value was linked with the protistan NMDS2, the fungal NMDS2, and the fungal Shannon diversity. The protistan βNTI value was significantly correlated with nutrient stocks and the protistan NMDS1. None of the co-occurrence network parameters were associated with microbial βNTI values (C). Green represents positive correlations, and orange represents negative correlations. * indicates *P*-value < 0.05, and ** indicates *P*-value < 0.01.

Supplementary Figures

References

Behie, S.W., Moreira, C.C., Sementchoukova, I., Barelli, L., Zelisko, P.M., and Bidochka, M.J., (2017). Carbon translocation from a plant to an insect-pathogenic endophytic fungus. Nat. Commun. 8 (1), 14245. DOI: https://doi.org/10.1038/ncomms14245.

Boerner, R.E.J., Brinkman, J.A., and Smith, A., (2005). Seasonal variations in enzyme activity and organic carbon in soil of a burned and unburned hardwood forest. Soil Biol. Biochem. 37 (8), 1419-1426. DOI: https://doi.org/10.1016/j.soilbio.2004.12.012.

Caporaso, J.G., Lauber, C.L., Walters, W.A., Berg-Lyons, D., Lozupone, C.A., Turnbaugh, P.J., Fierer, N., and Knight, R., (2011). Global patterns of 16S rRNA diversity at a depth of millions of sequences per sample. Proc. Natl. Acad. Sci. 108 (Supplement 1), 4516-4522. DOI: https://doi.org/10.1073/pnas.1000080107.

Criquet, S., Farnet, A.M., Tagger, S., and Le Petit, J., (2000). Annual variations of phenoloxidase activities in an evergreen oak litter: influence of certain biotic and abiotic factors. Soil Biol. Biochem. 32 (11), 1505-1513. DOI: https://doi.org/10.1016/S0038-0717(00)00027-4.

Decker, K.L., Boerner, R.E., and Morris, S.J., (1999). Scale-dependent patterns of soil enzyme activity in a forested landscape. Can. J. For. Res. 29 (2), 232-241. DOI: https://doi.org/10.1139/x98-192.

Finzi, A.C., Sinsabaugh, R.L., Long, T.M., and Osgood, M.P., (2006). Microbial community responses to atmospheric carbon dioxide enrichment in a warm-temperate forest. Ecosystems 9 (2), 215-226. DOI: https://doi.org/10.1007/s10021-005-0078-6.

Gardes, M., and Bruns, T.D., (1993). ITS primers with enhanced specificity for basidiomycetes--application to the identification of mycorrhizae and rusts. Mol. Ecol. (2), 113-118. DOI: 10.1111/j.1365-294x.1993.tb00005.x.

Huse, S.M., Dethlefsen, L., Huber, J.A., Welch, D.M., Relman, D.A., and Sogin, M.L., (2008). Exploring Microbial Diversity and Taxonomy Using SSU rRNA Hypervariable Tag Sequencing. PLoS Genet. 4 (11), e1000255. DOI: https://doi.org/10.1371/journal.pgen.1000255.

Kellner, H., Luis, P., Schlitt, B., and Buscot, F., (2009). Temporal changes in diversity and expression patterns of fungal laccase genes within the organic horizon of a brown forest soil. Soil Biol. Biochem. 41 (7), 1380-1389. DOI: https://doi.org/10.1016/j.soilbio.2009.03.012.

Rimington, W.R., Pressel, S., Duckett, J.G., Field, K.J., and Bidartondo, M.I., (2019). Evolution and networks in ancient and widespread symbioses between Mucoromycotina and liverworts. Mycorrhiza 29 (6), 551-565. DOI: https://doi.org/10.1007/s00572-019-00918-x.

Santos, S.S., Nielsen, T.K., Hansen, L.H., and Winding, A., (2015). Comparison of three DNA extraction methods for recovery of soil protist DNA. J. Microbiol. Methods 115, 13-19. DOI: https://doi.org/10.1016/j.mimet.2015.05.011.

Santos, S.S., Schöler, A., Nielsen, T.K., Hansen, L.H., Schloter, M., and Winding, A., (2020). Land use as a driver for protist community structure in soils under agricultural use across Europe. Sci. Total Environ. 717, 137228. DOI: https://doi.org/10.1016/j.scitotenv.2020.137228.

Sinsabaugh, R.L., (2010). Phenol oxidase, peroxidase and organic matter dynamics of soil. Soil Biol. Biochem. 42 (3), 391-404. DOI: https://doi.org/10.1016/j.soilbio.2009.10.014.

Sinsabaugh, R.L., Gallo, M.E., Lauber, C., Waldrop, M.P., and Zak, D.R., (2005). Extracellular enzyme activities and Soil organic matter dynamics for northern hardwood forests receiving simulated nitrogen deposition. Biogeochemistry 75 (2), 201-215. DOI: https://doi.org/10.1007/s10533-004-7112-1.

Sinsabaugh, R.L., Saiya-Cork, K., Long, T., Osgood, M.P., Neher, D.A., Zak, D.R., and Norby, R.J., (2003). Soil microbial activity in a Liquidambar plantation unresponsive to CO2-driven increases in primary production. Appl. Soil Ecol. 24 (3), 263-271. DOI: https://doi.org/10.1016/S0929-1393(03)00002-7.

Toberman, H., Freeman, C., Evans, C., Fenner, N., and Artz, R.R.E., (2008). Summer drought decreases soil fungal diversity and associated phenol oxidase activity in upland Calluna heathland soil. FEMS Microbiol. Ecol. 66 (2), 426-436. DOI: https://doi.org/10.1111/j.1574-6941.2008.00560.x.

Ullah, S., Ai, C., Huang, S., Zhang, J., Jia, L., Ma, J., Zhou, W., and He, P., (2019). The responses of extracellular enzyme activities and microbial community composition under nitrogen addition in an upland soil. PLoS One 14 (9), e0223026. DOI: https://doi.org/10.1371/journal.pone.0223026.

Weintraub, M.N., Scott-Denton, L.E., Schmidt, S.K., and Monson, R.K., (2007). The effects of tree rhizodeposition on soil exoenzyme activity, dissolved organic carbon, and nutrient availability in a subalpine forest ecosystem. Oecologia 154 (2), 327-338. DOI: https://doi.org/10.1007/s00442-007-0804-1.

White Tj, Bruns Td, Lee Sb, and Jw, T. 1990. Amplification and direct sequencing of fungal ribosomal RNA genes for phylogenetics. United States: Academic Press

Supplementary Tables

Table S1: Information on seasonal temperature and precipitation

Seasons	Average temperature (℃)	Daily maximum temperature (℃)	Daily minimum temperature (℃)	Precipitation (mm)
Spring	-1.5	4.89	-6.19	0.25
Summer	14.81	21.22	8.43	0.87
Autumn	26.05	31.32	21.47	6.36
Winter	13.38	19.69	8.44	1.18

Supplementary Tables

Table S2: Procedure for evaluation of soil elemental parameters

Soil parameters	Methods	References
TP	NaOH Alkali Fusion-Molybdenum Antimony Spectrophotometry	* GB/T 9837-1988
TK	Sodium Hydroxide Melting - Flame Photometric Method	* NY/T 87-1988
HN	Alkaline Diffusion Method	* LY/T 1229-1999
AP	Sodium Bicarbonate Solution Extraction, Molybdenum Antimony Colorimetric Method	* NY/T 1121.7-2014
RAK	Ammonium Acetate Extraction-Flame Photometer Method	* NY/T 889-2004
SAK	Hot Nitric Acid Extraction-Atomic Absorption Method	* NY/T 889-2004
OP	Burning Method	Bao, S.D. (2000)
IP	1mol/L Hydrochloric Acid Extraction, Molybdenum Antimony Colorimetric Method	Medeiros, JJ et al., (2005)
SOM	Potassium Dichromate Oxidation-External Heating Method	* NY/T 1121.6-2006
TC	Foil Embedding, TOC Analyzer Measurement (Soil-Determination of organic carbon-Combustion oxidation nondispersive infrared absorption method)	* HJ 695-2014
TS	Nitric Acid-Perchloric Acid Digestion, ICP-AES	Zhang et al., 2017
AS	CaCl2 Extractant Extraction, Barium Sulfate Turbidimetric Method	* NY/T 1121.14-2006
TN	Sulfuric Acid-Accelerator Digestion, Kjeldahl Method (Nitrogen determination methods of forest soils)	* LY/T 1228-2015
pH	Determination of pH in Soil	COMBI 5000 (STEP Systems GmbH, Duisburger, Nuremberg, Germany)
EC	Determination of EC in Soil	COMBI 5000 (STEP Systems GmbH, Duisburger, Nuremberg, Germany)
SWC	Drying Method (Determination of forest soil water content)	* LY/T 1213-1999

* China's Soil Standard protocols for forest regions in Chinese

Supplementary Tables

Bao, S.D. (2000) Soil agricultural chemical analysis. 3rd Edition, China Agricultural Press, Beijing, 265-267.

Medeiros, JJ González, B. Pérez Cid, and E. Fernández Gómez. "Analytical phosphorus fractionation in sewage sludge and sediment samples." *Analytical and bioanalytical chemistry* 381, no. 4 (2005): 873-878.

Zhang et al., 2017 Improvement of Extraction and Determination Method of Total Sulfur in Soil, Journal of Anhui Agricultural Sciences.

Supplementary Tables

Table S3: Extracellular enzyme abbreviations

Nutrient cycle	Enzyme	Abbreviation	EC	Reactions
C cycle	β-Glucosidase	BGC	3.2.1.21	Hydrolyze glucose from cellobiose and cellulose oligomers (cellulose degradation)
	β- xylosidase	BXYS	3.2.1.37	Hydrolyze xylose from hemicellulose and extracellular polysaccharides
	β-cellobiosidase	BCL	3.2.1.91	Hydrolysis of (1→4)-β-D-glucosidic linkages in cellulose and cellotetraose
N cycle	Leucine Aminopeptidase	LAP	3.4.11.1	Hydrolyses leucine and other hydrophobic amino acids from the N terminus of polypeptides
	α-Glucosidase	AGC	3.2.1.20	Starch degradation
	N-acetyl-β-D-glucosidase	NAG	3.2.1.52	Chitin and peptidoglycan degradation
P cycle	Alkaline Phosphatase	ALP	3.1.3.1	Mineralize organic P into phosphate
S cycle	Aryl sulfatase	ASF	3.1.6.1	Catalyze 3-oxo-L-alanine
Phenolic compounds oxidase	Polyphenol oxidase	PPO	1.14.18.1	Oxidize phenols using oxygen
	Peroxidase	POD	1.11.1.17	Oxidize aromatic and aliphatic hydrocarbons using peroxide

This extracellular enzyme category is selected based on the study of Ullah et al. (2019). EC denotes the enzyme's commission number.

Ullah, S., Ai, C., Huang, S., Zhang, J., Jia, L., Ma, J., Zhou, W., and He, P., 2019. The responses of extracellular enzyme activities and microbial community composition under nitrogen addition in upland soil. PLOS ONE 14, e0223026 DOI: https://doi.org/10.1371/journal.pone.0223026.

Supplementary Tables

Table S4: The qPCR primers and PCR cycle conditions in this study

Domain	Primers	Sequences (5' to 3')	Thermal cycles	References	Amplification size
Bacteria	338F	ACTCCTACGGGAGGCAGCA	95 °C pre-denaturation for 3 min, 35 × (30 s at 95 °C, 5 s at 58 °C, 1 min at 72 °C) 10 min at 72 °C	(Huse et al., 2008)	~ 445 bp
	806R	GGACTACHVGGGTWTCTAAT		(Caporaso et al., 2011)	
Fungi	ITS1F	CTTGGTCATTTAGAGGAAGTAA	95 °C pre-denaturation for 3 min, 35 × (30 s at 95 °C, 5 s at 62 °C, 1 min at 72 °C) 10 min at 72 °C	(Gardes and Bruns, 1993)	~ 290 bp
	ITS2R	GCTGCGTTCTTCATCGATGC		(White TJ et al., 1990)	
Eukaryote	Euk345F	AAGGAAGGCAGCAGGCG	95 °C pre-denaturation for 3 min, 35 × (30 s at 95 °C, 5 s at 62 °C, 1 min s at 72 °C), 10 min at 72 °C	(Santos et al., 2015; 2020)	~ 150 bp
	Euk499R	CACCAGACTTGCCCTCYAAT			

Melt Curve 65.0 °C to 95.0 °C, increment 0.5 °C

Caporaso, J.G., Lauber, C.L., Walters, W.A., Berg-Lyons, D., Lozupone, C.A., Turnbaugh, P.J., Fierer, N., and Knight, R., 2011. Global patterns of 16S rRNA diversity at a depth of millions of sequences per sample. Proc. Natl. Acad. Sci. 108, 4516-4522 DOI: https://doi.org/10.1073/pnas.1000080107.

Gardes, M., and Bruns, T.D., 1993. ITS primers with enhanced specificity for basidiomycetes--application to the identification of mycorrhizae and rusts. Mol. Ecol., 113-118 DOI: https://doi.org/10.1111/j.1365-294x.1993.tb00005.x.

Huse, S.M., Dethlefsen, L., Huber, J.A., Welch, D.M., Relman, D.A., and Sogin, M.L., 2008. Exploring Microbial Diversity and Taxonomy Using SSU rRNA Hypervariable Tag Sequencing. PLoS Genet. 4, e1000255 DOI: https://doi.org/10.1371/journal.pgen.1000255.

Santos, S.S., Nielsen, T.K., Hansen, L.H., and Winding, A., 2015. Comparison of three DNA extraction methods for recovery of soil protist DNA. J. Microbiol. Methods 115, 13-19 DOI: https://doi.org/10.1016/j.mimet.2015.05.011.

Santos, S.S., Schöler, A., Nielsen, T.K., Hansen, L.H., Schloter, M., and Winding, A., 2020. Land use as a driver for protist community structure in soils under agricultural use across Europe. Sci. Total Environ. 717, 137228 DOI: https://doi.org/10.1016/j.scitotenv.2020.137228.

White Tj, Bruns Td, Lee Sb, and Jw, T. (1990). Amplification and direct sequencing of fungal ribosomal RNA genes for phylogenetics. United States: Academic Press.

Supplementary Tables

Table S5: Information of 72 CNPS cycling genes detected in gene chip

Number	Gene Name	Forward Primer	Reverse Primer	Classification
1	16S rRNA	GTGCCAGCMGCCGCGG	CCGTCAATTCMTTTRAGTTT	
2	abfA	CGSTAYCCSGGCGGCAAYTT	TGCCASGGNCCGTCCATYTC	C degradation
3	amyA	YGGTTTTCGTCTTGACGCSG	MGGCTGMGTRTCATGRTTK	C degradation
4	amyX	TATAAYTGGGGMTATGAYCC	CCCATYAAATCAAAWCGRAA	C degradation
5	apu	ACVTGGATAGGYGAGCCYCA	CCRTCSGGGAAGTAGTTKCC	C degradation
6	cdh	ATWRYCTWCCGMRTHGCCMT	GTKAGSGGRTTBYKGRYCAT	C degradation
7	chiA	TSAAGAARTACGCSGACAACG	ASGTCATCAGRCCCTTSAG	C degradation
8	exo-chi	GATTGGTSVCAATATGAYRG	STCCARCCACCRAYRCTRAA	C degradation
9	cex	YSTACGGSATGCACTGGMT	TANCGCAGRTAGTCVCCCAT	C degradation
10	glx	AACCAGTCGATCATCTACGA	RTGSACGAGCTCDGGCATGG	C degradation
11	iso-plu	GTCATYTACTTYGGNCC	CGNGCSACATCNGCCCA	C degradation
12	lig	CCGCACACACTGTTGCTGC	CGAAGGATTGCCACTCGCA	C degradation
13	manB	ATGCGCGGBGTCAACCA	TCGTTGSCGATGTTGABGA	C degradation
14	mnp	MACRCCSTTCGACTCSACC	ACGTCSGAGCAGTCRAYGA	C degradation
15	naglu	TVAAYTGGTAYCTGAAATAY	CCRTGYAGVGCCATCCAGTC	C degradation
16	pgu	ANCATTGGTGGCCSTGGAA	TTRAYGGCRATRCARTCRTC	C degradation
17	pox	ACYAGTATCCATTGGCACGGT	AGATGVGARTGATACCARAA	C degradation
18	xylA	TGGGGBGGTCGYGAAGG	ACTTTGGCRTCRAAGTT	C degradation
19	accA	GAAGGCTAYCGCAARGC	CCTTCMGGSGARATMAC	C fixation
20	aclB	TGGACMATGGTDGCYGGKGGT	ATAGTTKGGSCCACCTCTTC	C fixation
21	acsA	GATACCTGGTGGCAGACCGA	TGATCACGTCGTCGACCCGG	C fixation
22	acsB	CTYTGYCAGTCMTTYGCBCC	CCCATAAABCCYGGDGTYTG	C fixation
23	acsE	TCATCGGCGAACGCATCAAC	AGRCCGGCTTCSATGGC	C fixation
24	cdaR	CGARATGGTGGTGCTCAA	CARCGTRTTACGATGAATA	C fixation
25	frd A	MTGCTGCACACSCTGTW	CCGGTSGGGTGRWACTG	C fixation
26	korA	GCCGGCTACCCCATCACCCC	ATGATGGGATGGTCGCCATG	C fixation
27	mcrA	GGTGGTGTMGGDTTCACMCARTA	CGTTCATBGCGTAGTTVGGRTAGT	C fixation
28	mct	TGGGCGCSGASGTSATMCG	TTGACSGTRTARTCSAYSGC	C fixation
29	pccA	GTGMTGATCAAGGCCWC	CGSGTGTTCATYTCSAGGAA	C fixation
30	rbcL	AAGGACGACGAGAACATC	TGCAGGATCATGTCGTT	C fixation
31	smtA	TTTCTGGCCGGBTAYGCDGC	CGGTACGGHCCGGTYTGVCC	C fixation
32	ureC	AAGMTSCACGAGGACTGGGG	AGRTGGTGGCASACCATSAGCAT	Ammonification
33	hzo	AAGACNTGYCAYTGGGGWAAA	GACATACCCATACTKGTRTANACNGT	Anaerobic ammonium oxidation
34	hzsA	WTYGGKTATCARTATGTAG	AAABGGYGAATCATARTGGC	Anaerobic ammonium oxidation
35	hzsB	ARGGHTGGGGHAGYTGGAAG	GTYCCHACRTCATGVGTCTG	Anaerobic ammonium oxidation
36	nasA	CARCCNAAYGCNATGGG	ATNGTRTGCCAYTGRTC	Assimilatory N reduction
37	narG	TAYGTSGGGCAGGARAAACTG	CGTAGAAGAAGCTGGTGCTGT	Denitrification
38	nirK1	GGMATGGTKCCSTGGCA	GCCTCGATCAGRTTRTGGTT	Denitrification
39	nirK2	ATGGCGCCATCATGGTNYTNCC	TCGAAGGCCTCGATNARRTTRTG	Denitrification
40	nirK3	TGCACATCGCCAACGGNATGTWYGG	GGCGCGGAAGATGSHRTGRTCNAC	Denitrification
41	nirS1	GTSAACGTSAAGGARACSGG	GASTTCGGRTGSGTCTTGA	Denitrification
42	nirS2	ATCGTCAACGTCAARGARACVGG	TTCGGGTGCGTCTTSABGAASAG	Denitrification
43	nirS3	TGGAGAACGCCGGNCARGTNTGG	GATGATGTCCACGGCNACRTANGG	Denitrification
44	nosZ1	CGYTGTTCMTCGACAGCCAG	CGSACCTTSTTGCCSTYGCG	Denitrification
45	nosZ2	CGCRACGGCAASAAGGTSMSSGT	CAKRTGCAKSGCRTGGCAGAA	Denitrification
46	napA	CTGGACIATGGGYTTIAACCA	CCTTCYTTYTCIACCCACAT	Dissimilatory N reduction
47	ppk	GACCCGAABGTRCTBGCSAT	TTATAATTNCCSGTNCCNA	Inorganic P biosynthesis
48	ppx	TGCATCTGGCGGACGGCCT	AGATCCGCCGCCAATATCA	Inorganic P hydrolysis
49	gcd	CGSAACTGGGAYTACCGS	TCCCACAGSCCSKCGTC	Inorganic P solubilization
50	gcd	ATCGCGTTCGGGCCGGACG	ATSAGRTTSAGCTCGTCCCA	Inorganic P solubilization
51	pqqC	AACCGCTTCTACTACCAG	GCGAACAGCTCGGTCAG	Inorganic P solubilization
52	pqq-mdh	TGTTCTATGTGCCGGCCAA	CTTCCACAGTTCCTTGCC	Methane metabolism
53	mmox	ATGGAGGCGGTCAAGGACGA	CGCTTCATGCCCTTCCACAG	Methane metabolism
54	mxaF	GCGGCACCAACTGGGGCTGGT	GGGCAGCATGAAGGGCTCCC	Methane metabolism
55	pmo A	GGNGACTGGGACTTCTGG	GAASGCNGAGAAGAASGC	Methane metabolism
56	nifH	AAAGGYGGWATCGGYAARTCCACCAC	TGSGCYTTGTCYTCRCGGATBGGCAT	N fixation
57	amoA1	STAATGGTCTGGCTTAGACG	GCGGCCATCCATCTGTATGT	Nitrification
58	amoA2	GGGGTTTCTACTGGTGGT	CCCCTCKGSAAAGCCTTCTT	Nitrification
59	amoB	TGGTAYGACATKAWATGG	RCGSGGCARGAACATSGG	Nitrification
60	hao	TGTCATACCCGGCACAAGTTC	CATRTGGCAGAACTGRCABGT	Nitrification
61	nxrA	CAGACCGACGTGTGCGAAAG	TCCACAAGGAACGGAAGGTC	Nitrification
62	gdh A	GCCATCGGYCCWTACAAGGG	ATGTCRCCNGCCGGAACGTC	Organic N mineralization
63	bpp	GACGCAGCCGAYGAYCCNGCNITNTGG	CAGGSCGCANRTCIACRTTRTT	Organic P mineralization
64	cphy	GTGGACCTRCGRMARGARWCICA	GTCCGACCATTGCCTGCYTCRCARTGRAMRTGIADCCA	Organic P mineralization

Supplementary Tables

65	phnk	CATCGTCGGCGAATCCGG	TGCTGCATGCCGCCGGAAAA	Organic P mineralization
66	phoD	CAGTGGGACGACCACGAGGT	GAGGCCGATCGGCATGTCG	Organic P mineralization
67	phoX	GARGAGAACWTCCACGGYTA	GATCTCGATGATRTGRCCRAAG	Organic P mineralization
68	soxY	ATCGATGACAACCCCGTGCC	AGCTGGTCCATCTGCATGCCG	S oxidation
69	yedz	CTGCTGATCACGCTGGCCAT	GCGATGCAGCTTCTTCCAGCG	S oxidation
70	apsA	GGGYCTKTCCGCYATCAAYAC	ATCATGATCTGCCAGCGGCCGGA	S reduction
71	dsrA	ACSCACTGGAAGCACG	GGTGGAGCCGTGCATGTT	S reduction
72	dsrB	CAACATCGTYCAYACCCAGGG	GTGTAGCAGTTACCGCA	S reduction

Supplementary Tables

Table S6: Ecological attributes within each soil functional group

**Overall (broad function)**	**Functional groups (narrow functions)**	**Attributes**	**Function role**	**Measurement**
Net soil multifunctionality	Nutrient stocks	TC (g/kg)	Carbon pool	Soil chemical analysis
		SOM (g/kg)	Carbon pool	Soil chemical analysis
		TK (g/kg)	Potassium pool	Soil chemical analysis
		TP (g/kg)	Phosphorus pool	Soil chemical analysis
		TN (g/kg)	Nitrogen pool	Soil chemical analysis
		HN (g/kg)	Nitrogen pool	Soil chemical analysis
		IP (g/kg)	Phosphorous pool	Soil chemical analysis
		OP (g/kg)	Phosphorous pool	Soil chemical analysis
		AP (g/kg)	Phosphorous pool	Soil chemical analysis
		RAK (g/kg)	Potassium pool	Soil chemical analysis
		SAK (g/kg)	Potassium pool	Soil chemical analysis
		TS (g/kg)	Sulfur pool	Soil chemical analysis
		AS (g/kg)	Sulfur pool	Soil chemical analysis
		SWC (%)	Water content	Soil chemical analysis
		EC (ms/cm)	Electrical conductivity	Soil chemical analysis
		C:N	Carbon: nitrogen ratio	Soil chemical analysis

Supplementary Tables

Organic matter decomposition	ALP (μmol/d/g)	Alkaline phosphatase (P cycle)	Soil protein analysis
	LAP (μmol/d/g)	Leucine Aminopeptidase (C cycle)	Soil protein analysis
	AGC (μmol/d/g)	α-Glucosidase (N cycle)	Soil protein analysis
	BGC (μmol/d/g)	β-Glucosidase (C cycle)	Soil protein analysis
	NAG (μmol/d/g)	N-acetyl-β-D-glucosidase (N cycle)	Soil protein analysis
	BXYS (μmol/d/g)	β- xylosidase (C cycle)	Soil protein analysis
	PPO (mg/d/g)	Polyphenol oxidase (Phenolic compounds oxidase)	Soil protein analysis
	POD (mg/d/g)	Peroxidase (Phenolic compounds oxidase)	Soil protein analysis
	ASF (μmol/d/g)	Arylsulfatase (S cycle)	Soil protein analysis
	BCL (μg /min /g)	β-cellobiosidase (C cycle)	Soil protein analysis
	C:P [ln (BGC): ln (ALP)]	Stoichiometry	Soil protein analysis
	C:N [ln (BGC): ln (NAG + LAP)]	Stoichiometry	Soil protein analysis
	N:P [ln (NAG + LAP): ln (ALP)]	Stoichiometry	Soil protein analysis
	ln C: ln N: ln P	Stoichiometry	Soil protein analysis
Microbial functional genes	C cycle (35 genes)	Carbon hydrolysis, Starch, Hemicellulose, Cellulose, Chitin, Pectin, Lignin, C fixation	Amplicon sequencing
	N cycle (22 genes)	N fixation, Nitrification, Denitrification, Ammonification, Anaerobic ammonium oxidation, Assimilatory N reduction, Dissimilatory N reduction, Organic N mineralization	Amplicon sequencing

Supplementary Tables

	P cycle (9 genes)	Organic P mineralization, Inorganic P solubilization, Inorganic P biosynthesis, Inorganic P hydrolysis	Amplicon sequencing
	S cycle (5 genes)	S reduction, S oxidation	Amplicon sequencing
	Methane metabolism (4 genes)	Methane production, Methane oxidation	Amplicon sequencing
	Phylogenetic marker (1 gene)	16S rRNA gene	Amplicon sequencing

Supplementary Tables

Table S7: The impact of plantation types with seasonal variations on soil properties and enzyme activities in the XNA

Samples	CSP	CSU	CAT	CWI	SSP	SSU	SAT	SWI
TC (g/kg)	18.79±0.33^{a}	18.79±0.60^{a}	19.07±0.49^{a}	19.54±0.81^{a}	16.90±1.21^{b}	17.18±1.18^{b}	18.74±0.56^{a}	18.69±0.60^{a}
SOM (g/kg)	14.35±0.86^{abc}	13.78±0.18^{bc}	13.82±1.19^{abc}	15.26±1.36^{ab}	13.62±0.87^{abc}	13.24±1.30^{c}	14.39±0.45^{abc}	15.38±0.12^{a}
TK (g/kg)	19.49±1.06^{ab}	18.84±0.33^{b}	18.57±0.33^{b}	19.06±0.69^{ab}	20.73±1.33^{a}	20.39±1.65^{ab}	19.21±0.47^{ab}	19.62±1.62^{ab}
TP(g/kg)	0.84±0.06	0.86±0.01	0.84±0.07	0.92±0.04	0.86±0.08	0.83±0.08	0.9±0.12	0.93±0.07
TN(g/kg)	0.97±0.03^{bc}	0.95±0.04^{bc}	0.95±0.05^{bc}	0.95±0.04^{bc}	1.02±0.04^{ab}	0.94±0.04^{c}	1±0.03^{bc}	1.08±0.07^{a}
HN (g/kg)	0.07±0.01	0.07±0.00	0.07±0.01	0.08±0.01	0.07±0.00	0.07±0.01	0.07±0.00	0.07±0.00
IP (g/kg)	0.71±0.02	0.72±0.05	0.73±0.06	0.78±0.06	0.71±0.08	0.71±0.10	0.75±0.09	0.80±0.03
OP (g/kg)	0.16±0.01^{ab}	0.16±0.02^{ab}	0.14±0.02^{ab}	0.13±0.01^{ab}	0.13±0.02^{b}	0.17±0.03^{a}	0.13±0.01^{b}	0.15±0.02^{ab}
AP (g/kg)	0.01±0.00^{b}	0.01±0.00^{b}	0.01±0.00^{b}	0.02±0.00^{a}	0.02±0.00^{a}	0.02±0.00^{a}	0.01±0.00^{b}	0.02±0.00^{a}
RAK (g/kg)	0.12±0.01	0.12±0.02	0.11±0.01	0.13±0.02	0.15±0.01	0.13±0.02	0.11±0.01	0.13±0.02
SAK (g/kg)	0.86±0.03^{bc}	0.89±0.08^{bc}	0.82±0.01^{c}	0.97±0.10^{ab}	1.03±0.09^{a}	0.96±0.14^{ab}	0.89±0.02^{bc}	0.87±0.04^{bc}
TS (g/kg)	0.38±0.03^{ab}	0.32±0.06^{bc}	0.39±0.03^{a}	0.37±0.05^{ab}	0.39±0.01^{a}	0.34±0.01^{abc}	0.30±0.05^{c}	0.34±0.02^{abc}
AS (g/kg)	0.04±0.01^{c}	0.05±0.01^{c}	0.08±0.02^{b}	0.12±0.01^{a}	0.01±0.00^{d}	0.01±0.00^{d}	0.01±0.00^{d}	0.04±0.00^{c}
SWC (%)	0.15±0.00^{b}	0.16±0.01^{b}	0.16±0.01^{b}	0.22±0.03^{a}	0.14±0.01^{b}	0.10±0.01^{c}	0.08±0.03^{c}	0.09±0.00^{c}
EC (ms/cm)	0.21±0.02^{cde}	0.27±0.06^{bc}	0.29±0.04^{b}	0.38±0.07^{a}	0.19±0.01^{de}	0.18±0.01^{e}	0.17±0.02^{e}	0.26±0.05^{bcd}
pH	8.30±0.10^{a}	8.22±0.04^{ab}	8.22±0.10^{ab}	8.13±0.12^{b}	8.16±0.04^{ab}	8.23±0.09^{ab}	8.24±0.04^{ab}	8.11±0.11^{b}
ALP (μmol/d/g)	11.54±1.18^{ab}	11.64±1.40^{ab}	10.61±1.24^{bc}	9.36±1.12^{c}	12.85±0.96^{a}	12.97±0.77^{a}	12.26±0.92^{ab}	11.33±0.93^{ab}
LAP (μmol/d/g)	47.17±2.76^{a}	39.72±2.70^{bc}	38.54±2.73^{bc}	43.78±4.18^{ab}	40.08±1.41^{bc}	38.04±2.73^{c}	37.01±2.45^{c}	41.57±4.29^{bc}
AGC (μmol/d/g)	23.06±2.27^{b}	19.71±1.89^{bc}	21.81±2.61^{bc}	31.95±3.05^{a}	21.74±2.75^{bc}	18.28±2.28^{c}	21.47±2.82^{bc}	22.00±1.86^{bc}
BGC (μmol/d/g)	25.13±3.24^{bc}	23.43±2.43^{bc}	26.10±3.12^{ab}	29.73±2.20^{a}	20.88±0.85^{cd}	21.31±2.96^{cd}	23.12±3.21^{bcd}	18.64±2.42^{d}
NAG (μmol/d/g)	32.86±3.75	31.69±2.00	32.32±1.07	33.35±2.24	32.76±1.73	31.45±1.90	30.34±1.47	32.42±2.49
BXYS (μmol/d/g)	0.65±0.16	0.71±0.46	0.90±0.10	0.84±0.30	0.69±0.40	0.94±0.10	0.64±0.46	0.55±0.11
PPO (mg/d/g)	50.51±2.15^{c}	68.30±7.43^{b}	55.86±5.44^{c}	47.53±2.80^{c}	56.44±7.06^{c}	78.89±2.40^{a}	69.78±5.87^{ab}	54.39±6.76^{c}
POD (mg/d/g)	81.14±7.58^{cd}	90.45±7.38^{abcd}	79.23±6.51^{d}	92.92±4.69^{abc}	83.85±4.99^{bcd}	99.43±6.35^{a}	89.73±9.95^{abcd}	95.64±8.03^{ab}
ASF (μmol/d/g)	15.23±1.51^{a}	11.83±1.10^{b}	12.88±0.95^{b}	12.01±1.15^{b}	11.52±1.20^{bc}	11.51±0.14^{bc}	12.67±1.22^{b}	9.70±0.96^{c}
BCL (μg /min /g)	57.25±0.28^{b}	57.16±0.91^{b}	58.69±2.78^{ab}	57.93±4.29^{ab}	61.12±1.29^{a}	59.32±0.13^{ab}	58.82±1.56^{ab}	60.45±1.51^{ab}

Letters after numbers indicate significant differences (Tukey-Kramer HSD test; $P < 0.05$; mean ± standard error).

Abbreviations: XNA: Xiong'an New Area; C: Chinese pine tree; S: Chinese scholar tree; SP: spring; SU: summer; AT: autumn; and WI: winter; SWC, Soil water content; TC, total carbon; EC, soil electrical conductivity, SOM, soil organic matter, TK, total potassium, RAK, rapidly available potassium, SAK, slow-available potassium, TP, total phosphorus, TN, total nitrogen, HN, soil hydrolyzed nitrogen, IP, inorganic phosphorus; OP, organic phosphorus, AP, available phosphorus, TS, total sulfur; AS, available sulfur; BGC, β-Glucosidase; BXYS, β- xylosidase; BCL, β-cellobiosidase; LAP, Leucine Aminopeptidase; AGC, α-Glucosidase; NAG, N-acetyl-β-D-glucosidase; ALP, Alkaline phosphatase; ASF, Arylsulfatase; PPO, Polyphenol oxidase; and POD, Peroxidase.

Supplementary Tables

Results showed that winter was characterized by low activities of those enzymes involved in P, ALP ($F_{7, 23}$ = 3.6566, P = 0.0151), S cycling, and ASF ($F_{7, 23}$ = 6.1483, P = 0.0013). More detailed results can be found in the Supporting Text. The association of edaphic factors with EEAs/EES is shown in Figs. S2-S3.

Supplementary Tables

Table S8: The C:N ratio for different seasons

Seasons	CP	CS
Spring	0.32±0.05a	0.29±0.02b
Summer	0.33±0.04a	0.31±0.03ab
Autumn	0.37±0.03a	0.34±0.05a
Winter	0.39±0.04a	0.25±0.02b

Table S9: The C:P ratio for different seasons

Seasons	CP	CS
Spring	2.21±0.53b	1.63±0.12a
Summer	2.05±0.45b	1.65±0.20a
Autumn	2.46±0.12ab	1.90±0.36a
Winter	3.21±0.44a	1.64±0.15a

Table S10: The N:P ratio for different seasons

Seasons	CP	CS
Spring	6.97±0.75b	5.69±0.40b
Summer	6.18±0.56b	5.37±0.48b
Autumn	6.72±0.46b	5.51±0.34b
Winter	8.26±0.36a	6.53±0.11a

Table S11: The C:P:N ratio for different seasons

Seasons	CP	CS
Spring	3.22: 4.38: 2.44	3.04: 4.29: 2.55
Summer	3.15: 4.27: 2.45	3.05: 4.24: 2.56
Autumn	3.26: 4.26: 2.36	3.13: 4.21: 2.50
Winter	3.39: 4.34: 2.23	2.92: 4.30: 2.43
Average value	3.25: 4.31: 2.37	3.04: 4.26: 2.51

Supplementary Tables

Table S12: The relative abundance of microbial phyla and classes in different plantation types and different seasons

	CSP	CSU	CAT	CWI	SSP	SSU	SAT	SWI
**Bacterial phyla**								
Actinobacteria	5012±274bc	5023±657bc	5051±33bc	6030±1080ab	6614±247a	5739±543abc	4873±915c	5561±244bc
Proteobacteria	5245±775	5504±1073	5224±918	5404±1279	5143±650	5126±1158	4158±212	5125±1377
Acidobacteria	3269±1037ab	2978±1314ab	3550±1086ab	2371±296b	2201±506b	2767±614b	4528±1213a	2798±1293b
Chloroflexi	2180±182ab	2173±345ab	2252±299ab	1699±403b	1997±407ab	2463±545a	2331±86a	2004±394ab
Firmicutes	809±197bc	803±142bc	537±88cd	1462±1667a	656±88cd	521±33d	685±247cd	1063±196b
Gemmatimonadetes	620±73bc	802±172a	713±99ab	488±75c	716±90ab	859±66a	695±100ab	736±64ab
**Bacterial classes**								
Actinobacteria	5012±274bc	5023±657bc	5051±33bc	6030±1080ab	6614±247a	5739±543abc	4873±915c	5561±244bc
Alphaproteobacteria	2978±585ab	2941±665ab	2911±ab	3571±1018a	3089±411ab	2833±527ab	2258±315b	3064±873ab
Subgroup-6	2070±842ab	1786±926ab	2277±839ab	1091±652b	1288±350b	1672±486ab	3020±993a	1711±967ab
Gammaproteobacteria	1480±106	1585±287	1539±394	1179±206	1286±181	1499±442	1119±135	1237±301
**Fungal phyla**								
Ascomycota	36323±1123bcd	34878±1123cd	32198±1819d	44971±2674a	39880±2169abc	35311±3822cd	37192±8889bcd	42629±2922ab
Mortierellomycota	5924±1792a	4901±635ab	6031±1792a	1230±898c	4955±1449ab	6784±1774a	4777±2385ab	2876±1548bc
Basidiomycota	4832±98ab	4318±938ab	6638±5818a	3857±1619ab	2349±857ab	2095±1397ab	4073±4214ab	1724±853b
**Fungal classes**								
Sordariomycetes	20607±2002bc	17613±1731c	18380±2387c	31057±4241a	19889±4740c	15599±2152c	19559±9078c	29327±8274ab
Dothideomycetes	8916±1243bc	10146±567b	6407±1964d	14200±1510a	9951±1348b	6856±581cd	8007±1566bcd	7158±931cd
Mortierellomycetes	5879±630a	4819±24ab	6024±1797a	1730±219c	4944±1440ab	6640±1828a	4758±2383ab	2872±1547bc
Pezizomycetes	5282±1118b	5283±2423b	3765±1227bc	1545±345c	2916±2332bc	2726±240bc	8458.50±1733a	1968±1181c
**Protistan phyla**								
Cercozoa	4299±539a	3517±937ab	3772±713a	2148±593b	3486±636ab	2147±677b	3229±683ab	2272±1318b
Chlorophyta	956±508ab	1067±486ab	579±175b	2396±1074a	1443±1128ab	1459±1504ab	1929±910ab	1938±1167ab
Apicomplexa	744±804b	976±722b	2042±1750ab	1301±1260ab	1220±873ab	3163±1006a	1021±618b	1177±616b
Unclassified Eukaryota	1141±402	1583±409	953±88	1649±634	1133±523	1017±247	1538±425	984±110
Lobosa	1145±235b	1152±308b	1777±160a	810±385bc	940±426bc	861±243bc	796±83bc	395±198c
**Protistan classes**								
Filosa-Sarcomonadea	3639±527a	2790±720ab	3186±641a	1504±615c	3046±671ab	1727±633bc	2537±570abc	1864±1033bc
Chlorophyceae	1024±242cd	638±38cd	492±144d	2705±446a	1289±1138bcd	466±43d	1765±798abc	2361±623ab
Apicomplexa-X	651±395b	906±296b	801±5b	1208±398b	1220±873b	4171±419a	992±125b	1456±541b
Unclassified Eukaryota	827±41d	1528±409abc	1158± 202abcd	1654±21a	1133±523abcd	1017±247bcd	1538±425ab	984±110cd
Tubulinea	916±248ab	920±253ab	1443±238a	395±119bc	721±417bc	638±270bc	806±318bc	290±44c
Variosea	360±54bc	880±380ab	358±158c	508±24bc	739±438abc	502±198bc	1068±347a	556±132bc

Supplementary Tables

Table S13: α-diversity indices of soil microbiomes in different plantations with seasonal variations

	CSP	CSU	CAT	CWI	SSP	SSU	SAT	SWI
**Bacteria**								
Sobs	2524±3a	2399±155ab	2288±35b	2250±191b	2239±42b	2298±106b	2355±122ab	2361±29ab
Chao1	3560±55a	3427±256ab	3265±133ab	3236±199b	3185±153b	3265±158ab	3293±226ab	3346±145ab
ACE	3592±84	3649±619	3463±416	3234±237	3390±405	3446±208	3331±201	3350±120
Simpson	0.004±0.001b	0.004±0.001b	0.004±0.001b	0.016±0.013a	0.006±0.001b	0.004±0.001b	0.003±0.000b	0.005±0.001b
Shannon	6.7±0.02a	6.6±0.13a	6.6±0.04ab	6.2±0.23c	6.4±0.05b	6.6±0.11ab	6.6±0.09a	6.6±0.02ab
PD	229±2a	217±16ab	210±2b	208±20b	204±3b	207±9b	214±8ab	214±7ab
**Fungi**								
Sobs	801±127ab	872±76ab	898±49ab	533±86c	715±32bc	912±200a	743±111ab	769±98ab
Chao1	922±159a	976±132a	1009±38a	667±101b	805±23ab	981±240a	838±116ab	889±109ab
ACE	908±170a	977±133a	1017±42a	673±114b	804±26ab	982±233a	846±119ab	896±100ab
Simpson	0.028±0.004b	0.036±0.016b	0.041±0.020b	0.137±0.068a	0.042±0.011b	0.025±0.006b	0.103±0.101ab	0.146±0.091a
Shannon	4.5±0.08ab	4.4±0.23ab	4.3±0.40abc	3.0±0.49d	4.2±0.12abc	4.7±0.25a	3.8±0.81bc	3.6±0.66cd
PD	239±33ab	269±15ab	274±10a	145±27c	219±11b	279±53a	238±38ab	236±35ab
**Protists**								
Sobs	689±49a	584±66b	591±57b	697±681a	648±6ab	598±29b	661±45ab	653±64ab
Chao1	790±75ab	651±89c	678±61bc	827±74a	760±19abc	680±44bc	725±54abc	770±72ab
ACE	781±87ab	634±83d	654±61d	843±63a	734±2abcd	669±48cd	723±61bcd	776±61abc
Simpson	0.014±0.003b	0.013±0.003b	0.071±0.100ab	0.025±0.009b	0.020±0.004b	0.107±0.055a	0.014±0.005b	0.033±0.028b
Shannon	5.2±0.13a	5.2±0.07a	4.7±0.95ab	4.9±0.28ab	5.0±0.04a	4.2±0.32b	5.3±0.13a	4.7±0.66ab
PD	120±5ab	102±12cd	98±9d	123±4a	102±3cd	99±4d	112±4bc	105±2cd

Table S14: Bacterial ANOSIM analysis

Group	Statistic	P value
CSP-CSU	0.0741	0.487
CSP-CAT	0.0741	0.519
CSP-CWI	0.4444	0.098
CSP-SSP	0.7037	0.098
CSP-SSU	0.4444	0.098
CSP-SAT	0.4815	0.098
CSP-SWI	0.3333	0.201
CSU-CAT	-0.1111	0.676
CSU-CWI	0.6296	0.098
CSU-SSP	0.5556	0.098
CSU-SSU	0.1111	0.478
CSU-SAT	0.0741	0.195
CSU-SWI	0.2963	0.408
CAT-CWI	0.5185	0.098
CAT-SSP	0.9259	0.098
CAT-SSU	0.2222	0.3
CAT-SAT	0.4074	0.098
CAT-SWI	0.4815	0.098
CWI-SSP	0.7047	0.098
CWI-SSU	0.8519	0.098
CWI-SAT	0.7037	0.098
CWI-SWI	0.4074	0.098
SSP-SSU	0.2963	0.298
SSP-SAT	0.7037	0.098
SSP-SWI	-0.037	0.407
SSU-SAT	0.3333	0.098
SSU-SWI	0.2222	0.296
SAT-SWI	0.1481	0.293

Supplementary Tables

Table S15: Fungal ANOSIM analysis

Group	R value	P value
CSP-CSU	0.1111	0.511
CSP-CAT	0.3704	0.098
CSP-CWI	0.7037	0.098
CSP-SSP	0.3333	0.098
CSP-SSU	0.5556	0.098
CSP-SAT	0.4815	0.098
CSP-SWI	0.7407	0.098
CSU-CAT	0	0.511
CSU-CWI	0.6667	0.098
CSU-SSP	0.2963	0.186
CSU-SSU	0.1481	0.186
CSU-SAT	-0.0741	0.798
CSU-SWI	0.5926	0.098
CAT-CWI	1	0.098
CAT-SSP	0.8148	0.098
CAT-SSU	0.3704	0.098
CAT-SAT	0.3333	0.098
CAT-SWI	0.963	0.098
CWI-SSP	0.7778	0.098
CWI-SSU	1	0.098
CWI-SAT	0.7407	0.098
CWI-SWI	0.5926	0.098
SSP-SSU	0.4074	0.098
SSP-SAT	0.2593	0.195
SSP-SWI	0.5926	0.098
SSU-SAT	-0.1481	0.786
SSU-SWI	0.7778	0.098
SAT-SWI	0.1852	0.19

Supplementary Tables

Table S16: Protistan ANOSIM analysis

Group	R value	P value
CSP-CSU	0.037	0.622
CSP-CAT	0.1852	0.195
CSP-CWI	0.4074	0.201
CSP-SSP	-0.0556	0.511
CSP-SSU	0.4074	0.098
CSP-SAT	0.5556	0.098
CSP-SWI	0.2222	0.298
CSU-CAT	-0.037	0.616
CSU-CWI	0.1852	0.201
CSU-SSP	-0.0741	0.622
CSU-SSU	0.2593	0.408
CSU-SAT	0.037	0.489
CSU-SWI	0.1852	0.298
CAT-CWI	0.4815	0.098
CAT-SSP	0.2222	0.098
CAT-SSU	0.4074	0.098
CAT-SAT	0.4074	0.098
CAT-SWI	0.4074	0.098
CWI-SSP	0.2963	0.311
CWI-SSU	0.2222	0.208
CWI-SAT	0.1852	0.305
CWI-SWI	-0.1481	0.8
SSP-SSU	0.1852	0.414
SSP-SAT	0.037	0.496
SSP-SWI	-0.1481	0.908
SSU-SAT	0.2593	0.296
SSU-SWI	0.037	0.599
SAT-SWI	0.1111	0.201

Supplementary Tables

Table S17: Bacterial PERMANOVE analysis based on environmental factors

Characteristics	R2	Variance	P-value
SE	0.26831	26.83%	0.029
TR	0.04464	4.46%	0.347
AGC	0.17402	17.40%	0.011
AP	0.17021	17.02%	0.016
SWC	0.14357	14.36%	0.033
TS	0.12883	12.88%	0.045
RAK	0.12456	12.46%	0.053
EC	0.12106	12.11%	0.054
AS	0.11493	11.49%	0.07
SAK	0.08487	8.49%	0.128
ALP	0.08166	8.17%	0.132
TP	0.07747	7.75%	0.133
IP	0.07021	7.02%	0.184
LAP	0.06964	6.96%	0.185
pH	0.07444	7.44%	0.163
SOM	0.07069	7.07%	0.191
POD	0.06696	6.70%	0.203
PPO	0.0681	6.81%	0.203
HN	0.05975	5.98%	0.236
TC	0.06048	6.05%	0.235
ASF	0.05215	5.22%	0.267
TK	0.04939	4.94%	0.282
TN	0.02576	2.58%	0.606
BCL	0.0244	2.44%	0.602
OP	0.02351	2.35%	0.633
BGC	0.01696	1.70%	0.762
BXYS	0.01224	1.22%	0.856
NAG	0.00517	0.52%	0.945

Table S18: Fungal PERMANOVE analysis based on environmental factors

Characteristics	R2	Variance	P-value
SE	0.43295	43.30%	0.002
TR	0.05526	5.53%	0.268
TP	0.35569	35.57%	0.003
AGC	0.28619	28.62%	0.002
IP	0.30436	30.44%	0.002
AP	0.28765	28.77%	0.006
RAK	0.27331	27.33%	0.002
SOM	0.22763	22.76%	0.01
pH	0.14998	15.00%	0.026
POD	0.14901	14.90%	0.041
SAK	0.13744	13.74%	0.06
LAP	0.12998	13.00%	0.057
HN	0.11365	11.37%	0.073
EC	0.12899	12.90%	0.077
PPO	0.11109	11.11%	0.079
AS	0.10252	10.25%	0.089
TK	0.09531	9.53%	0.103
SWC	0.07626	7.63%	0.177
ALP	0.07453	7.45%	0.16
TS	0.07001	7.00%	0.175
TN	0.06866	6.87%	0.21
ASF	0.05914	5.91%	0.25
OP	0.04394	4.39%	0.344
BXYS	0.03385	3.39%	0.437
BGC	0.02993	2.99%	0.455
TC	0.02788	2.79%	0.524
BCL	0.01735	1.74%	0.688
NAG	0.01528	1.53%	0.732

Supplementary Tables

Table S19: The Protistan PERMANOVE analysis based on environmental factors

Characteristics	R2	Variance	P-value
SE	0.17905	17.91%	0.163
TR	0.03879	3.88%	0.466
AGC	0.04739	4.74%	0.357
AP	0.09959	9.96%	0.061
SWC	0.006	0.60%	0.978
TS	0.0138	1.38%	0.873
RAK	0.03428	3.43%	0.545
EC	0.01202	1.20%	0.911
AS	0.00947	0.95%	0.95
SAK	0.015	1.50%	0.871
ALP	0.0286	2.86%	0.611
TP	0.0324	3.24%	0.566
IP	0.0375	3.75%	0.482
LAP	0.01947	1.95%	0.823
pH	0.04724	4.72%	0.345
SOM	0.07297	7.30%	0.147
POD	0.12183	12.18%	0.029
PPO	0.06179	6.18%	0.222
HN	0.01768	1.77%	0.834
TC	0.01271	1.27%	0.893
ASF	0.10625	10.63%	0.05
TK	0.02289	2.29%	0.731
TN	0.05294	5.29%	0.288
BCL	0.03193	3.19%	0.591
OP	0.03205	3.21%	0.576
BGC	0.03728	3.73%	0.491
BXYS	0.04467	4.47%	0.392
NAG	0.08201	8.20%	0.106

Supplementary Tables

Table S20: Topological indices of each network in Fig. 6

	CP	CS	SP	SU	AT	WI
Bacteria (%)	23.81%	24.49%	28.79%	28.57%	25.45%	25.53%
Fungi (%)	11.90%	10.20%	15.15%	10.20%	10.91%	10.64%
Protists (%)	4.76%	14.29%	16.67%	14.29%	16.36%	10.64%
Environmental variables (%)	59.52%	51.02%	39.39%	46.94%	47.27%	53.19%
Network nodes (ND)	42	49	66	49	55	47
Network edges (NG)	77	81	186	108	114	125
Average degree (AD)	3.667	3.306	5.636	4.408	4.145	5.319
Average weighted degree	2.559	2.325	4.951	3.902	3.689	4.762
Average path length (APL)	4.003	4.029	3.760	3.611	3.488	3.249
Radius	1	5	1	1	2	4
Network diameter (ND)	11	9	10	9	9	7
Graph density	0.089	0.069	0.087	0.092	0.077	0.116
Modularity (ML)	0.545	0.65	0.537	0.607	0.667	0.617
Average clustering coefficient (ACC)	0.5	0.364	0.581	0.463	0.559	0.548
Sum changes	0.003704	0.010185	0.008611	0.008206	0.008246	0.014316
Total triangles	51	29	199	93	106	123
Edges linking protists to bacteria (%)	3.90%	4.94%	8.60%	3.70%	2.63%	4.80%
Edges linking protists to fungi (%)	0	0	3.23%	0.93%	1.75%	0.00%
Edges linking bacteria to fungi (%)	0	4.94%	7.53%	6.48%	3.51%	8.00%

In order to determine the linkages of soil biota with environmental variables among different plantations and seasonal dynamics, six networks were constructed by combining all microbiomes from the four seasons and two plantation types. Based on the network size parameter, the constructed co-occurrence network covered 77 (CP with 42 nodes), 81 (CS with 49), 186 (spring with 66 nodes), 108 (summer with 49 nodes), 114 (autumn with 55 nodes), 125 (winter with 47 nodes) edges, including positive and negative relationships.

Supplementary Tables

Table S21: Top 10 nodes with the highest betweenness centrality (BC) value for different plantation types seasonally

ID	Microbial taxa/edaphic factors/ soil enzymes	B.C.	Seasons
Chrysophyceae_Clade-C	Protists	600	Spring
Vermamoebidae	Protists	561	
Sandonidae	Protists	525	
OP	Edaphic factor	492.856157	
BGC	Soil enzyme (C cycle)	474.964356	
pH	Edaphic factor	371.656067	
Norank Actinobacteria	Bacteria	338	
Burkholderiaceae	Bacteria	303.896135	
No-rank KD4-96	Bacteria	177	
ALP	Soil enzyme (P cycle)	167.782313	
AS	Edaphic factor	265.274839	Summer
TK	Edaphic factor	225.893021	
Gemmatimonadaceae	Bacteria	184.910278	
Norank Actinobacteria	Bacteria	145.498234	
SAK	Edaphic factor	145.498234	
Sphingomonadaceae	Bacteria	122.042509	
Nitrosomonadaceae	Bacteria	122.042509	
HN	Edaphic factor	118.075455	
Micrococcaceae	Bacteria	114.127062	
No-rank Subgroup-6	Bacteria	94.297491	
OP	Edaphic factor	185.49281	Autumn
HN	Edaphic factor	135	
IP	Edaphic factor	129.802406	
Nocardioidaceae	Bacteria	128.539194	
BXYS	Soil enzyme (C cycle)	110.539194	
SOM	Edaphic factor	106.939169	
TC	Edaphic factor	92.633333	
Nolandellidae	Protists	70	
TS	Edaphic factor	69.379853	
Paracercomonadidae	Protists	69.366667	
LAP	Soil enzyme (N cycle)	322.192615	Winter
EC	Edaphic factor	235.932318	
SAK	Edaphic factor	216.705457	
Cladosporiaceae	Fungi	165.246855	
BXYS	Soil enzyme (C cycle)	146.548008	
Nocardioidaceae	Bacteria	145.809961	
Sphingomonadaceae	Bacteria	133.13267	
JG30-KF-CM45	Bacteria	107.026422	
Cercomonadidae	Protists	103.390276	
Nectriaceae	Fungi	89.55464	
TP	Edaphic factor	116.116529	Chinese pine tree

Cercomonadidae	Protists	90.006991	
Chlamydomonadales-X	Protists	87.350852	
NorankSubgroup-6	Bacteria	79.001923	
Sphingomonadaceae	Bacteria	73.451324	
Gemmatimonadaceae	Bacteria	71.083257	
AP	Edaphic factor	70.43765	
SWC	Edaphic factor	58.354458	
Sandonidae	Protists	55.327989	
POD	Soil enzyme (Phenolic compounds oxidase)	41.822575	
LAP	Soil enzyme (N cycle)	254.866198	Chinese scholar tree
RAK	Edaphic factor	189.370455	
HN	Edaphic factor	176.164177	
Nitrosomonadaceae	Bacteria	174.795382	
Bacillaceae	Bacteria	171.408211	
Allapsidae	Protists	159.57197	
Nocardioidaceae	Bacteria	144.314719	
Unclassified Fungi	Fungi	138.775047	
Oxytrichidae	Protists	131.041919	
IP	Edaphic factor	119.380961	

More details about co-occurrence networks

In spring, nodes with high degree values were largely associated with the fungal family of Pseudeurotiaceae, the protistan family of Cercomonadidae, the bacterial family of Geminicoccaceae, and AS (environmental factor) (Fig. 5A). In summer, nodes with high degree values were mainly affiliated with AS (environmental factor), the bacterial family of Xanthobacteraceae (Fig. 5B). Comparing autumn and winter, nodes with high degrees were primarily connected with environmental factors, such as RAK, LAP, and ASF in autumn (Fig. 5C), as well as, LAP, HN, pH, and POD (Fig. 5D). However, in CP, nodes with high degrees were mostly linked with TP, IP, and AP (Fig. 5E). In CS, nodes with high degrees were largely affiliated with AS, the bacterial family of Nocardioidaceae, and EC (Fig. 5F). Summer characterized with those nodes were related to bacterial network like Gemmatimonadaceae, Sphingomonadaceae, Nitrosomonadaceae, and unclassified Actinobacteria as well as edaphic factors like AS, TK, SAK,

and HN; autumn characterized with those nodes were belonged to edaphic factors and hydrolysis enzymes, which were belonged to the C cycle, and winter characterized with those key nodes were affiliated to bacterial, fungal and protistan networks, edaphic factors and hydrolysis enzymes, which were belonged to N and C cycles. The biotic key nodes for the *Pinus* forest were affiliated to bacteria and protist networks, whereas these nodes for the *Sophora* forest belonged to fungi, bacteria, and protist networks.

Supplementary Text

Table S22: Network structure for soil microbial interaction at different plantation types seasonally

Groups	Total Positive correlation	Total Negative correlation	Positive Microbe-Microbe interactions	Negative Microbe-Microbe interactions	Positive Environment-Microbe interactions	Negative Environment-Microbe interactions	Positive Environment-Environment interactions
Spring	99	86	30	30	44	43	25
Summer	62	46	14	18	21	30	11
Autumn	77	37	13	11	32	16	32
Winter	79	54	15	19	26	17	38
CP type	61	51	6	9	22	30	33
CS type	62	46	12	13	28	24	22

Microbial interactions were also inferred using co-occurrence network analysis. Correspondingly, the total correlation number, including both positive (co-presence) and negative (mutual exclusion) correlations, was higher in spring. Both plant species outnumbered with the approximately highest number of total positive correlation numbers, whereas the highest number of total negative correlations were recorded for the CP plot.

Supplementary Text

Results

Extracellular ecoenzymatic activities (EEAs) and stoichiometry (EES)

The only significant enzyme production observed for C cycle-related enzymes between season variations and plantations belonged to the BGC activity ($F_{7, 23}$ = 5.13, P = 0.0033), with the highest value in cold seasons in the CP plot. Two other C cycle-related enzyme activities were not significant across seasonal variations [BXYS ($F_{7, 23}$ = 0.6351, P = 0.7209) and BCL ($F_{7, 23}$ = 1.4582, P = 0.2507)]. There was a significant change in two of the N-related enzyme activity, LAP ($F_{7, 23}$ = 33.7595, P = 0.00149) and AGC ($F_{7, 23}$ = 8.2333, P = 0.0003), whereas the NAG activity did not differ between seasons and plantations ($F_{7, 23}$ = 0.5614, P = 0.7762). The activities of oxidative enzymes like PPO ($F_{7, 23}$ = 12.1724, $P < 0.0001$) and POD ($F_{7, 23}$ = 3.0017, P = 0.0325) significantly differed among seasons and plantations. The activities of PPO and POD showed fluctuations during seasonal variations, with the greatest activity occurring in summer in the CS plot (Table S7).

The results of Spearman's heatmap revealed that different enzymes belonging to a particular element cycling has different association with soil nutrients. For instance, BGC showed a positive association with TC, whereas BCL showed a negative association with TC. Interestingly, none of those enzymes related to N cycling showed a significant association with TN, whereas they showed a positive association with TC (Fig. S2). Our study showed significant associations between BGC: LAP + NAG and BGC: ALP with TC/TN ratio (Fig. S3). The results of Spearman's heatmap revealed that soil LAP enzyme showed the highest number of significant and positive associations (n = 8) with edaphic parameters such as TC (R = 0.41, P = 0.04), SOM (R = 0.47, P = 0.02), TP (R = 0.46, P = 0.02), HN (R = 0.57, P = 0.004), IP (R = 0.46, P = 0.02), RAK (R = 0.45, P = 0.003), SWC (R = 0.42, P = 0.03) and EC (R = 0.46, P = 0.02),

whereas ALP showed only one positive association with TK (R = 0.55, P = 0.005) (Fig. S2). The soil enzyme PPO showed the highest number of the negative association (n = 4) with some soil factors like TC (R = -0.41, P = 0.04), AS (R = -0.67, P = 0.0004), SWC (R = -0.54, P = 0.006) and EC (R = -0.54, P = 0.007), whereas BCL and BXYS enzymes showed only one negative association with TC (R = -0.55, P = 0.005) and TN (R = -0.414, P = 0.044), respectively.

Relationship between diversity, abundance, and EEAs

A few soil factors showed significant and similar effects on the Shannon diversity of the two microbial groups. For instance, TP showed significant and negative correlations with bacterial and fungal Shannon diversity. In contrast, AP showed a substantial and adverse link with bacterial and protists Shannon diversity (Fig. 3). Both bacterial and fungal abundance (top 10 phyla) were notably related to various soil factors. Still, protists' abundance showed a contrasting tendency concerning edaphic factors (Fig. 3).

Drivers of soil microbiome community variations

Among all edaphic parameters and soil enzymes, five soil properties were significantly associated with the relative abundance of the dominant bacterial phyla, namely, TK (R^2 = 0.29, P = 0.029), AP (R^2 = 0.39, P = 0.014), RAK (R^2 = 0.51, P = 0.003), SAK (R^2 = 0.41, P = 0.005) and TS (R^2 = 0.29, P = 0.031) (Fig. 3B). Moreover, we found that edaphic parameters such as SOM (R^2 = 0.31, P = 0.025), TP (R^2 = 0.50, P = 0.002), IP (R^2 = 0.42, P = 0.005), AP (R^2 = 0.39, P = 0.009), RAK (R^2 = 0.40, P = 0.004), AS (R^2 = 0.45, P = 0.004), and soil enzymes such as ALP (R^2 = 0.44, P = 0.004), AGC (R^2 = 0.59, P = 0.001), PPO (R^2 = 0.34, P = 0.011) and POD (R^2 = 0.31, P = 0.015) significantly influenced fungal community structures (Figs. 4C, D). In bacterial RDA, the first two axes of soil properties and soil enzyme explained 44.15% and 52.16% of the total

variations, respectively (Figs. 4A, B). In fungal RDA, the first two axes of soil properties and soil enzymes explained 53.56% and 69.79% of the total variations, respectively (Figs. 4C, D). In protists, RDA, the first two axes of soil properties and soil enzyme explained 43.21% and 45.27% of the total variation, respectively (Figs. 4E, F). The RDA displayed those environmental factors correlated with the protistan community, but just the POD enzyme (R^2 = 0.38, P = 0.011) and AP (R^2 = 0.26, P = 0.039) significantly affected the shape of the protistan community (Figs. 4E, F).

Effect of seasonal variations and plantations on microbial trophic groups

Plantation type and seasonality significantly influenced the community structure of PICRUSt2-derived protist potential metabolic pathways; of the top 13 metabolic KEGG and MetaCyc pathways of protistan lineages, 'Adenosine triphosphatase' and 'Aerobic respiration I' were the most abundant pathways among eight groups, which their frequency was statistically distinguished from other pathways (Figs. S14A, B). In terms of fungal guilds, 24 groups were identified as well as an extra group as 'unknown'. The variations for fungal functional guilds were observed seasonally among and within sampling plots (Fig. S15). Symbiotrophs were the most abundant fungal trophic mode in the CP plot in spring and summer (Fig. S16). TAX4FUN analysis showed that six bacterial functional groups were dominated across seasonal samples. The ‘metabolism group’ with the highest number of pathways (n = 11) was distinguished among other groups. Within this group, the abundance of two ‘carbohydrate and amino acid metabolisms’ pathways was the highest compared with other pathways (Fig. S17).

Discussion

Microbial diversity and abundance under seasonal variations and different plantations

Supplementary Text

We found an interaction effect between the abundance of the microbe-consuming protists, Cercozoa, which is globally one of the most abundant and functionally essential soil protists groups (Guo et al., 2021), with pH level and the ASF enzyme activity (Fig. 3). These two environmental variables were significantly dropped from spring to winter at *Pinus* Forest but not at *Sophora* Forests (Table S7). These results suggest that those driving forces governing the diversity and composition of protistan lineages can be different from those governing the abundance of dominated protist lineages, which are widely distributed across habitats, and play a crucial role in maintaining the multifunctionality and stability of a community. This study supports evidence from a previous observation (Walden et al., 2021), highlighting the seasonal variation of the protistan Cercozoa. As another example, the relative abundance of Chloroflexia was significantly correlated with ALP, and AGC enzyme activities (Fig. 3), suggesting the important role of oligotrophic bacteria in contributing to the phosphorus and nitrogen cycles of the ecosystem through mineralization of organic phosphorus and starch degradation, respectively (Yang et al., 2021). In this study, pH had a significant positive effect on N-related functions (Fig. S2). This association may be caused by pH significantly affecting substrate availability (Kemmitt et al., 2006).

Here, we showed that both bacterial and fungal communities were influenced by soil pH, but the bacterial community was more significantly affected by pH than that of fungi, which might be due to comparatively narrow optimal pH ranges for bacterial growth but wide pH ranges for fungal growth, which could potentially alter the microbial community structure by favoring high-pH adapted or alkaliphilic microorganisms. However, PERMANOVA analysis showed that the impact of different plantations significantly explained none of the microbial β-diversity variations. This finding was consistent with that of Liu et al. (2020), who highlighted that the effect of soil type on

shaping bacterial rhizosphere was stronger than plant species. The effect of a possible role of aboveground factors like different plantations was observed on belowground traits like edaphic parameters. For example, there were no seasonal variations for TC, and SOM in *Pinus* Forest, whereas significant seasonal variations were observed for both factors in *Sophora* Forest. This observation indicated the existence of a link between plant species' aboveground and belowground traits. This distinct pattern between soil properties of two different plantations might be due, in part, to differences in rhizodeposition stoichiometry and/or rhizosphere priming (Yin et al., 2014). Our LDA bar results showed how combined variations of soil variables and plant species within seasonal variations could specify specific microbial taxa as biomarkers. This significant specification of specific microbial taxa as bio-indicators in each experimental plot suggested a core group of microbial taxa that responds to seasonal variations in different plantations harmoniously. We should mention that those taxa altered in abundance concerning seasonal variations may simultaneously respond to several soil factors like temperature, soil moisture, and other edaphic parameters. However, identified seasonal taxa could be used in future studies as indicator taxa of potential climatic changes across a wide range of soil types. In temperate forests, microbial communities can be shaped mainly by several seasonal processes, such as seasonal variations in environmental conditions like temperature, moisture, seasonal input of fresh litter, and photosynthates' allocation into soils (López-Mondéjar et al., 2015).

The relative abundance of bacterial taxa was disproportionately higher than other soil biotas

There are several possible explanations for the high proportion of bacterial taxa in afforested plots. The first is that the accumulation of the aboveground litter in the young manmade forest floor in the XNA (forest age stand~ 4 years) is much less than in hyperdiverse and layered

forests (mature temperate forests) yearly, resulting in a low level of ongoing decomposition process through soil microbial networks. Although the role of bacteria in the decay of dead plant biomass is essential (López-Mondéjar et al., 2015), the conventional view has highlighted the role of fungi in the decomposition of plant litter due to their acclimatization, including the possession of an efficient enzymatic apparatus and the ability to translocate nutrients filamentous growth (Brabcová et al., 2016). Our result was consistent with the previous study of Nacke et al. (2014), highlighting that bacterial taxa dominated soil biota in grassland and forest soils. Also, our previous study based on different land-use types in the XNA showed that bacterial and protistan taxa outnumbered fungal lineages (Wang et al., 2021). This result was inconsistent with other forest soil eukaryotic transcriptionally in which the abundance of fungal taxa was higher than bacteria (Bailly et al., 2007; Damon et al., 2012; Takasaki et al., 2013). It has been highlighted that the winter period without photosynthate flow is expected to show increased activity of decomposer microbial species (Baldrian et al., 2012). In line with this concept, we realized that the abundance of some saprophytic fungal genera in *Pinus* Forest, such as *Cryptococus*, *Rhodotorula* was higher than those fungal genera like *Cladophialophora* in *Sophora* Forest (mostly in winter); this might be related to the fact that coniferous litters are composed of recalcitrant organic polymer, resulting in a higher amount of fungal biomass and the proliferation of root-symbiotic fungi; this helps to access to plant-produced C (Root-supplied C). Additionally, our study did not capture those fungal saprophytic taxa like *Mycena*, *Mycosphaerella*, and *Naevala* reported by Žifčáková et al. (2016) in which they described the detailed role of microbial taxa in the *Picea abies*-dominated coniferous forest soil's functioning in two contrasting seasons (summer vs. winter), indicating the pivotal importance of plant litters decomposition to determine certain microbial taxa. Thus, perhaps, the lack of plant litters

reduced saprophytic taxa and enriched non-saprophytic taxa in man-made forest soils. We strongly speculate that the lack of a diverse array of decomposer microbial taxa caused by the paucity of plant litters increased the soil carbon pools in winter in our experimental plots and perhaps influenced fungal abundance. It seems that microbial structures in a young man-made seasonal forest do not follow the general pattern of soil biota of hyperdiverse forests (mature). Therefore, the abundance of saprophytic taxa is high in winter (no photosynthetic plant production), and the high abundance of ectomycorrhizal taxa in summer. Therefore, we speculated that microbial communities and their activities inhabiting the young man-made forest soils might mainly be fueled by rhizodeposition relative to litter decomposition due to the plant litter's paucity, suggesting the nature of soil substrate processing soil microbes may magnify its importance in the overall man-made forest ecosystem. However, therefore, further work needs to be done to validate the role of plant litters in shaping microbial interactions and diversity in the man-made seasonal forest. Second, the pH level in the XNA is alkaline (8.1~ 8.3, Table 1) (Wang et al., 2021). Other studies highlighted that alkaline pH was not optimal for fungal growth (Rousk et al., 2009). Also, it has been highlighted that most protistan taxa prefer alkaline pH (Oliverio et al., 2020). The pH level might considerably explain the lower abundance of fungi in our experimental plots. However, we are not suggesting that soil pH is the leading environmental variable to consider as a universal predictor of the relative abundance of soil microbiomes in our sampling sites. Our GAM analysis showed a positive association between bacterial Shannon diversity and the alkaline pH level, in which Shannon diversity increased from pH 8.0 to 8.4 linearly (Fig. S11). It has been well shown that neutral pH contributes significantly to the diversity of bacterial communities in soils (Fierer and Jackson, 2006), but this does not mean bacterial taxa cannot adjust themselves to alkaline soil conditions.

Supplementary Text

Additionally, this result indicated that pH alone might not play a prominent role in bacterial diversity and stability in soils (Fierer and Jackson, 2006). Third, the fungal growth network is more sensitive to anthropogenic activities than other soil biotas (Strickland and Rousk, 2010; Wang et al., 2021) through creating manmade forests. Perhaps the reasons mentioned above could lead to the fact that the relative fungal abundance disproportionately to be lower than other soil biotas, and it is emphasized that bacterial-dominated ecosystem would presumably link to increased nitrogen losses and reduced C-sequestration (Strickland and Rousk, 2010; de Vries et al., 2011; de Vries et al., 2013). Here, we showed that both bacterial and fungal communities were influenced by soil pH, but the bacterial community was more significantly affected by pH than that of fungi, which might be due to comparatively narrow optimal pH ranges for bacterial growth but wide pH ranges for fungal growth, which could potentially alter the microbial community structure by favoring high-pH adapted or alkaliphilic microorganisms.

Our results showed that the high proportion of assigned reads and quantitative PCR belonged to bacterial taxa in our experimental plots in the plantation forest over the course of one year. Some plausible reasons might describe this observation. First, the annual accumulation of aboveground litter in the young plantation forest floor in the XNA (forest stand age ~ 4 years) is much lower than that in hyperdiverse and layered forests (mature temperate forests). Second, other studies highlighted that alkaline pH was not optimal for fungal growth (Rousk et al., 2009). Unlike fungal diversity indices without an association with soil pH, bacterial Shannon diversity was significantly associated with soil pH (Figure 3), suggesting that the bacterial diversity was more significantly affected by pH than the fungal diversity, which might be due to comparatively narrow optimal pH ranges for bacterial growth but wide pH ranges for fungal growth (Rousk et al., 2010). Third, the fungal growth network is more sensitive to

anthropogenic activities (Strickland and Rousk, 2010; Wang et al., 2021) and forest management (Bastida et al., 2017) than the bacterial community by creating plantation forests. Our results suggest that the development of the new forest may have resulted in a loss of niches for other soil biotas. Moreover, we infer that the microbial community structure can be altered by the potential effect of alkalinity by favoring high-pH-adapted or alkaliphilic microorganisms. We concluded that several reasons, such as the pH level, the paucity of plant litterfall, anthropogenic activities, and land management practices, created an opportunity for bacterial taxa to take over other soil biotas, such as fungal and protist lineages in young plantation, seasonal, open-canopy forests in the XNA. Further studies that take these variables into account will need to be undertaken.

Our results showed that microbial communities were significantly bacterial-dominated genera in spring and summer. In contrast, the autumn and winter communities were fungal-dominated genera. Still, these fungal differences were not significant (Figure 1). Previous studies that evaluated the seasonal fluctuations of the microbial community in diverse ecosystems (Schadt et al., 2003; Bardgett et al., 2005; Schmidt et al., 2007) observed consistent results on whether fungi that dominated under-snow biomass and bacterial taxa were more active in summer. The increase in bacterial abundance from spring to summer was associated with an increased abundance of protist genera, some of which may be bacterial feeders (Figure 1). Several reasons can explain the domination of bacterial taxa in summer. First, fungi commonly target recalcitrant substrates such as plant litter (higher C to N ratio), whereas bacteria generally target labile substrates such as root exudates. In summer, warmer temperatures can increase root exudates compared with winter, and fresh plant litter input produces a relatively more moderate C to N ratio (Strickland and Rousk, 2010). Therefore, the domination of bacterial taxa was

expected in summer. Another reason that can explain the trend mentioned above might be related to temperature and soil moisture as the two main factors that drive soil biota abundance (Brockett et al., 2012; Wei et al., 2020). We realized that the soil moisture was relatively high in winter, particularly in the *Pinus* forest (Table S7). Additionally, the GAM analysis showed that both fungal and bacterial Shannon diversity indices decreased with increasing soil moisture. The decrease in the bacterial Shannon diversity was much greater than that of the fungal Shannon diversity (Figure S10), suggesting a threshold for soil moisture, and those fungi will exhibit less of a response to changes in moisture compared with bacteria (Strickland and Rousk, 2010). However, it has been highlighted that the bacterial community was more sensitive to lower soil moisture than the fungal community (Bastida et al., 2017). A consistent pattern for the effect of soil moisture on soil biota abundance remains to be elucidated. Another possible explanation for the increased abundance of fungi in cold seasons could be partially related to their natural resistance to freeze-thaw perturbations (McMahon et al., 2009; Haei et al., 2011) and may not be related to substrate preference. More discussion about these reasons can be found in the Supporting Text.

Soil microbial co-occurrence network complexity under seasonal successions and in different plantations

As bacteria and fungi are principal prey for phagotrophic and/or consumer protists as the dominant soil protist functional group in soils (Saleem et al., 2012; Friman et al., 2016; Geisen et al., 2016; Oliverio et al., 2020; Guo et al., 2021), biotic interactions within the soil microbiome can influence protist diversity. The taxon-specific manner of protist taxa was observed for their potential microbial prey by detecting different and/or specific links between bacteria/fungi and protist lineages. These results are consistent with previous studies showing

that protist taxa selectively graze on fungal or bacterial lineages (Geisen et al., 2016; Schulz-Bohm et al., 2017; Zhao et al., 2019). Interestingly, however, we observed changeable connections of some protist groups with different microbiome taxa in different seasons and different plantations, suggesting that the substitution of preferable feeding impacts the predator composition structure (Saleem et al., 2012). The network results showed that the young plantation forests induced more positive interactions (mutualism or commensalism) than negative interactions (competitive) in each plantation type and even in different seasons, suggesting that cooperative interactions might play a key role in shaping microbial interactions and structures in different plantations seasonally. This might correspond to stable ecosystem functioning (Wang et al., 2021). However, competitive interactions do not necessarily match unstable and/or poor ecosystem functioning (L. Qiu et al., 2021). Whether these positive and negative interactions mutually impact the microbial network assembly seasonally needs to be further assessed. The association of environmental variables with co-occurrence network parameters (Figures 5 and S14) showed that the assembly of the ecological network was shaped by several vital parameters, among which temperature appeared to be the strongest. This striking observation is consistent with the previous study, highlighting that microbial co-occurrence networks are mainly modulated by temperature, precipitation, soil nitrogen, latitude, and plant diversity (Tu et al., 2020).

Effect of different plantations and seasons on soil enzymes

It has been shown that there is a yearly succession of dominant substrates for microbial growth progressing; for instance, the availability of carbon polymers/phenolics within winter, proteins during snowmelt time, and rhizodeposition within summer (Schmidt et al., 2007). This fact might be a reasonable explanation for the high level of TC and SOM in winter in our experimental plot,

but all the parts of these nutrients might not be available for microbial access, and consequently, lead to the higher activity of some enzymes like POD in winter. The local climatic condition and temporal variability might be other parameters to alter the microbial composition structure (Fu et al., 2020). With respect to environmental variables, we found a positive link between BGC: LAP + NAG, BGC: ALP, and LAP + NAG: ALP ratios with soil moisture (Fig. S2), suggesting the importance of soil moisture on soil enzyme production. More specifically, regional climate is expected to influence soil microbial compositions differently based on a determination of both moisture conditions and temperature as the main driving factors of seasonal differences in microbial compositions (Baldrian et al., 2013; Žifčáková et al., 2017). Consequently, it would impact the pools of various ecoenzymes activities, including hydrolytic enzymes involved in biogeochemical cycling (C, N, P, and S cycling), and oxidative enzyme activities (Sinsabaugh, 2010; Brockett et al., 2012; Jia et al., 2017; Xu et al., 2017; Zheng et al., 2020). The association of temperature variation during the year with a single function (soil enzyme activities) was previously observed in the tundra (Schadt et al., 2003; Wallenstein et al., 2009), in a temperate spruce forest, and in a boreal coniferous forest (Wittmann et al., 2004). Besides, precipitation is a crucial factor in tropical and subtropical zones in comparison with temperature due to the lower variations of temperature than those in cold and temperate zones (Ahmed et al., 2019). Previous studies demonstrated that higher climatic parameters like soil moisture might increase the EEA (Brockett et al., 2012; Jia et al., 2017; Xu et al., 2017; Zheng et al., 2020). Our results suggest that the abundance of oligotrophic (microbial communities like Acidobacteria as a slow-growing group) non-significantly dropped, and the abundance of copiotrophic communities (Bacteroidetes and most Proteobacteria; faster-growing) non-significantly increased in the *Pinus* Forest with the

higher soil moisture (Fig. S5; Tables S12), suggesting bacterial taxonomy was shifted through soil moisture and consequently higher EEA activities seasonally.

Our oxidative activities measurement, particularly for POD, showed a strong and segregated seasonal pattern in which the enzyme concentration was high in winter (relatively with higher soil moisture) and low in summer (Table S7), suggesting the vital role of seasonality and consequently soil moisture on soil oxidative activities. Our results support evidence from a previous observation (Di Nardo et al., 2004), which emphasized that the impact of seasonal variations on oxidative activities with evaluating associated oxidative activities with decomposing *Quercus ilex* litter (oak) in Mediterranean shrubland and other observations (Laiho, 2006; Sinsabaugh, 2010), in which noted that oxidative activities were precipitously dropped during dry seasons. Our results showed that *Sophora* Forest faced a more substantial P limitation (with an averagely lower TP) than the *Pinus* Forest, with comparatively lower C:P and N:P ecoenzyme activity ratios, particularly in warmer seasons (Tables S8-11). This result is similar to Hou et al. (2020), highlighting that P deficiency is a common problem in forest ecosystems and often intensifies in the summer season. However, the abundance of P cycling genes in both plots did not show a significant difference (Fig. 8D). We should mention that extracellular enzyme activities have not been explicitly represented in global biogeochemical models because the relationship between EEA and microbial functional genes is unclear. This association is not straightforward (Sinsabaugh and Shah, 2012). Our result might indicate that when microbes faced a low level of P availability, they accelerated the expression of P-acquiring enzymes and decelerated the expression of C and N-acquiring enzymes (Sinsabaugh et al., 2009; Tapia-Torres et al., 2015), thereby resulting in a lower C:P and N:P ecoenzyme activity ratios. Other studies used identical interpretations for C:P and N:P activity ratios to conclude P

limitation in different ecosystem types (Waring et al., 2014; Peng and Wang, 2016; Zhang et al., 2019; Zheng et al., 2020). We should mention that *Sophora* Forest was characterized by bigger plants and perhaps with more extensive root systems compared with *Pinus* Forest, expecting to provide more exogenous resources to microbes in the form of above- and belowground litter input for the synthesis of soil EEAs (Zhang et al., 2019), and expected to see higher C:N and N:P ratios, but it was out of the expectation. Thus, the limitation of P availability in the soil is most likely due to plant uptake and immobilization of P in plant biomass (Zhang et al., 2016). Additionally, based on the average ratio of C:N:P acquisition enzymes in forest types (Table S9), the primarily smaller P than C or N-acquiring enzyme activities were observed. Therefore, as we observed through obtaining the ecoenzymatic activity ratios, land-use changed to a forest, and different plantations increased microbial P limitation, and perhaps microbes allocated more resources to obtain P by increasing ALP activity non-significantly, especially within spring and summer (Table S7) (Zheng et al., 2020), indicating P is a critical constraining nutrient for man-made forests in the XNA.

Detailed discussion for CNPS cycling genes

Our findings suggest that very different profiles of CNPS cycling genes in our experimental plots with a significantly lower abundance of C-degradation, C-fixation, N and S cycling in Chinese pine plots in autumn and winter than that in Chinese scholar tree plots, suggesting that seasonality (Landesman et al., 2019), and plantation (Chen et al., 2020) through alteration of soil microbial communities overwhelmingly influenced the balance of biogeochemical cycles. Our study showed a significant association between oxidative activities and the composition of protistan lineages. Lignin degradation may indicate that microbes resort to mining N from more recalcitrant organic matter pools due to severely N-limited conditions (Moorhead and Sinsabaugh, 2006; Broadbent et

al., 2017; Broadbent et al., 2021). Due to severely N-limited conditions, lignin degradation may indicate that microbes are restoring to mining N from more recalcitrant organic matter pools. We also detected the absolute abundance of genes involved in nitrification (*amoA1*, *nxrA*, *amoA2*, *amoB*, and *hao*), particularly the higher abundance of *amoA1* in summer in *Pinus* Forest. The increasing importance of *amoA1* likely reflects the combination of lignin-degrading enzymes and a legacy of nitrification following in summer in the specific plantation. Thus, our findings suggested that soil microbial communities contribute mainly to the breakdown of labile organic compounds (hemicellulose) in the year-round study irrespective of seasonality matched with other C-related compounds like starch, cellulose, chitin, pectin, and lignin (Fig. S20). This discrepancy might be a good reason why the potential activity of those enzymes involved in C-hydrolysis degradation, like BCL, BXYS, BCL, and LAP was relatively stable annually, irrespective of plantation types with seasonal variations (Table S7). Our result showed that the abundance of C-hydrolysis genes involved in hemicellulose degradation, *abfA* (arabinofuranosidase), *xylA* (xylose isomerase), and *manB* (mannanase), was higher than other C-hydrolysis genes irrespective of plantation types and seasonal variations. Such trends were attributed mainly to the fact that in the young afforested areas, the organic matter pool might mainly comprise labile (simple) substrates because of fewer plant inputs (litters and roots). Furthermore, the concentration of β-Xylosidase enzyme (hydrolysis of hemicellulose) was not significantly different in different plots with seasonal variations, suggesting stable degradation of plant-derived components, and soil microbes are the primary consumers of labile substrates in afforested plots in young man-made forests. Ren et al. (2021) reported that bacterial phyla like *Actinobacteria*, *Proteobacteria*, and *Acidobacteria*, can access C in cellulose, hemicelluloses, and chitin by producing the variety of extracellular enzymes, which were dominated bacterial phyla in our study irrespective of plantation types.

Supplementary Text

Interestingly, the abundance of microbial taxa like fungal order Thelebolales and bacterial orders Actinomycetales and Bacillales were very low in our experimental plots, and the close relationship of them with cellulose degradation is also reported by other studies (Lynd et al., 2002; Zhou et al., 2014; Bhatnagar et al., 2018; Broadbent et al., 2021). This association might be a possible explanation for the lower abundance of those genes involved in cellulose degradation. The significantly positive correlation between the abundance of CNPS cycling genes with microbial diversity indices suggests microbial diversity and composition can affect microbial functions (extracellular enzymes and functional genes). More specifically, the result of RDA and the positive association of microbial diversity indices with CNPS cycling genes suggest that protistan community composition can potentially shape by C-related functions like C-degradation genes and C-oxidative related enzymes. We conclude that in young man-made forests in the XNA with less plant input, the structure of soil organic matter mostly is labile, which the abundance of those genes involved in C cycling like hemicellulose was higher than other genes. Also, the soil is alkaline, which is a good encouragement for lignin-degrading enzymes. Subsequently, protistan community composition can be shaped by the C-related functions (C-cycling genes and oxidative-related enzymes), irrespective of plantation types and seasonal variations, whereas bacterial and fungal community composition can be shaped differently.

Supplementary Text

Effect of seasonality on soil enzymes

The results of the ecoenzyme activities indicated that seasonal variations and plantation types significantly influenced the EEA (Table S7), indicating different isoenzyme pools and, consequently, the domination of different microbial taxa in each season, plantation, gene pool, and expression pattern. Other researchers documented comparable seasonal alterations in the EEA of soils (Chen et al., 2016; Machmuller et al., 2016; Sherman and Coleman, 2020; X. Qiu et al., 2021). As an example, the production of BGA increased in winter in the *Pinus* forest, whereas its activity was high in autumn in the *Sophora* forest. A possible explanation is that different plantation types have individual ecological and physiological features (Zhang et al., 2018), resulting in different effects on the edaphic conditions and microenvironment (Zhang et al., 2019), conclusively impacting the secretion of microbial nutrient-acquiring exoenzymes and plants (Cui et al., 2018). High bacterial and fungal gene copy numbers generally showed higher enzyme activity in the *Pinus* forest than in the *Sophora* forest, indicating that key microbial-mediating biogeochemical processes vary seasonally in different plantations (Fig. S11). Additionally, these higher gene copy numbers likely explain the high potential enzyme activities in winter, but our oxidative enzyme activities were higher in summer. A similar finding showed that oxidative activities were more variable than hydrolytic activities and increased with soil pH (Sinsabaugh et al., 2008). In addition, as mentioned previously, microbes typically invest more in enzymes, especially lignin-degrading enzymes, in summer (Broadbent et al., 2021). In contrast, potential enzyme activities were potentially expected to be high in winter. The most recent study highlighted that the same EEA dataset could be interpreted in contrasting ways (Fierer et al., 2021). Higher enzyme activities can be interpreted as more nutrient availability (Sinsabaugh et al., 2008) or reduced nutrient availability (Nannipieri et al., 2012). In accordance with the resource allocation strategy, soil biota

may secrete more soil ecoenzymes under low nutrient conditions (Sinsabaugh et al., 2002; Wallenius et al., 2011; Sinsabaugh and Shah, 2012; Bowles et al., 2014; Cenini et al., 2016; Xu et al., 2017; Jing Wang et al., 2020; Zhou et al., 2020). In our study, we inferred that the higher activity of some enzymes in cold seasons might be related to the lack of plant litter and plant photosynthesis products. However, other studies showed that higher enzyme activities in hyperdiverse forests were limited to autumn, which provided fresh and readily available substrates for more secretion of soil ecoenzymes (Wittmann et al., 2004; Baldrian et al., 2008; X. Qiu et al., 2021), suggesting the synchronous intensification of belowground biomass and plant litter driving a considerable increase in the metabolism of microorganisms to produce more soil ecoenzymes. Our study showed the positive relationships of BGC: ALP, LAP + NAG: ALP with TC, LAP + NAG with TP and SOM (Fig. S2), as well as significant associations between BGC: LAP + NAG and BGC: ALP with TC/TN ratio (Fig. S2); these associations might not be the conclusion to match enzymatic acquisition ratios with nutrient stoichiometry (Zhou et al., 2020) because nutrients such as organic matter elements can be stored in various forms, and only a tiny portion can be used by microbes (Kamble and Bååth, 2014).

The results of the investigated C:N:P stoichiometry ratios indicated a deviation from the global (Sinsabaugh et al., 2008; 2012) and regional scale of 1:1:1 for China's forests based on a nationwide dataset (Xu et al., 2017; Zhou et al., 2020), suggesting that ecoenzyme activity stoichiometry in the plantation forests of the XNA was mostly based on nutrient resource availability and demand for microbial nutrients and was not homeostatic. This result is consistent with that from several studies conducted in China (Peng and Wang, 2016; Zhang et al., 2019; Jie Wang et al., 2020), indicating that this ratio can potentially be different based on the type of ecosystem and soil. Our results also showed that the *Sophora* forest faced more substantial P

limitation (with an average lower TP) than the *Pinus* forest, with comparatively lower C:P and N:P ecoenzyme activity ratios, particularly in warmer seasons (Tables S9-S10). This is similar to the previous finding, highlighting that P deficiency is a common problem in forest ecosystems and often intensifies in the summer (Hou et al., 2020). However, the abundance of P cycling genes in both forest types did not show a significant difference (Fig. 6C). A detailed discussion is presented in the Supporting text.

References

Ahmed, I.U., Mengistie, H.K., Godbold, D.L., and Sandén, H., (2019). Soil moisture integrates the influence of land-use and season on soil microbial community composition in the Ethiopian highlands. Applied Soil Ecology 135, 85-90. DOI: https://doi.org/10.1016/j.apsoil.2018.11.010.

Bailly, J., Fraissinet-Tachet, L., Verner, M.-C., Debaud, J.-C., Lemaire, M., Wésolowski-Louvel, M., and Marmeisse, R., (2007). Soil eukaryotic functional diversity, a metatranscriptomic approach. The ISME Journal 1 (7), 632-642. DOI: https://doi.org/10.1038/ismej.2007.68.

Baldrian, P., Kolařík, M., Štursová, M., Kopecký, J., Valášková, V., Větrovský, T., Žifčáková, L., Šnajdr, J., Rídl, J., Vlček, Č., and Voříšková, J., (2012). Active and total microbial communities in forest soil are largely different and highly stratified during decomposition. The ISME Journal 6 (2), 248-258. DOI: https://doi.org/10.1038/ismej.2011.95.

Baldrian, P., Šnajdr, J., Merhautová, V., Dobiášová, P., Cajthaml, T., and Valášková, V., (2013). Responses of the extracellular enzyme activities in hardwood forest to soil temperature and seasonality and the potential effects of climate change. Soil Biology and Biochemistry 56, 60-68. DOI: https://doi.org/10.1016/j.soilbio.2012.01.020.

Baldrian, P., Trögl, J., Frouz, J., Šnajdr, J., Valášková, V., Merhautová, V., Cajthaml, T., and Herinková, J., (2008). Enzyme activities and microbial biomass in topsoil layer during spontaneous succession in spoil heaps after brown coal mining. Soil Biology and Biochemistry 40 (9), 2107-2115. DOI: https://doi.org/10.1016/j.soilbio.2008.02.019.

Bardgett, R.D., Bowman, W.D., Kaufmann, R., and Schmidt, S.K., (2005). A temporal approach to linking aboveground and belowground ecology. Trends in Ecology & Evolution 20 (11), 634-641. DOI: https://doi.org/10.1016/j.tree.2005.08.005.

Bastida, F., Torres, I.F., Andrés-Abellán, M., Baldrian, P., López-Mondéjar, R., Větrovský, T., Richnow, H.H., Starke, R., Ondoño, S., García, C., López-Serrano, F.R., and Jehmlich, N., (2017). Differential sensitivity of total and active soil microbial communities to drought and forest management. Global Change Biology 23 (10), 4185-4203. DOI: https://doi.org/10.1111/gcb.13790.

Behie, S.W., Moreira, C.C., Sementchoukova, I., Barelli, L., Zelisko, P.M., and Bidochka, M.J., (2017). Carbon translocation from a plant to an insect-pathogenic endophytic fungus. Nature Communications 8 (1), 14245. DOI: https://doi.org/10.1038/ncomms14245.

Bhatnagar, J.M., Peay, K.G., and Treseder, K.K., (2018). Litter chemistry influences decomposition through activity of specific microbial functional guilds. Ecological Monographs 88 (3), 429-444. DOI: https://doi.org/10.1002/ecm.1303.

Boerner, R.E.J., Brinkman, J.A., and Smith, A., (2005). Seasonal variations in enzyme activity and organic carbon in soil of a burned and unburned hardwood forest. Soil Biology and Biochemistry 37 (8), 1419-1426. DOI: https://doi.org/10.1016/j.soilbio.2004.12.012.

Supplementary Text

Bowles, T.M., Acosta-Martínez, V., Calderón, F., and Jackson, L.E., (2014). Soil enzyme activities, microbial communities, and carbon and nitrogen availability in organic agroecosystems across an intensively-managed agricultural landscape. Soil Biology and Biochemistry 68, 252-262. DOI: https://doi.org/10.1016/j.soilbio.2013.10.004.

Brabcová, V., Nováková, M., Davidová, A., and Baldrian, P., (2016). Dead fungal mycelium in forest soil represents a decomposition hotspot and a habitat for a specific microbial community. New Phytologist 210 (4), 1369-1381. DOI: https://doi.org/10.1111/nph.13849.

Broadbent, A.a.D., Orwin, K.H., Peltzer, D.A., Dickie, I.A., Mason, N.W.H., Ostle, N.J., and Stevens, C.J., (2017). Invasive N-fixer impacts on litter decomposition driven by changes to soil properties not litter quality. Ecosystems 20 (6), 1151-1163. DOI: https://doi.org/10.1007/s10021-016-0099-3.

Broadbent, A.a.D., Snell, H.S.K., Michas, A., Pritchard, W.J., Newbold, L., Cordero, I., Goodall, T., Schallhart, N., Kaufmann, R., Griffiths, R.I., Schloter, M., Bahn, M., and Bardgett, R.D., (2021). Climate change alters temporal dynamics of alpine soil microbial functioning and biogeochemical cycling via earlier snowmelt. The ISME Journal 15 (8), 2264-2275. DOI: https://doi.org/10.1038/s41396-021-00922-0.

Brockett, B.F.T., Prescott, C.E., and Grayston, S.J., (2012). Soil moisture is the major factor influencing microbial community structure and enzyme activities across seven biogeoclimatic zones in western Canada. Soil Biology and Biochemistry 44 (1), 9-20. DOI: https://doi.org/10.1016/j.soilbio.2011.09.003.

Caporaso, J.G., Lauber, C.L., Walters, W.A., Berg-Lyons, D., Lozupone, C.A., Turnbaugh, P.J., Fierer, N., and Knight, R., (2011). Global patterns of 16S rRNA diversity at a depth of millions of sequences per sample. Proceedings of the National Academy of Sciences 108 (Supplement 1), 4516-4522. DOI: https://doi.org/10.1073/pnas.1000080107.

Cenini, V.L., Fornara, D.A., Mcmullan, G., Ternan, N., Carolan, R., Crawley, M.J., Clément, J.-C., and Lavorel, S., (2016). Linkages between extracellular enzyme activities and the carbon and nitrogen content of grassland soils. Soil Biology and Biochemistry 96, 198-206. DOI: https://doi.org/10.1016/j.soilbio.2016.02.015.

Chen, Q.-L., Ding, J., Zhu, D., Hu, H.-W., Delgado-Baquerizo, M., Ma, Y.-B., He, J.-Z., and Zhu, Y.-G., (2020). Rare microbial taxa as the major drivers of ecosystem multifunctionality in long-term fertilized soils. Soil Biology and Biochemistry 141, 107686. DOI: https://doi.org/10.1016/j.soilbio.2019.107686.

Chen, X., Chen, H.Y.H., Chen, X., Wang, J., Chen, B., Wang, D., and Guan, Q., (2016). Soil labile organic carbon and carbon-cycle enzyme activities under different thinning intensities in Chinese fir plantations. Applied Soil Ecology 107, 162-169. DOI: https://doi.org/10.1016/j.apsoil.2016.05.016.

Criquet, S., Farnet, A.M., Tagger, S., and Le Petit, J., (2000). Annual variations of phenoloxidase activities in an evergreen oak litter: influence of certain biotic and abiotic factors. Soil Biology and Biochemistry 32 (11), 1505-1513. DOI: https://doi.org/10.1016/S0038-0717(00)00027-4.

Cui, Y., Fang, L., Guo, X., Wang, X., Zhang, Y., Li, P., and Zhang, X., (2018). Ecoenzymatic stoichiometry and microbial nutrient limitation in rhizosphere soil in the arid area of the northern Loess Plateau, China. Soil Biology and Biochemistry 116, 11-21. DOI: https://doi.org/10.1016/j.soilbio.2017.09.025.

Damon, C., Lehembre, F., Oger-Desfeux, C., Luis, P., Ranger, J., Fraissinet-Tachet, L., and Marmeisse, R., (2012). Metatranscriptomics reveals the diversity of genes expressed by eukaryotes in forest soils. PloS One 7 (1). DOI: https://doi.org/10.1371/journal.pone.0028967.

De Vries, F.T., Thébault, E., Liiri, M., Birkhofer, K., Tsiafouli, M.A., Bjørnlund, L., Bracht Jørgensen, H., Brady, M.V., Christensen, S., De Ruiter, P.C., D'hertefeldt, T., Frouz, J., Hedlund, K., Hemerik, L., Hol, W.H.G., Hotes, S., Mortimer, S.R., Setälä, H., Sgardelis, S.P., Uteseny, K., Van Der Putten, W.H., Wolters, V., and Bardgett, R.D., (2013). Soil food web properties explain ecosystem services across European land use systems. Proceedings of the National Academy of Sciences of the United States of America 110 (35), 14296-14301. DOI: https://doi.org/10.1073/pnas.1305198110.

De Vries, F.T., Van Groenigen, J.W., Hoffland, E., and Bloem, J., (2011). Nitrogen losses from two grassland soils with different fungal biomass. Soil Biology and Biochemistry 43 (5), 997-1005. DOI: https://doi.org/10.1016/j.soilbio.2011.01.016.

Decker, K.L., Boerner, R.E., and Morris, S.J., (1999). Scale-dependent patterns of soil enzyme activity in a forested landscape. Canadian Journal of Forest Research 29 (2), 232-241. DOI: https://doi.org/10.1139/x98-192.

Di Nardo, C., Cinquegrana, A., Papa, S., Fuggi, A., and Fioretto, A., (2004). Laccase and peroxidase isoenzymes during leaf litter decomposition of *Quercus ilex* in a Mediterranean ecosystem. Soil Biology and Biochemistry 36 (10), 1539-1544. DOI: https://doi.org/10.1016/j.soilbio.2004.07.013.

Fierer, N., and Jackson, R.B., (2006). The diversity and biogeography of soil bacterial communities. Proceedings of the National Academy of Sciences of the United States of America 103 (3), 626-631. DOI: https://doi.org/10.1073/pnas.0507535103.

Fierer, N., Wood, S.A., and Bueno De Mesquita, C.P., (2021). How microbes can, and cannot, be used to assess soil health. Soil Biology and Biochemistry 153, 108111. DOI: https://doi.org/10.1016/j.soilbio.2020.108111.

Finzi, A.C., Sinsabaugh, R.L., Long, T.M., and Osgood, M.P., (2006). Microbial community responses to atmospheric carbon dioxide enrichment in a warm-temperate forest. Ecosystems 9 (2), 215-226. DOI: https://doi.org/10.1007/s10021-005-0078-6.

Friman, V.-P., Dupont, A., Bass, D., Murrell, D.J., and Bell, T., (2016). Relative importance of evolutionary dynamics depends on the composition of microbial predator–prey community. The ISME Journal 10 (6), 1352-1362. DOI: https://doi.org/10.1038/ismej.2015.217.

Fu, D., Wu, X., Qiu, Q., Duan, C., and Jones, D.L., (2020). Seasonal variations in soil microbial communities under different land restoration types in a subtropical mountains region, Southwest China. Applied Soil Ecology 153, 103634. DOI: https://doi.org/10.1016/j.apsoil.2020.103634.

Gardes, M., and Bruns, T.D., (1993). ITS primers with enhanced specificity for basidiomycetes--application to the identification of mycorrhizae and rusts. Molecular Ecology (2), 113-118. DOI: 10.1111/j.1365-294x.1993.tb00005.x.

Geisen, S., Koller, R., Hünninghaus, M., Dumack, K., Urich, T., and Bonkowski, M., (2016). The soil food web revisited: Diverse and widespread mycophagous soil protists. Soil Biology and Biochemistry 94, 10-18. DOI: https://doi.org/10.1016/j.soilbio.2015.11.010.

Guo, S., Xiong, W., Hang, X., Gao, Z., Jiao, Z., Liu, H., Mo, Y., Zhang, N., Kowalchuk, G.A., Li, R., Shen, Q., and Geisen, S., (2021). Protists as main indicators and determinants of plant performance. Microbiome 9, 64. DOI: https://doi.org/10.1186/s40168-021-01025-w.

Haei, M., Rousk, J., Ilstedt, U., Öquist, M., Bååth, E., and Laudon, H., (2011). Effects of soil frost on growth, composition and respiration of the soil microbial decomposer community. Soil Biology and Biochemistry 43 (10), 2069-2077. DOI: https://doi.org/10.1016/j.soilbio.2011.06.005.

Hou, E., Luo, Y., Kuang, Y., Chen, C., Lu, X., Jiang, L., Luo, X., and Wen, D., (2020). Global meta-analysis shows pervasive phosphorus limitation of aboveground plant production in natural terrestrial ecosystems. Nature Communications 11, 637. DOI: https://doi.org/10.1038/s41467-020-14492-w.

Huse, S.M., Dethlefsen, L., Huber, J.A., Welch, D.M., Relman, D.A., and Sogin, M.L., (2008). Exploring Microbial Diversity and Taxonomy Using SSU rRNA Hypervariable Tag Sequencing. PLOS Genetics 4 (11), e1000255. DOI: https://doi.org/10.1371/journal.pgen.1000255.

Jia, X., Shao, M.A., Zhu, Y., and Luo, Y., (2017). Soil moisture decline due to afforestation across the Loess Plateau, China. Journal of Hydrology 546, 113-122. DOI: https://doi.org/10.1016/j.jhydrol.2017.01.011.

Kamble, P.N., and Bååth, E., (2014). Induced N-limitation of bacterial growth in soil: Effect of carbon loading and N status in soil. Soil Biology and Biochemistry 74, 11-20. DOI: https://doi.org/10.1016/j.soilbio.2014.02.015.

Kellner, H., Luis, P., Schlitt, B., and Buscot, F., (2009). Temporal changes in diversity and expression patterns of fungal laccase genes within the organic horizon of a brown forest soil. Soil Biology and Biochemistry 41 (7), 1380-1389. DOI: https://doi.org/10.1016/j.soilbio.2009.03.012.
Kemmitt, S.J., Wright, D., Goulding, K.W.T., and Jones, D.L., (2006). pH regulation of carbon and nitrogen dynamics in two agricultural soils. Soil Biology and Biochemistry 38 (5), 898-911. DOI: https://doi.org/10.1016/j.soilbio.2005.08.006.
Laiho, R., (2006). Decomposition in peatlands: Reconciling seemingly contrasting results on the impacts of lowered water levels. Soil Biology and Biochemistry 38 (8), 2011-2024. DOI: https://doi.org/10.1016/j.soilbio.2006.02.017.
Landesman, W.J., Freedman, Z.B., and Nelson, D.M., (2019). Seasonal, sub-seasonal and diurnal variation of soil bacterial community composition in a temperate deciduous forest. FEMS Microbiology Ecology 95 (2). DOI: https://doi.org/10.1093/femsec/fiz002.
Liu, L., Huang, X., Zhang, J., Cai, Z., Jiang, K., and Chang, Y., (2020). Deciphering the relative importance of soil and plant traits on the development of rhizosphere microbial communities. Soil Biology and Biochemistry 148, 107909. DOI: https://doi.org/10.1016/j.soilbio.2020.107909.
López-Mondéjar, R., Voříšková, J., Větrovský, T., and Baldrian, P., (2015). The bacterial community inhabiting temperate deciduous forests is vertically stratified and undergoes seasonal dynamics. Soil Biology and Biochemistry 87, 43-50. DOI: https://doi.org/10.1016/j.soilbio.2015.04.008.
Lynd, L.R., Weimer, P.J., Van Zyl, W.H., and Pretorius, I.S., (2002). Microbial cellulose utilization: fundamentals and biotechnology. Microbiology and molecular biology reviews : MMBR 66 (3), 506-577, table of contents. DOI: https://doi.org/10.1128/MMBR.66.3.506-577.2002
Machmuller, M.B., Mohan, J.E., Minucci, J.M., Phillips, C.A., and Wurzburger, N., (2016). Season, but not experimental warming, affects the activity and temperature sensitivity of extracellular enzymes. Biogeochemistry 131 (3), 255-265. DOI: https://doi.org/10.1007/s10533-016-0277-6.
Mcmahon, S.K., Wallenstein, M.D., and Schimel, J.P., (2009). Microbial growth in Arctic tundra soil at −2°C. Environmental Microbiology Reports 1 (2), 162-166. DOI: https://doi.org/10.1111/j.1758-2229.2009.00025.x.
Moorhead, D.L., and Sinsabaugh, R.L., (2006). A theoretical model of litter decay and microbial interaction. Ecological Monographs 76 (2), 151-174. DOI: https://doi.org/10.1890/0012-9615(2006)076[0151:ATMOLD]2.0.CO;2.
Nacke, H., Fischer, C., Thürmer, A., Meinicke, P., and Daniel, R., (2014). Land Use Type Significantly Affects Microbial Gene Transcription in Soil. Microbial Ecology 67 (4), 919-930. DOI: https://doi.org/10.1007/s00248-014-0377-6.
Nannipieri, P., Giagnoni, L., Renella, G., Puglisi, E., Ceccanti, B., Masciandaro, G., Fornasier, F., Moscatelli, M.C., and Marinari, S., (2012). Soil enzymology: classical and molecular approaches. Biology and Fertility of Soils 48 (7), 743-762. DOI: https://doi.org/10.1007/s00374-012-0723-0.
Oliverio, A.M., Geisen, S., Delgado-Baquerizo, M., Maestre, F.T., Turner, B.L., and Fierer, N., (2020). The global-scale distributions of soil protists and their contributions to belowground systems. Science Advances 6 (4), eaax8787. DOI: https://doi.org/10.1126/sciadv.aax8787.
Peng, X., and Wang, W., (2016). Stoichiometry of soil extracellular enzyme activity along a climatic transect in temperate grasslands of northern China. Soil Biology and Biochemistry 98, 74-84. DOI: https://doi.org/10.1016/j.soilbio.2016.04.008.
Qiu, L., Zhang, Q., Zhu, H., Reich, P.B., Banerjee, S., Van Der Heijden, M.G.A., Sadowsky, M.J., Ishii, S., Jia, X., Shao, M., Liu, B., Jiao, H., Li, H., and Wei, X., (2021). Erosion reduces soil microbial diversity, network complexity and multifunctionality. The ISME Journal 15 (8), 2474-2489. DOI: https://doi.org/10.1038/s41396-021-00913-1.
Qiu, X., Peng, D., Tian, H., Wang, H., Liu, X., Cao, L., Li, Z., and Cheng, S., (2021). Soil ecoenzymatic stoichiometry and microbial resource limitation driven by thinning practices and season types in *Larix principis-rupprechtii* plantations in North China. Forest Ecology and Management 482, 118880. DOI: https://doi.org/10.1016/j.foreco.2020.118880.

Ren, C., Zhang, X., Zhang, S., Wang, J., Xu, M., Guo, Y., Wang, J., Han, X., Zhao, F., Yang, G., and Doughty, R., (2021). Altered microbial CAZyme families indicated dead biomass decomposition following afforestation. Soil Biology and Biochemistry 160, 108362. DOI: https://doi.org/10.1016/j.soilbio.2021.108362.

Rimington, W.R., Pressel, S., Duckett, J.G., Field, K.J., and Bidartondo, M.I., (2019). Evolution and networks in ancient and widespread symbioses between Mucoromycotina and liverworts. Mycorrhiza 29 (6), 551-565. DOI: https://doi.org/10.1007/s00572-019-00918-x.

Rousk, J., Bååth, E., Brookes, P.C., Lauber, C.L., Lozupone, C., Caporaso, J.G., Knight, R., and Fierer, N., (2010). Soil bacterial and fungal communities across a pH gradient in an arable soil. The ISME Journal 4 (10), 1340-1351. DOI: https://doi.org/10.1038/ismej.2010.58.

Rousk, J., Brookes, P.C., and Bååth, E., (2009). Contrasting soil pH effects on fungal and bacterial growth suggest functional redundancy in carbon mineralization. Applied and Environmental Microbiology 75 (6), 1589-1596. DOI: https://doi.org/10.1128/aem.02775-08.

Saleem, M., Fetzer, I., Dormann, C.F., Harms, H., and Chatzinotas, A., (2012). Predator richness increases the effect of prey diversity on prey yield. Nature Communications 3 (1), 1305. DOI: https://doi.org/10.1038/ncomms2287.

Santos, S.S., Nielsen, T.K., Hansen, L.H., and Winding, A., (2015). Comparison of three DNA extraction methods for recovery of soil protist DNA. Journal of Microbiological Methods 115, 13-19. DOI: https://doi.org/10.1016/j.mimet.2015.05.011.

Santos, S.S., Schöler, A., Nielsen, T.K., Hansen, L.H., Schloter, M., and Winding, A., (2020). Land use as a driver for protist community structure in soils under agricultural use across Europe. Science of The Total Environment 717, 137228. DOI: https://doi.org/10.1016/j.scitotenv.2020.137228.

Schadt, C.W., Martin, A.P., Lipson, D.A., and Schmidt, S.K., (2003). Seasonal dynamics of previously unknown fungal lineages in tundra soils. Science 301 (5638), 1359-1361. DOI: https://doi.org/10.1126/science.1086940.

Schmidt, S., Costello, E., Nemergut, D., Cleveland, C.C., Reed, S., Weintraub, M., Meyer, A., and Martin, A., (2007). Biogeochemical consequences of rapid microbial turnover and seasonal succession in soil. Ecology 88 (6), 1379-1385. DOI: https://doi.org/10.1890/06-0164.

Schulz-Bohm, K., Geisen, S., Wubs, E.R.J., Song, C., De Boer, W., and Garbeva, P., (2017). The prey's scent – Volatile organic compound mediated interactions between soil bacteria and their protist predators. The ISME Journal 11 (3), 817-820. DOI: https://doi.org/10.1038/ismej.2016.144.

Sherman, L., and Coleman, M.D., (2020). Forest soil respiration and exoenzyme activity in western North America following thinning, residue removal for biofuel production, and compensatory soil amendments. GCB Bioenergy 12 (3), 223-236. DOI: https://doi.org/10.1111/gcbb.12668.

Sinsabaugh, R., Carreiro, M., and Alvarez, S. 2002. "Enzyme and microbial dynamics of litter decomposition," in Enzyme and microbial dynamics of litter decomposition, eds. R.G. Burns & R.P. Dick. (New York, USA: Marcel Dekker, Inc., 249-265. DOI: https://doi.org/10.1201/9780203904039.ch9.

Sinsabaugh, R.L., (2010). Phenol oxidase, peroxidase and organic matter dynamics of soil. Soil Biology and Biochemistry 42 (3), 391-404. DOI: https://doi.org/10.1016/j.soilbio.2009.10.014.

Sinsabaugh, R.L., Gallo, M.E., Lauber, C., Waldrop, M.P., and Zak, D.R., (2005). Extracellular enzyme activities and Soil organic matter dynamics for northern hardwood forests receiving simulated nitrogen deposition. Biogeochemistry 75 (2), 201-215. DOI: https://doi.org/10.1007/s10533-004-7112-1.

Sinsabaugh, R.L., Hill, B.H., and Follstad Shah, J.J., (2009). Ecoenzymatic stoichiometry of microbial organic nutrient acquisition in soil and sediment. Nature 462 (7274), 795-798. DOI: https://doi.org/10.1038/nature08632.

Sinsabaugh, R.L., Lauber, C.L., Weintraub, M.N., Ahmed, B., Allison, S.D., Crenshaw, C., Contosta, A.R., Cusack, D., Frey, S., Gallo, M.E., Gartner, T.B., Hobbie, S.E., Holland, K., Keeler, B.L., Powers, J.S., Stursova, M., Takacs-Vesbach, C., Waldrop, M.P., Wallenstein, M.D., Zak, D.R., and Zeglin,

L.H., (2008). Stoichiometry of soil enzyme activity at global scale. Ecology Letters 11 (11), 1252-1264. DOI: https://doi.org/10.1111/j.1461-0248.2008.01245.x.

Sinsabaugh, R.L., Saiya-Cork, K., Long, T., Osgood, M.P., Neher, D.A., Zak, D.R., and Norby, R.J., (2003). Soil microbial activity in a Liquidambar plantation unresponsive to CO2-driven increases in primary production. Applied Soil Ecology 24 (3), 263-271. DOI: https://doi.org/10.1016/S0929-1393(03)00002-7.

Sinsabaugh, R.L., and Shah, J.J.F., (2012). Ecoenzymatic stoichiometry and ecological theory. Annual Review of Ecology, Evolution, and Systematics 43 (1), 313-343. DOI: https://doi.org/10.1146/annurev-ecolsys-071112-124414.

Strickland, M.S., and Rousk, J., (2010). Considering fungal:bacterial dominance in soils – Methods, controls, and ecosystem implications. Soil Biology and Biochemistry 42 (9), 1385-1395. DOI: https://doi.org/10.1016/j.soilbio.2010.05.007.

Takasaki, K., Miura, T., Kanno, M., Tamaki, H., Hanada, S., Kamagata, Y., and Kimura, N., (2013). Discovery of glycoside hydrolase enzymes in an avicel-adapted forest soil fungal community by a metatranscriptomic approach. PloS One 8 (2), e55485. DOI: https://doi.org/10.1371/journal.pone.0055485.

Tapia-Torres, Y., Elser, J.J., Souza, V., and García-Oliva, F., (2015). Ecoenzymatic stoichiometry at the extremes: How microbes cope in an ultra-oligotrophic desert soil. Soil Biology and Biochemistry 87, 34-42. DOI: https://doi.org/10.1016/j.soilbio.2015.04.007.

Toberman, H., Freeman, C., Evans, C., Fenner, N., and Artz, R.R.E., (2008). Summer drought decreases soil fungal diversity and associated phenol oxidase activity in upland Calluna heathland soil. FEMS Microbiology Ecology 66 (2), 426-436. DOI: https://doi.org/10.1111/j.1574-6941.2008.00560.x.

Tu, Q., Yan, Q., Deng, Y., Michaletz, S.T., Buzzard, V., Weiser, M.D., Waide, R., Ning, D., Wu, L., He, Z., and Zhou, J., (2020). Biogeographic patterns of microbial co-occurrence ecological networks in six American forests. Soil Biology and Biochemistry 148, 107897. DOI: https://doi.org/10.1016/j.soilbio.2020.107897.

Ullah, S., Ai, C., Huang, S., Zhang, J., Jia, L., Ma, J., Zhou, W., and He, P., (2019). The responses of extracellular enzyme activities and microbial community composition under nitrogen addition in an upland soil. PloS One 14 (9), e0223026. DOI: https://doi.org/10.1371/journal.pone.0223026.

Wallenius, K., Rita, H., Mikkonen, A., Lappi, K., Lindström, K., Hartikainen, H., Raateland, A., and Niemi, R.M., (2011). Effects of land use on the level, variation and spatial structure of soil enzyme activities and bacterial communities. Soil Biology and Biochemistry 43 (7), 1464-1473. DOI: https://doi.org/10.1016/j.soilbio.2011.03.018.

Wallenstein, M.D., Mcmahon., S.K., and Schimel., J.P., (2009). Seasonal variation in enzyme activities and temperature sensitivities in Arctic tundra soils. Global Change Biology 15 (7), 1631-1639. DOI: https://doi.org/10.1111/j.1365-2486.2008.01819.x.

Wang, C., Masoudi, A., Wang, M., Yang, J., Yu, Z., and Liu, J., (2021). Land-use types shape soil microbial compositions under rapid urbanization in the Xiong'an New Area, China. Science of the Total Environment 777, 145976. DOI: https://doi.org/10.1016/j.scitotenv.2021.145976.

Wang, J., Feng, L., Palmer, P.I., Liu, Y., Fang, S., Bösch, H., O'dell, C.W., Tang, X., Yang, D., Liu, L., and Xia, C., (2020). Large Chinese land carbon sink estimated from atmospheric carbon dioxide data. Nature 586 (7831), 720-723. DOI: https://doi.org/10.1038/s41586-020-2849-9.

Wang, J., Wang, X., Liu, G., Wang, G., Wu, Y., and Zhang, C., (2020). Fencing as an effective approach for restoration of alpine meadows: Evidence from nutrient limitation of soil microbes. Geoderma 363, 114148. DOI: https://doi.org/10.1016/j.geoderma.2019.114148.

Waring, B.G., Weintraub, S.R., and Sinsabaugh, R.L., (2014). Ecoenzymatic stoichiometry of microbial nutrient acquisition in tropical soils. Biogeochemistry 117 (1), 101-113. DOI: https://doi.org/10.1007/s10533-013-9849-x.

Wei, G., Li, M., Shi, W., Tian, R., Chang, C., Wang, Z., Wang, N., Zhao, G., and Gao, Z., (2020). Similar drivers but different effects lead to distinct ecological patterns of soil bacterial and archaeal

communities. Soil Biology and Biochemistry 144, 107759. DOI: https://doi.org/10.1016/j.soilbio.2020.107759.

Weintraub, M.N., Scott-Denton, L.E., Schmidt, S.K., and Monson, R.K., (2007). The effects of tree rhizodeposition on soil exoenzyme activity, dissolved organic carbon, and nutrient availability in a subalpine forest ecosystem. Oecologia 154 (2), 327-338. DOI: https://doi.org/10.1007/s00442-007-0804-1.

White Tj, Bruns Td, Lee Sb, and Jw, T. 1990. Amplification and direct sequencing of fungal ribosomal RNA genes for phylogenetics. United States: Academic Press

Wittmann, C., Kähkönen, M.A., Ilvesniemi, H., Kurola, J., and Salkinoja-Salonen, M.S., (2004). Areal activities and stratification of hydrolytic enzymes involved in the biochemical cycles of carbon, nitrogen, sulphur and phosphorus in podsolized boreal forest soils. Soil Biology and Biochemistry 36 (3), 425-433. DOI: https://doi.org/10.1016/j.soilbio.2003.10.019.

Xu, Z., Yu, G., Zhang, X., He, N., Wang, Q., Wang, S., Wang, R., Zhao, N., Jia, Y., and Wang, C., (2017). Soil enzyme activity and stoichiometry in forest ecosystems along the North-South Transect in eastern China (NSTEC). Soil Biology and Biochemistry 104, 152-163. DOI: https://doi.org/10.1016/j.soilbio.2016.10.020.

Yang, L., Barnard, R., Kuzyakov, Y., and Tian, J., (2021). Bacterial communities drive the resistance of soil multifunctionality to land-use change in karst soils. European Journal of Soil Biology 104, 103313. DOI: https://doi.org/10.1016/j.ejsobi.2021.103313.

Yin, H., Wheeler, E., and Phillips, R.P., (2014). Root-induced changes in nutrient cycling in forests depend on exudation rates. Soil Biology and Biochemistry 78, 213-221. DOI: https://doi.org/10.1016/j.soilbio.2014.07.022.

Zhang, C., Liu, G., Xue, S., and Wang, G., (2016). Soil bacterial community dynamics reflect changes in plant community and soil properties during the secondary succession of abandoned farmland in the Loess Plateau. Soil Biology and Biochemistry 97, 40-49. DOI: https://doi.org/10.1016/j.soilbio.2016.02.013.

Zhang, W., Gao, D., Chen, Z., Li, H., Deng, J., Qiao, W., Han, X., Yang, G., Feng, Y., and Huang, J., (2018). Substrate quality and soil environmental conditions predict litter decomposition and drive soil nutrient dynamics following afforestation on the Loess Plateau of China. Geoderma 325, 152-161. DOI: https://doi.org/10.1016/j.geoderma.2018.03.027.

Zhang, W., Xu, Y., Gao, D., Wang, X., Liu, W., Deng, J., Han, X., Yang, G., Feng, Y., and Ren, G., (2019). Ecoenzymatic stoichiometry and nutrient dynamics along a revegetation chronosequence in the soils of abandoned land and *Robinia pseudoacacia* plantation on the Loess Plateau, China. Soil Biology and Biochemistry 134, 1-14. DOI: https://doi.org/10.1016/j.soilbio.2019.03.017.

Zhao, Z.-B., He, J.-Z., Geisen, S., Han, L.-L., Wang, J.-T., Shen, J.-P., Wei, W.-X., Fang, Y.-T., Li, P.-P., and Zhang, L.-M., (2019). Protist communities are more sensitive to nitrogen fertilization than other microorganisms in diverse agricultural soils. Microbiome 7, 33. DOI: https://doi.org/10.1186/s40168-019-0647-0.

Zheng, H., Liu, Y., Chen, Y., Zhang, J., Li, H., Wang, L., and Chen, Q., (2020). Short-term warming shifts microbial nutrient limitation without changing the bacterial community structure in an alpine timberline of the eastern Tibetan Plateau. Geoderma 360, 113985. DOI: https://doi.org/10.1016/j.geoderma.2019.113985.

Zhou, L., Liu, S., Shen, H., Zhao, M., Xu, L., Xing, A., and Fang, J., (2020). Soil extracellular enzyme activity and stoichiometry in China's forests. Functional Ecology 34 (7), 1461-1471. DOI: https://doi.org/10.1111/1365-2435.13555.

Zhou, Y., Pope, P.B., Li, S., Wen, B., Tan, F., Cheng, S., Chen, J., Yang, J., Liu, F., Lei, X., Su, Q., Zhou, C., Zhao, J., Dong, X., Jin, T., Zhou, X., Yang, S., Zhang, G., Yang, H., Wang, J., Yang, R., Eijsink, V.G.H., and Wang, J., (2014). Omics-based interpretation of synergism in a soil-derived cellulose-degrading microbial community. Scientific Reports 4 (1), 5288. DOI: https://doi.org/10.1038/srep05288.

Supplementary Text

Žifčáková, L., Větrovský, T., Howe, A., and Baldrian, P., (2016). Microbial activity in forest soil reflects the changes in ecosystem properties between summer and winter. Environmental Microbiology 18 (1), 288-301. DOI: https://doi.org/10.1111/1462-2920.13026.

Žifčáková, L., Větrovský, T., Lombard, V., Henrissat, B., Howe, A., and Baldrian, P., (2017). Feed in summer, rest in winter: microbial carbon utilization in forest topsoil. Microbiome 5, 122. DOI: https://doi.org/10.1186/s40168-017-0340-0.
